# Alkynyl-Gold Carbazole Hybrids: Luminescence and Functionalization via iClick Reactions

**DOI:** 10.1021/acs.inorgchem.5c02714

**Published:** 2025-08-20

**Authors:** Roberto Berbés Martínez, Juan V. Alegre-Requena, Raquel P. Herrera, M. Concepción Gimeno

**Affiliations:** † Departamento de Química Inorgánica, Instituto de Síntesis Química y Catálisis Homogénea (ISQCH) CSIC-Universidad de Zaragoza, 50009 Zaragoza, Spain; ‡ Laboratorio de Organocatálisis Asimétrica, Departamento de Química Orgánica, Instituto de Síntesis Química y Catálisis Homogénea (ISQCH) CSIC-Universidad de Zaragoza, C/Pedro Cerbuna 12, 50009 Zaragoza, Spain

## Abstract

A series of structurally diverse gold­(I) complexes bearing the 9-(4-ethynylphenyl)-9*H*-carbazole chromophore were synthesized, featuring mononuclear, dinuclear, tricoordinated, and supramolecular architectures. Their formation involved either alkynylation reactions or the reaction of polymeric alkynyl species [Au­(C≡CR)]_*n*_ with auxiliary ligands. Notably, an equilibrium between bimetallic and tricoordinated species was observed when diphosphine ligands were employed, highlighting the dynamic nature of these systems. This equilibrium was found to be solvent-dependent, with the pure tricoordinated complex isolable in a nonpolar solvent. The triazole complexes were synthesized via iClick chemistry by reacting the carbazole-functionalized alkyne with azide-phosphine gold­(I) precursors. Structural analysis confirmed hydrogen-bonding frameworks and the absence of π–π stacking. Photophysical studies demonstrated intense solid-state phosphorescence, with blue-green luminescence markedly enhanced at 77 K. At room temperature, emission broadened (450–600 nm) and red-shifted compared to the free ligand, indicating a synergistic contribution of carbazole-centered (^3^IL) and intramolecular charge transfer (ICT) transitions. This metal-tuned photophysical behavior underscores the potential of these gold complexes in luminescent materials. This charge transfer, occurring from the carbazole to the ligand system is mediated by the phenyl ring and modulated by metal coordination. TD-DFT calculations were performed to analyze the molecular orbitals involved in both the absorption (S0 → S_1_) and emission (T_1_ → S0) transitions of the complex **1**, indicating that the emission is predominantly of intraligand character, although coordination to the Au­(I) center induces slight modifications in the energy levels and transition intensities.

## Graphical abstract

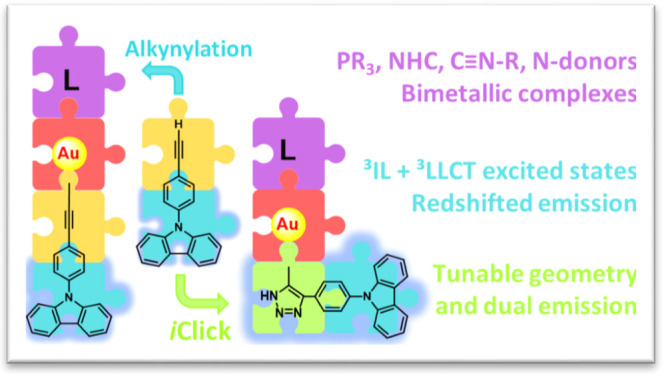

## Introduction

In recent decades, gold­(I) alkynyl complexes have garnered significant attention due to their intriguing structural frameworks^1–4^ and versatile applications in catalysis,^5^ pharmaceuticals,^6–9^ and photochemistry.^10–13^ The combination of the linear coordination geometry of gold­(I) complexes with the inherent linearity and π-conjugation of the alkynyl ligand makes these compounds attractive building blocks for the design of oligomers and polymers.^14,15^

Additionally, alkynyl ligands are recognized as strong σ- and π-donors, and studies using photoelectron spectroscopy and theoretical calculations have confirmed that the Au–C bond in alkynyl gold complexes is among the strongest known gold-ligand bonds.^16^ Its high dissociation energy contributes to the extraordinary stability of these species, highlighting their exceptional potential for application-driven technologies.

Furthermore, the ability of gold atoms to engage in supramolecular interactions, driven by metallophilic and electrostatic forces, enhances their potential for constructing sophisticated molecular architectures.^17–20^ Combined with the ease of functionalizing the alkynyl fragment at both carbon atoms, this allows for the incorporation of functional groups such as chromophores and other biologically or electronically relevant moieties. These characteristics make gold­(I) alkynyl complexes suitable candidates for the development of materials with luminescent properties, organic light-emitting diodes (OLEDs), and molecular electronic devices.^21,22^

The coordination of alkynyl ligands to gold not only preserves their intrinsic properties but can also enhance them through the electronic influence of gold or the unique structural features imparted by the C≡C–Au fragment. This interplay of geometric, electronic, and functional versatility emphasizes the importance of gold­(I) alkynyl complexes in modern material and molecular design.

This study focuses on the synthesis and characterization of gold­(I) complexes featuring the ligand 9-(4-ethynylphenyl)-9*H*-carbazole (**EPC**) in combination with various auxiliary ligands, with an emphasis on investigating their photoluminescent properties. Carbazole-based compounds are integral to the advancement of OLEDs, serving as both emitters and host materials.^23^ Furthermore, these compounds play versatile roles as intermediates in dye synthesis, photocatalysts in organic reactions, and monomers for photoluminescent polymers.^24,25^ By analyzing and comparing the photophysical properties of the free ligand with those of its Au­(I) complexes, this study aims to determine how the gold center influences in the luminescent properties. Finally, the synthetic versatility of alkynyl ligands has been used to perform iClick-type reactions, synthesizing organometallic complexes with triazole fragments attached to the Au­(I) center. These complexes can incorporate different auxiliary ligands depending on the azide used in the click reaction. This approach allows us to study the effect of the formal insertion of a triazole group between the chromophore and the metal center on luminescent properties.

## Results and Discussion

### Synthesis and Characterization of Gold Complexes

The first group of complexes that were synthesized are those with the structure RC≡CAuL, where R = 4-phenyl-9*H*-carbazole and L = different auxiliary ligands. As auxiliary ligands, phosphines of varying steric volumes and NHCs, two of the most commonly used species for forming stable gold complexes, have been studied. Additionally, other less common ligands in gold-alkynyl chemistry, such as isocyanides, imidazoles, and amines, have also been used.

The synthesis of alkynyl gold­(I) complexes was achieved using two distinct methods (Schemes 1 and 2). In the first approach, which has been used for most of the complexes, the alkyne reacts with the gold precursor, [AuCl­(L)], in the presence of a base, as illustrated in Scheme 1. In this reaction, the gold fragment activates the alkyne, facilitating its deprotonation.

**Scheme 1:**
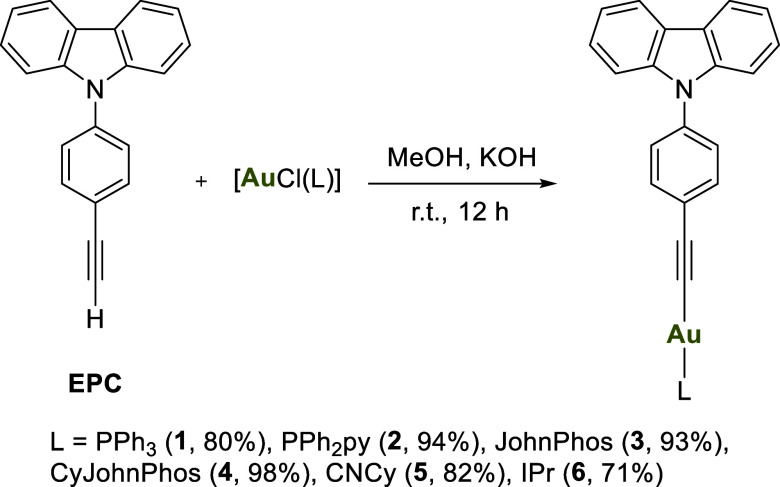
Synthesis of Gold­(I) Alkynyl Complexes **1**–**6**

**Scheme 2:**
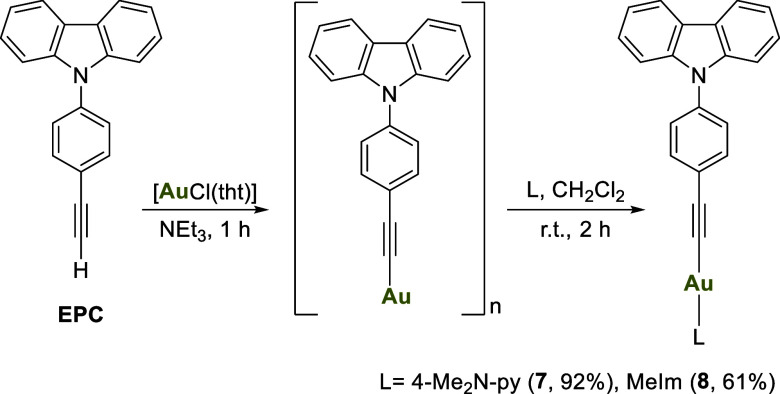
Synthesis of Gold­(I) Species **7** and **8**

All complexes were characterized by ^1^H, ^31^P­{^1^H} and ^13^C­{^1^H}-APT NMR spectroscopy (see Supporting Information, Figures S1–S16), as well as by mass spectrometry. The ^1^H NMR spectra for complexes **1–6** exhibit the expected signals, confirming the presence of both ligands and the absence of the proton of the alkyne precursor. The ^31^P­{^1^H} NMR spectra for the phosphine-containing complexes display a downfield-shifted singlet compared to the starting material, indicating the coordination of the gold fragment. The ^13^C­{^1^H}-APT NMR resonance for the carbene carbon of **6** appears at 191.0 ppm, showing a downfield shift compared to the chloride derivatives (Figure S16). Mass spectrometry analysis reveals molecular ion peaks corresponding to protonated or sodium-adduct species [M + H]^+^ or [M + Na]^+^.

Compounds bearing nitrogen donor atoms as auxiliary ligands could not be synthesized using this method due to issues encountered during the formation of the precursor complexes ([AuCl­(L)]), which could not be efficiently isolated. For their synthesis, a gold-based metallic polymer was used as the precursor compound.

By reacting stoichiometric amounts of [AuCl­(tht)] and 9-(4-ethynylphenyl)-9*H*-carbazole in the presence of an excess of triethylamine, a yellow polymer with the structure [AuC≡CR]_*n*_ (where R = phenyl-9*H*-carbazole) is obtained. These gold alkynyl polymers are known to react with nucleophiles, breaking their polymeric structure and forming the corresponding RC≡CAuNu complex.^26^

Therefore, the remaining compounds were synthesized in dichloromethane using equimolar amounts of the polymer with 4-dimethylaminopyridine for compound **7** and with 1-methylimidazole for compound **8** (Scheme 2).

The characterization of complexes **7** and **8** was performed using similar techniques as those applied to the former compounds. The NMR spectra (Figures S17–S19) display the expected resonances for both ligands with different chemical shifts.

The use of a flexible bidentate diphosphine facilitated the formation of the dinuclear species **9**, whereas a rigid diphosphine, such as XantPhos, enabled the formation of the tricoordinated compound **10** (Scheme 3).

**Scheme 3:**
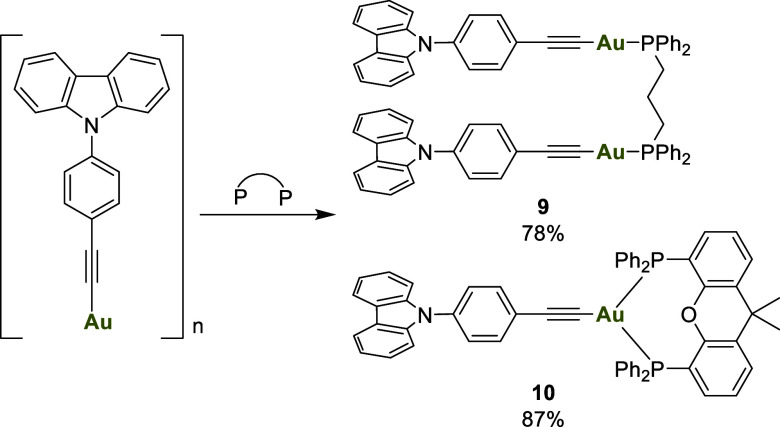
Reaction of the Polymer with Diphosphines

The synthesis of complex **9** was confirmed by NMR spectroscopy. However, the ^31^P­{^1^H} NMR spectrum of complex **10** revealed the presence of a minor impurity. After recrystallization, X-ray diffraction analysis confirmed the formation of an ionic compound, consisting of a homoleptic cationic tetra-coordinated gold phosphine species, with the bis­(alkynyl)gold compound as the anionic counterpart. This indicates the presence of an equilibrium in solution, from which the mixed compound crystallized (Scheme 4).

**Scheme 4:**
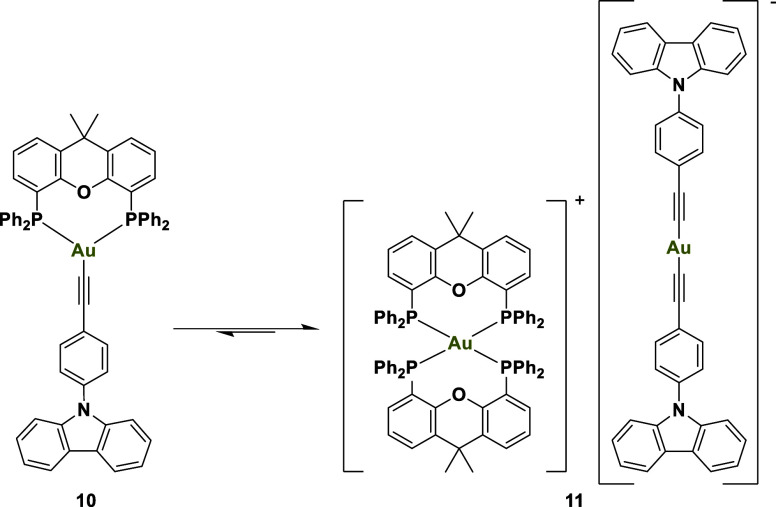
Equilibrium between **10** and **11** in Solution

To explore the conditions that favor the formation of these species, variable-temperature ^31^P­{^1^H} NMR spectroscopy was employed for complex **10** (Figure S32). The goal was to determine whether the observed structure exists solely in solution or if an equilibrium is present between the initially proposed compound and the structure identified by X-ray diffraction. At elevated temperatures, the system possesses enough thermal energy to enable rapid exchange between the different conformations, resulting in averaged phosphorus environments and a single NMR signal (Figure S32). Upon cooling, the exchange slows due to reduced thermal energy, leading to distinct chemical environments for the phosphine ligands (Figure S32). This manifests as an AA’BB’ spin system in the NMR spectrum, similar to what has been reported for the complex [Au­(XantPhos)_2_]­[Au­(C_6_Cl_2_F_3_)_2_], which features a halogenated phenyl group as the anionic ligand.^27^

Since compound **10** is a neutral species, while compound **11** is a salt composed of an anionic and a cationic complex, the equilibrium is expected to shift toward **11** in polar solvents, which better dissolve the anions. Conversely, nonpolar solvents may favor the formation of **10**. To test this hypothesis, crystals of **11** were dissolved in deuterated toluene, yielding **10** as the sole species in solution. To improve the synthesis of **10**, the reaction was repeated using toluene as the solvent, which enabled the isolation of the complex in pure form. Similarly, attempts to obtain pure **11** by performing the reaction in polar solvents such as acetone or acetonitrile resulted in a partial shift of the equilibrium toward **11**, making it the major product, though not exclusively.

One of the most notable examples of Click Chemistry is the synthesis of triazoles from azides and alkynes. In 1961, Rolf Huisgen reported the 1,3-dipolar cycloaddition of an alkyne with an azide under heating, yielding a mixture of 1,4- and 1,5-isomers.^28,29^ Later, Sharpless and Meldal demonstrated that copper­(I) salts not only accelerate the reaction but also exclusively produce the 1,4-isomer.^30,31^ In 2011, Adam S. Veige observed that gold­(I) alkynyl complexes activated alkynes similarly to copper in CuAAC, yielding bimetallic triazolates when reacting with gold azides.^32^ These metal-involved processes, termed iClick reactions, highlight the versatility of gold-mediated cycloadditions. Additionally, literature reports describe gold­(I)-coordinated azides reacting with alkynes to form monometallic triazoles through system rearrangement, where gold binds to a triazole carbon, and a proton shifts to a nitrogen. The iClick reaction with gold is similar to the copper-catalyzed azide–alkyne cycloaddition but differs due to gold­(I)’s soft Lewis acid nature. Gold­(I) coordinates to the alkyne, making it more electrophilic and prone to attack by the terminal nitrogen of the azide, forming a vinyl–gold intermediate. This undergoes intramolecular cyclization by the azide’s internal nitrogen, producing a triazolyl–gold compound. The reaction favors regioselectivity toward the 1,5-disubstituted triazole, unlike CuAAC which yields the 1,4-isomer.^33^

The synthesis and characterization of two gold­(I) triazoles have been successfully achieved through the reaction of the **EPC** precursor with gold azides, using PPh_3_ and JohnPhos as auxiliary ligands (Scheme 5). The reactions were conducted in acetonitrile/dichloromethane or toluene at room temperature (r.t.) under an argon atmosphere, with products isolated via diethyl ether precipitation. Without a copper catalyst, the reaction required 1 week to complete, but the addition of copper significantly accelerated the process.

**Scheme 5:**
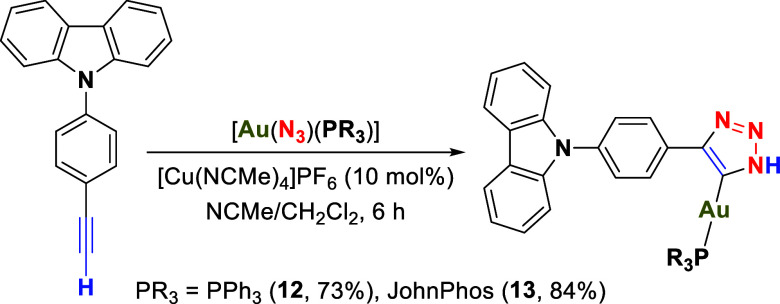
Synthesis of the Triazole-NHC-Gold­(I) Complexes **12** and **13**

The validation of the reaction can be performed using ^31^P­{^1^H} NMR experiments, where comparing the chemical shifts with those of the starting azides allows for the identification of a new and pure product. In the ^1^H NMR spectrum, the formation of the triazole has a significant effect on the ring that separates the alkyne from the carbazole in complex **12**, shifting the signal of its protons from 7.73 to 8.58 ppm (Figure S26), which is not observed for complex **13**.

### Crystal Structure Determination

Suitable crystals for X-ray diffraction studies of complex **1** were obtained through the slow diffusion of hexane into a dichloromethane solution of the complex. The compound crystallizes in the monoclinic system, within space group P2(1)/*n*, featuring one molecule per asymmetric unit (Figure 1).

**Figure 1:**
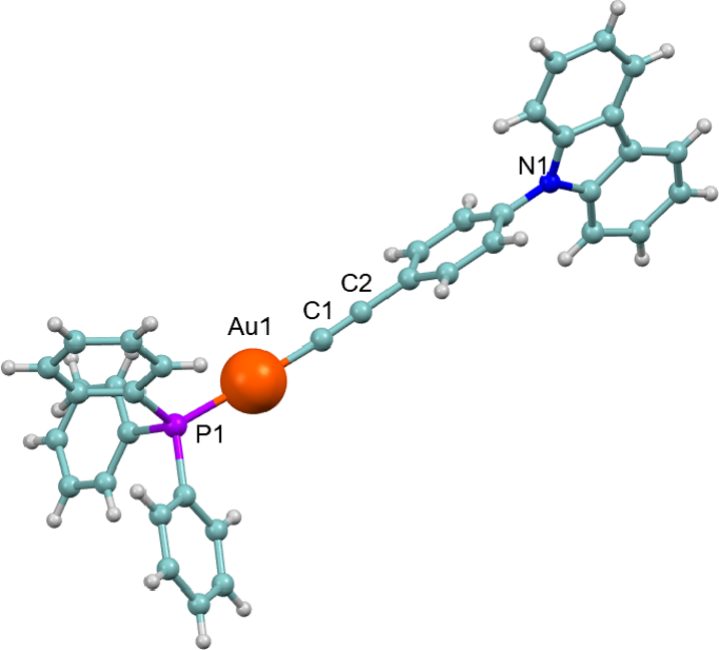
Molecular structure of complex **1**.

The Au–C bond distance of 1.9966(17) Å indicates a strong interaction, comparable to those found in other alkynyl gold complexes. Similarly, the Au–P bond distance of 2.2696(4) Å aligns with values reported for other alkynyl gold­(I) phosphine complexes.^26^ The gold atom adopts a linear coordination geometry, characteristic of gold­(I), with an almost ideal C1–Au1–P1 bond angle of 178.34(5)°.

The C1–C2–C3 chain exhibits a nearly linear arrangement, with a bond angle of 176.57(18)°, highlighting the overall structural linearity. Despite this, and the presence of planar molecular units, no significant short contacts are observed between the carbazole units. This is likely due to the nearly perpendicular orientation of the phenyl ring relative to the carbazole moiety, as well as the steric hindrance introduced by the bulky PPh_3_ ligand.

The crystal structure of complex **11** was determined by X-ray diffraction, revealing the ionic nature of the molecule, as shown in Figure 2. The structure consists of an anionic bis­(alkynyl)gold fragment and a cationic bis­(diphosphine)gold species. The Au–C bond distances in the alkynyl fragment are 1.983(7) and 1.997(6) Å, comparable to those observed in complex **1**. The tetra-coordinated gold­(I) species feature Au–P bond lengths ranging from 2.4451(12) to 2.4919(13) Å. The two gold centers exhibit distinct coordination geometries: one adopts a nearly linear arrangement with a Au–C–C angle of 176.7(2)°, while the other is tetra-coordinated, with diphosphine bite angles of approximately 110°. However, due to the limited quality of the crystal data, the reported bond distances and angles should be treated with caution.

**Figure 2:**
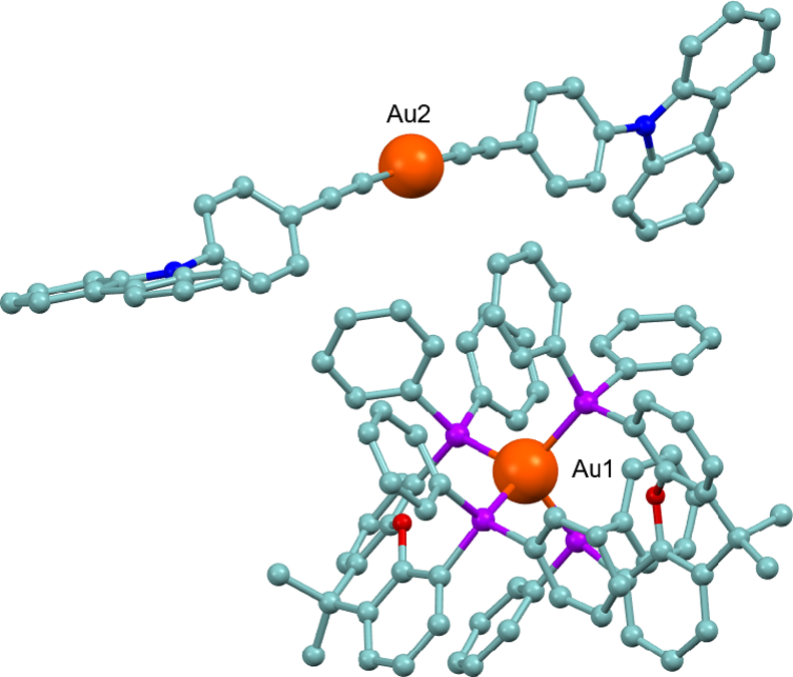
Molecular structure of complex **11**. Hydrogen atoms have been omitted for clarity.

The crystal structure of the triazole-carbene compounds **12** and **13** were determined by X-ray diffraction. Both complexes crystallized in the triclinic system with space group P(−1), featuring three independent molecules, one of which is depicted in Figure 3 for **12** and **13**. The data confirms that the gold azide species has been added to the triple bond, remaining coordinated to the carbon atom and generating an N-heterocyclic carbene derivative. The Au–C bond distances of 2.032(4) Å (**12**) or 2.027(4) (**13**), in one of the molecules, are comparable to other NHC–Au derivatives but slightly longer than those observed in the alkynyl-gold species discussed here. The Au–P distance of 2.3026(9) Å likely reflects the stronger *trans* influence of the NHC compared to the alkynyl moiety. Additionally, the C–Au–P bond angle of 176.51(10)° confirms the linearity of the gold center.

**Figure 3:**
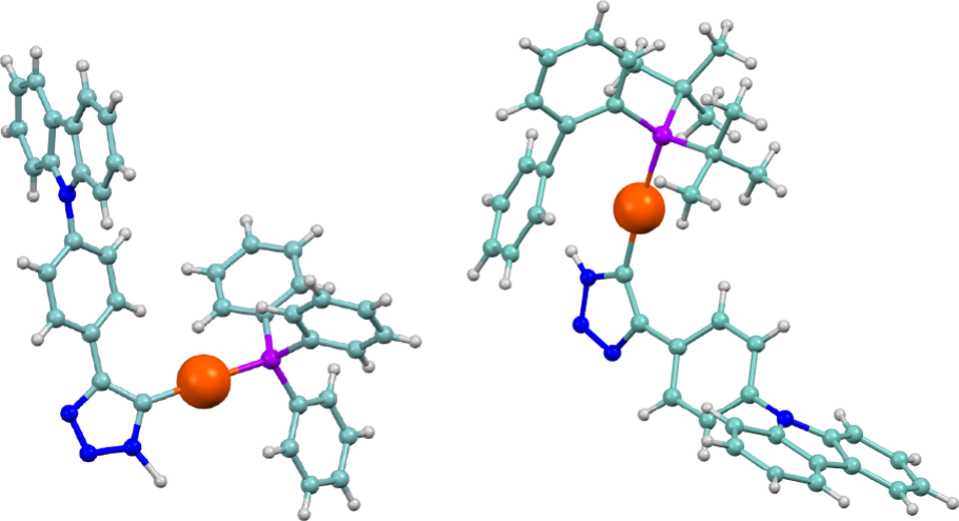
Molecular structure of one of the independent molecules of complexes **12** or **13**.

The presence of the triazole moiety facilitates the formation of N–H···N hydrogen bonds in both complexes. Figure 4 shows those for complex **12** with N–H···N hydrogen bonds of 1.565 Å and N_donor_···N_aceptor_ of 2.892 Å with nearly ideal linearity. These molecules form a continuous chain that extends into a supramolecular network (Figure 4). Additionally, a Au···H interaction with the proton of the phenyl ring is observed in both complexes, which could be responsible for the downfield shift of this proton in the ^1^H NMR spectrum of the complex.

**Figure 4:**
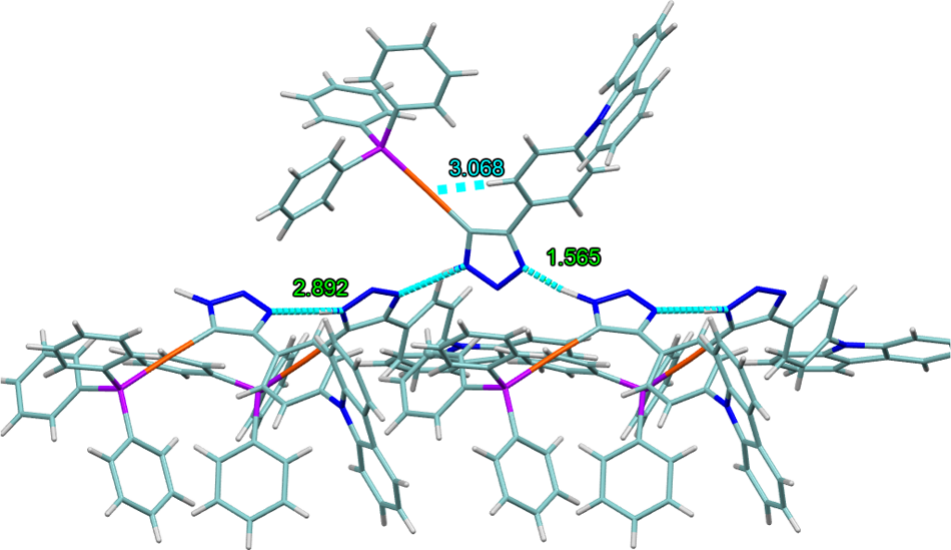
Association of molecules of **12** through hydrogen bonding.

### Photophysical Properties

The electronic absorption spectra of the ligand precursor in solution exhibit intense high-energy absorptions between 240 and 280 nm, along with a lower-energy absorption band in the range of approximately 295–350 nm. The high-energy absorptions are attributed to intraligand (IL) π–π* transitions (originating from the carbazole and alkynyl units), while the lower-energy absorptions may be ascribed to intraligand n−π* transitions in the carbazole mixed with intramolecular charge transfer (ICT) transitions (Figure S34).^34,35^ The gold complexes exhibit similar absorptions within the same wavelength range, indicating a common origin (see ESI for all absorption spectra). However, only complex containing the imidazole moiety shows a stronger absorption for the second band, possibly due to the involvement of IL/ICT in the nitrogen ligand (Figure S50).

Excitation and emission spectra for the ligand and complexes **1–10, 12** and **13** were measured at room temperature and at 77 K. The starting ligand **EPC** exhibits an excitation maximum at 352 nm, which shifts to 337 nm when the solid is cooled in liquid nitrogen. Its emission spectrum features a narrow, high-intensity band with a maximum at 449 nm, which shows minimal shift with temperature (446 nm). Additionally, a second, broader, and less intense band is observed with a maximum at 467 nm (Figure 5).

**Figure 5:**
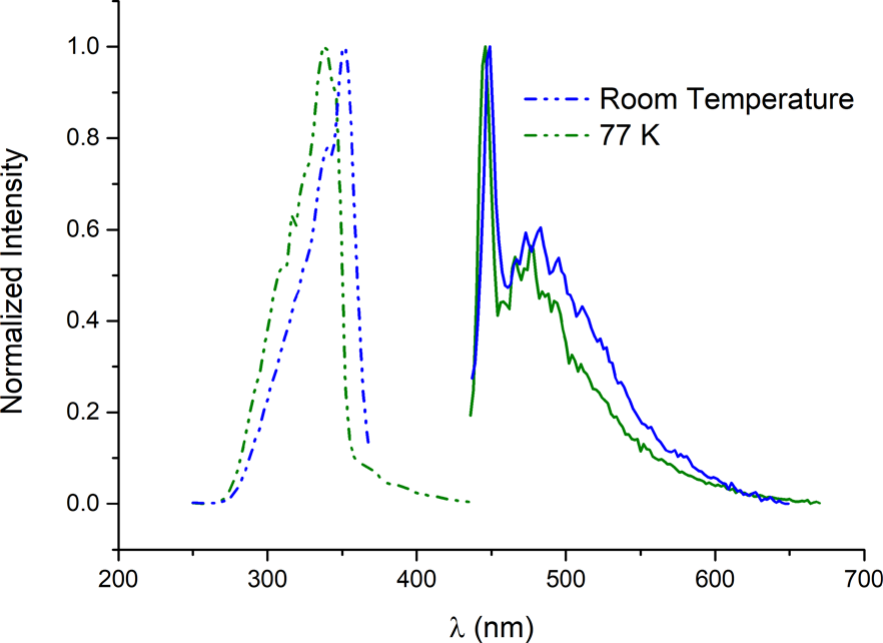
Normalized excitation and emission bands for the **EPC** precursor at r.t. and 77 K.

All complexes (**1–10, 12, 13**) exhibited strong blue-green luminescence in the solid state, particularly at 77 K (see ESI for all excitation and emission spectra). At room temperature, they displayed a broad emission band in the 450–600 nm range, which is both stronger and red-shifted compared to the ligand. This emission likely originates from a combination of ^3^IL excited states mainly based on the carbazole. These transitions are facilitated by the phenyl ring and are influenced by metal coordination. However, a minor contribution from a Au–L charge transfer transition to the alkynyl orbital, specifically a ^3^[σ­(Au–L)→π­(C≡C)] transition, may also be present, as observed in some gold­(I) alkynyl complexes.^26^

Figure 6 presents the excitation and emission bands for complex **1** at both room temperature and 77 K, serving as a representative example for the rest of the complexes (see ESI).

**Figure 6:**
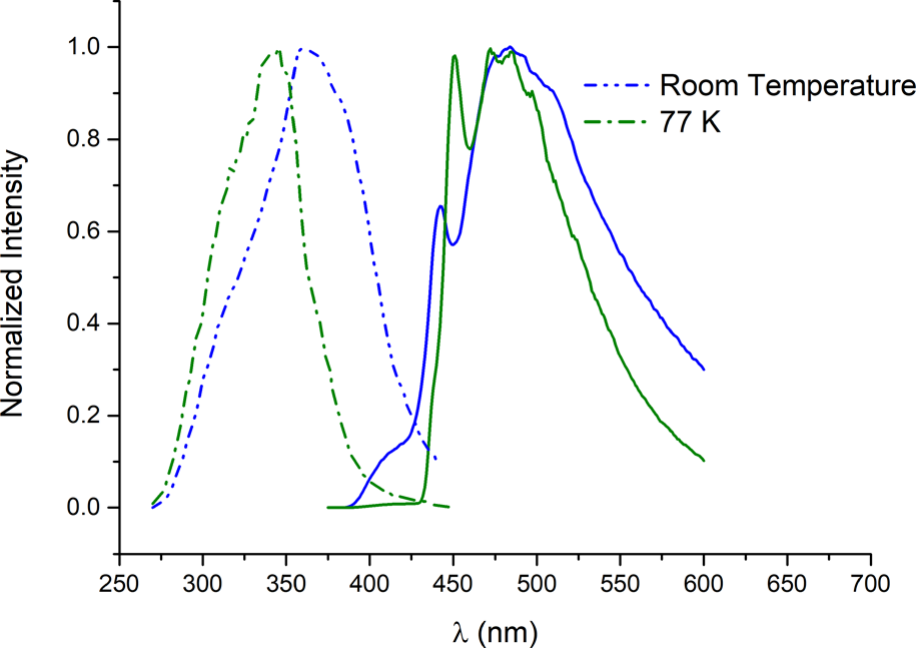
Normalized excitation and emission bands for complex **1** at r.t. and 77 K.

Complexes **12** and **13**, which are triazole-NHC derivatives, exhibit distinct dual emission at room temperature (Figure 7 for complex **13**). The most energetic emission is likely attributed to fluorescence from an intraligand transition involving the triazole moiety, while the second emission appears as a structured band, similar to those observed in previous complexes. At 77 K, the spectra reveal a structured emission akin to that of the earlier complexes, originating from a ^3^IL transition in the ligands.

**Figure 7:**
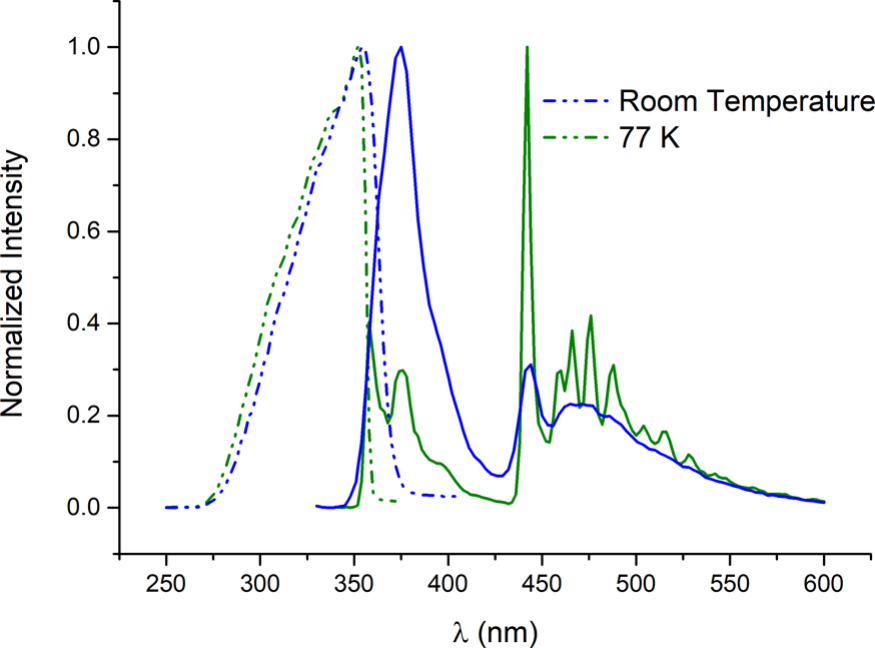
Normalized excitation and emission bands for complex **13** at r.t. and 77 K.

Lifetimes fall within the microsecond range, consistent with phosphorescent emission. However, the broad emission bands suggest the possible presence of two emissive states (biexponential decay). As a result, a minor contribution from intraligand fluorescence cannot be fully ruled out based on lifetime measurements alone.

The quantum yield of some of the complexes has been measured in the solid state, revealing a moderate value of 8% for complexes **3** and **9**. The highest quantum yield was observed for the triazolyl complex **13** bearing a JohnPhos ligand, reaching a value of 12%. Interestingly, the analogous complex **12**, which features PPh_3_ as the auxiliary ligand, shows a significantly lower quantum yield. This observation aligns with the findings of Gray and co-workers, who reported that variations in both the auxiliary ligands and the linkage modes to the gold centers markedly influence the excited-state dynamics in a series of dinuclear gold complexes.^36^ Table 1 summarizes all the photophysical properties of these complexes.

**Table 1:** Excitation, Emission, Lifetimes and Quantum Yield of the Ligand Precursor and the Gold Complexes

complex	*T* (K)	λ_ex_ (nm)	λ_em_ (nm)	τ(μs)	ϕ
**L**	r.t.	352	449, 467		
	77	337	446, 467		
**1**	r.t.	360	442, 483	34	0.03
	77	348	454, 472	395	
**2**	r.t.	396	526	326	
	77	348	538	74,000	
**3**	r.t.	358	444, 487	522	0.08
	77	352	442, 476	1141	
**4**	r.t.	334	439, 470	171	
	77	352	434, 469	232	
**5**	r.t.	314	454. 511	145	0.01
	77	320	448. 186	224	
**6**	r.t.	354	449, 484	24	0.01
	77	348	450, 482	322	
**7**	r.t.	348	439, 470	53	
	77	350	435, 467	164	
**8**	r.t.	355	432, 463	51	
	77	350	435, 457	416	
**9**	r.t.	352	492	14	0.08
	77	387	451, 476	168	
**10**	r.t.	368	513	15	
	77	356	458, 502	158	
**12**	r.t.	350	374, 442, 470	110.9	<0.01
	77	352	437, 469	434.9	
**13**	r.t.	354	375, 444, 470	56.3	0.12
	77	352	358, 376, 442, 469	234.1	

Due to the significant increase in the emission lifetime of complex **2** upon cooling to 77 K, the presence of thermally activated delayed fluorescence (TADF) was initially considered. To investigate this possibility, temperature-dependent lifetime measurements were conducted over the 80–300 K range, and the data were fitted using a model based on the Boltzmann distribution (see ESI). A Δ*E*(S_1_–T_1_) value of 3758 cm^–1^ was determined, substantially higher than typical values reported for gold complexes. This large energy gap makes efficient TADF unlikely. Instead, the observed long lifetimes are more plausibly attributed to phosphorescence from the triplet state, which is stabilized at low temperatures. The multiexponential decay behavior may arise from multiple emissive states or heterogeneous microenvironments, such as different conformers, packing arrangements, or aggregation effects, particularly in the solid state.^37,38^

### Theoretical Calculations

TD-DFT calculations employing the Tamm-Dancoff approximation were performed to analyze the molecular orbitals involved in both the absorption (S0 → S_1_) and emission (T_1_ → S0) transitions of the complex **1**. Figure 8 illustrates the molecular orbitals with the highest oscillator strengths contributing to these transitions (see ESI). The results support our initial hypothesis, indicating that the emission is predominantly of intraligand character, although coordination to the Au­(I) center induces slight modifications in the energy levels and transition intensities.

**Figure 8:**
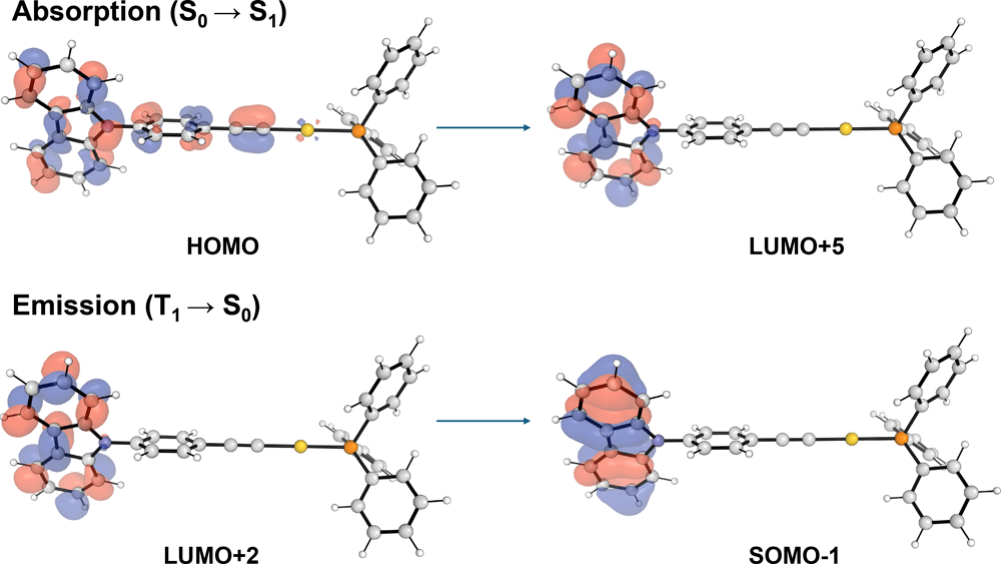
Molecular Orbitals involved in the absorption and emission of complex **1**.

## Conclusions

This study successfully highlights the versatility of gold­(I) alkynyl complexes in molecular design. The combination of the linear coordination geometry of the gold center and the functional tunability of alkynyl ligands enables the construction of diverse molecular architectures, including mononuclear, dinuclear, three-coordinated, and supramolecular structures. A series of Au­(I) alkynyl complexes were synthesized using multiple strategies, including direct alkynylation and polymer-based precursors. Notably, a three-coordinated complex exhibited a solvent-dependent equilibrium between neutral and ionic gold species, offering a strategy for controlled synthesis. The incorporation of triazole moieties via iClick reactions further expands the synthetic scope of gold­(I) alkynyl chemistry. Additionally, the formation of triazol-NHC-gold­(I) complexes provides new insights into ligand design and the potential for fine-tuning their properties.

The crystal structures reveal that, despite the presence of planar molecular units, π-π interactions are absent, likely due to the nearly perpendicular orientation of the carbazole unit relative to the phenyl ring. However, the triazole derivatives exhibit strong hydrogen bonding through NH···N interactions, leading to the formation of extended supramolecular assemblies.

Photophysical studies confirm that gold coordination enhances the luminescent properties of carbazole-alkynyl ligands. The observed redshift and increased emission intensity in the gold complexes, compared to the free ligand, suggest that, in addition to intraligand transitions within the carbazole, and intramolecular charge transfer (ICT) transitions are present. TD-DFT calculations were performed to analyze the molecular orbitals involved in both the absorption (S0 → S_1_) and emission (T_1_ → S0) transitions of the complex **1**, indicating that the emission is predominantly of intraligand (^3^IL) character.

## Experimental Section

### Instrumentation

NMR spectra were recorded on Bruker ARX300, AV300 or AV400 spectrometers. ^1^H NMR spectra were recorded at 300 or 400 MHz; ^13^C­{^1^H}-APT NMR spectra were recorded at 75 or 101 MHz; ^31^P­{^1^H} NMR spectra were recorded at 121 MHz or162 MHz. Chemical shifts are described on the scale (δ ppm) relative to the residual peaks of CHCl_3_ (7.28 ppm), toluene (2.09 ppm) and CH_2_Cl_2_ (5.32 ppm) for ^1^H NMR and to the central line of CDCl_3_ (77 ppm), toluene-*d*_8_ (20.40 ppm) and CD_2_Cl_2_ (53.84 ppm) for ^13^C­{^1^H}-APT NMR. A Bruker MicroToF-Q spectrometer was used for High-resolution mass spectra-ESI (HRMS-ESI) equipped with an API-ESI source and a QToF mass analyzer, both allowing a maximum error in the measurement of 5 ppm. Steady-state photoluminescence spectra and lifetime measurements were recorded with a FluoTime300 PicoQuant spectrometer as powder samples, placed in a quartz tube. A liquid nitrogen dewar assembly was used for the studies at 77 K. Photoluminescence measurements were performed using the excitation wavelength corresponding to the maximum in the excitation spectrum, which aligns with the wavelength that yields the highest emission intensity. Conversely, the emission spectra were collected using the excitation wavelength that produces maximum emission. The excitation and emission slit widths were set to 2 and 4 nm, respectively. A double-pass monochromator was used for both excitation and emission paths. No band-pass filters were employed during the measurements.

Quantum yields were measured by the absolute method using a Hamamatsu Quantaurus-QY C11347 compact one-box absolute quantum yield measurement system. In order to prove the reproducibility of the measurements, three or more measurements were carried out for each compound with different amount of solid powder sample. Through studies carried out for different substances, using both the absolute method and the comparative one, the relative uncertainty for the absolute method has been determined as less than 6%.^39^

### Synthesis and Characterization of the Complexes

#### Synthesis of [AuCl­(L)] (L = PR_3_, CNCy) Precursors

To a solution of [AuCl­(tht)] (320 mg, 1 mmol) in dichloromethane (10 mL) is added 1 equiv of the chosen phosphine. The mixture is stirred for 30 min and then, hexane (15 mL) is added to obtain the desired product as a white precipitate, which is filtered and washed with more hexane. After drying them under vacuum, the products are obtained in quantitative yield (>95%) and used without further purification. The [AuCl­(IPr)] compound was prepared using the previous methodology.^40^

#### Synthesis of the Polymer [AuC≡CR]*_n_* (C≡CR = 9-(4-Ethynylphenyl)-9H-carbazole)

9-(4-Ethynylphenyl)-9*H*-carbazole (133 mg, 0.5 mmol) is added to a solution of [AuCl­(tht)] (160 mg, 0.5 mmol) in CH_2_Cl_2_ (20 mL). The resulting yellow solution is stirred for 5 min, after which triethylamine (0.1 mL) is added, leading to the formation of a yellow precipitate. The mixture is then stirred for an additional hour. The desired product is obtained in a 49% yield (108 mg) by filtration, followed by successive washes with methanol (3 × 5 mL) and diethyl ether (3 × 5 mL).

#### Synthesis of [Au­(N_3_)­(PR_3_)] Derivatives

These compounds were prepared differently than the published procedure.^41^ To a solution of [AuCl­(PR_3_)] (1 mmol) in toluene (10 mL), AgOAc (167 mg, 1 mmol) is added, and the resulting solution is stirred, protected from sunlight, for 12 h at room temperature. After this reaction time, the solution is passed through a Celite filter, which is then washed with an excess of toluene (3 × 5 mL). TMSN_3_ (1 mmol) is then added to the resulting solution and stirred for another 12 h at room temperature. After the reaction time, the volume is reduced by half under vacuum, and the desired product is precipitated with cold hexane, isolated by filtration and dried under vacuum.

##### Synthesis of Alkynyl Gold Complexes (**1–6** and **9**)

For complexes **1–6** and **9**: To a solution of KOH (0.3 mmol, 1.5 equiv) in MeOH (10 mL), the alkyne 9-(4-ethynylphenyl)-9*H*-carbazole (0.2 mmol, 1 equiv), and [AuCl­(L)] (0.2 mmol, 1 equiv) were added. The mixture was stirred overnight at room temperature, and the resulting precipitate was isolated by filtration. The precipitate was then washed with cold methanol (3 × 5 mL) and diethyl ether (3 × 5 mL), and dried under vacuum. For complex **9**, 2 equiv of the ligand 9-(4-ethynylphenyl)-9*H*-carbazole are used instead of 1, and 3 equiv of KOH instead of 1.5.

##### Complex **1**

Following the general procedure, complex **1** was obtained after 14 h of reaction, as a pale white solid (116 mg, 80% yield). ^1^H NMR (400 MHz, CDCl_3_) δ 8.13 (d, *J* = 7.8 Hz, 2H, H5), 7.73 (d, *J* = 8.5 Hz, 2H, H8), 7.62–7.37 (m, 21H, PPh_3_, H3, H2, H9), 7.30–7.25 (m, 2H, H4). ^13^C­{^1^H}-APT NMR (101 MHz, CDCl_3_) δ 140.9 (s, C1), 136.2 (s, C7), 134.5 (d, *J* = 13.8 Hz, Ph), 133.9 (s, C8), 133.5 (d, *J* = 145.4 Hz, P–C), 131.7 (d, *J* = 2.4 Hz, Ph), 129.8 (d, *J* = 54.5 Hz, C12), 129.3 (d, *J* = 11.3 Hz, Ph), 126.7 (s, C9), 126.1 (s, C3), 124.2 (d, *J* = 3.0 Hz, C10), 123.5 (s, C6), 120.3 (s, C5), 120.0 (s, C4), 110.0 (s, C2), 103.5 (d, *J* = 26.3 Hz, C11). ^31^P­{^1^H} NMR (162 MHz, CDCl_3_) δ 42.4 (s, 1P, PPh_3_). HRMS (ESI-QTOF) *m*/*z*: [M + Na]^+^ Calculated for C_38_H_27_AuNNaP 748.1439; found 748.1439.

##### Complex **2**

Following the general procedure, complex **2** was obtained after 12 h of reaction, as a pale-yellow solid (137 mg, 94% yield). ^1^H NMR (400 MHz, CDCl_3_) δ 8.82 (d, *J* = 4.3 Hz, 1H, Ha), 8.16 (d, *J* = 7.7 Hz, 2H, H5), 8.06 (t, *J* = 7.6 Hz, 1H, Hd), 7.84–7.74 (m, 7H, Hc, H8, Ph), 7.57–7.37 (m, 13H, Hb, H3, H2, H9, Ph), 7.33–7.28 (m, 2H, H4). ^13^C­{^1^H}-APT NMR (101 MHz, CDCl_3_) δ 155.3 (d, *J* = 77.1 Hz, Ce), 151.5 (d, *J* = 14.5 Hz, Ca), 140.8 (s, C1), 136.6 (d, *J* = 11.0 Hz, *Cc*), 136.2 (s, C7), 134.8 (d, *J* = 13.8 Hz, Ph), 133.9 (s, C8), 131.9 (d, *J* = 33.1 Hz, Cd), 131.8 (d, *J* = 2.3 Hz, Ph), 129.5 (d, *J* = 57.0 Hz, C12), 129.1 (d, *J* = 11.5 Hz, Ph), 126.7 (s, C9), 126.1 (s, C3), 125.3 (d, *J* = 2.2 Hz, Cb), 124.2 (s, C10), 123.5 (s, C6), 120.4 (s, C5), 120.1 (s, C4), 110.0 (s, C2), 103.5 (s, C11). ^31^P­{^1^H} NMR (121 MHz, CDCl_3_) δ 41.2 (s, 1P, PPh_2_Py). HRMS (ESI-QTOF) *m*/*z*: [M + Na]^+^ Calculated for C_37_H_26_AuN_2_NaP 749.1391; found 749.1391.

##### Complex **3**

Following the general procedure, complex **3** was obtained after 12 h of reaction, as a pale-yellow solid (143 mg, 93% yield). ^1^H NMR (400 MHz, CDCl_3_) δ 8.14 (d, *J* = 7.7 Hz, 2H, H5), 7.89 (td, *J* = 7.1, 1.5 Hz, 1H, JohnPhos), 7.71–7.65 (m, 2H, H8), 7.65–7.38 (m, 11H, JohnPhos, H2, H3, H9), 7.34–7.16 (m, 5H, JohnPhos, H4), 1.45 (d, *J* = 15.0 Hz, 18H, C*H*_3_). ^13^C­{^1^H}-APT NMR (101 MHz, CDCl_3_) δ 150.4 (d, *J* = 15.0 Hz, JohnPhos), 142.6 (d, *J* = 6.1 Hz, JohnPhos), 141.0 (s, C1), 135.9 (d, *J* = 131.5 Hz, Ca), 135.4 (s, C7), 134.5 (d, *J* = 1.2 Hz, JohnPhos), 133.6 (s, C8), 133.2 (d, *J* = 7.3 Hz, JohnPhos), 130.4 (d, *J* = 2.1 Hz, JohnPhos), 129.4 (s, JohnPhos), 129.2 (s, JohnPhos), 128.2 (s, JohnPhos), 127.6 (d, *J* = 39.9 Hz, C12), 126.8 (d, *J* = 5.9 Hz, JohnPhos), 126.5 (s, C9), 126.0 (s, C3), 125.6 (d, *J* = 2.4 Hz, C10), 123.5 (s, C6), 120.3 (s, C5), 119.9 (s, C4), 110.1 (s, C2), 101.6 (d, *J* = 23.2 Hz, C11), 37.7 (d, *J* = 22.4 Hz, *C*Me_3_), 31.2 (d, *J* = 7.0 Hz, CH_3_). ^31^P­{^1^H} NMR (162 MHz, CDCl_3_) δ 64.5 (s, 1P, JohnPhos). HRMS (ESI-QTOF) *m*/*z*: [M + Na]^+^ Calculated for C_40_H_39_AuNNaP 784.2378; found 784.2378.

##### Complex **4**

Following the general procedure, complex **4** was obtained after 12 h of reaction, as a white solid (160 mg, 98% yield). ^1^H NMR (300 MHz, CDCl_3_) δ 8.14 (d, *J* = 7.7 Hz, 2H, H5), 7.88–7.78 (m, 1H, JohnPhos), 7.75–7.66 (m, 2H, H8), 7.61–7.36 (m, 11H, JohnPhos, H2, H3, H9), 7.35–7.16 (m, 5H, JohnPhos, H4), 2.22–2.06 (m, 2H, Hk), 2.05–1.92 (m, 2H, H^Cy^), 1.88–1.50 (m, 10H, H^Cy^), 1.46–1.11 (m, 8H, H^Cy^). ^13^C­{^1^H}-APT NMR (101 MHz, CDCl_3_) δ 149.0 (d, *J* = 10.1 Hz, JohnPhos), 141.8 (d, *J* = 5.0 Hz, JohnPhos), 140.9 (s, C1), 136.9 (d, *J* = 135.3 Hz, Ca), 135.6 (s, C7), 135.2 (d, *J* = 9.1 Hz, JohnPhos), 133.7 (s, C8), 132.4 (d, *J* = 7.1 Hz, JohnPhos), 130.6 (d, *J* = 2.0 Hz, JohnPhos), 129.3 (s, JohnPhos), 128.9 (s, JohnPhos), 128.3 (s, JohnPhos), 127.5 (d, *J* = 9.1 Hz, JohnPhos), 126.5 (s, C9), 126.4 (d, *J* = 46.5 Hz, C12), 126.0 (s, C3), 125.1 (d, *J* = 2.0 Hz, C10), 123.4 (s, C6), 120.3 (s, C5), 119.9 (s, C4), 110.0 (s, C2), 102.0 (s, d, *J* = 25.3 Hz, C11), 36.6 (d, *J* = 30.1 Hz, *C*H^Cy^), 31.2 (d, *J* = 5.3 Hz, *C*H_2_^Cy^), 29.6 (s, *C*H_2_^Cy^), 26.8 (d, *J* = 4.4 Hz, *C*H_2Cy_), 26.7 (d, *J* = 6.6 Hz, *C*H_2Cy_), 25.7 (s, *C*H_2_^Cy^). ^31^P­{^1^H} NMR (121 MHz, CDCl_3_) δ 50.4 (s, 1P, ^Cy^JohnPhos). HRMS (ESI-QTOF) *m*/*z*: [M + Na]^+^ Calculated for C_44_H_43_AuNNaP 836.2691; found 836.2691.

##### Complex **5**

Following the general procedure, compound **5** was obtained after 12 h of reaction, as a white solid (93 mg, 82% yield). ^1^H NMR (400 MHz, CDCl_3_) δ 8.13 (d, *J* = 7.7 Hz, 2H, H5), 7.68 (d, *J* = 8.4 Hz, 2H, H8), 7.45 (d, *J* = 8.5 Hz, 2H, H9), 7.43–7.36 (m, 4H, H2, H3), 7.32–7.23 (m, 2H, H4), 3.88 (tt, *J* = 8.1, 3.8 Hz, 1H, C*H*-NC), 2.07–1.95 (m, 2H, H^Cy^), 1.88–1.69 (m, 4H, H^Cy^), 1.57–1.40 (m, 4H, H^Cy^). ^13^C­{^1^H}-APT NMR (101 MHz, CDCl_3_) δ 140.9 (s, C1), 136.3 (s, C7), 134.0 (s, C8), 126.7 (s, C9), 126.1 (s, C3), 124.0 (s, C10), 123.5 (s, C6), 123.4 (s, C12), 120.4 (s, C5), 120.1 (s, C4), 110.0 (s, C2), 102.7 (s, C11), 54.8 (s, Ca), 31.7 (s, C^Cy^), 24.7 (s, C^Cy^), 22.7 (s, C^Cy^). HRMS (ESI-QTOF) *m*/*z*: [M + H]^+^ Calculated for C_27_H_24_AuN_2_ 573.1600; found 573.1573.

##### Complex **6**

Following the general procedure, complex **6** was obtained after 12 h of reaction, as a white solid (121 mg, 71% yield). ^1^H NMR (300 MHz, CDCl_3_) δ 8.03 (d, *J* = 7.7 Hz, 2H, H5), 7.51–7.42 (m, 4H, H8, Ha), 7.33–7.13 (m, 12H, H9+H4+H3+H2+Hb), 7.07 (s, 2H, He), 2.57 (hept, *J* = 6.8 Hz, 4H, Hf), 1.35 (d, *J* = 6.8 Hz, 12H), 1.17 (d, *J* = 6.9 Hz, 12H). ^13^C­{^1^H}-APT NMR (101 MHz, CDCl_3_) δ 191.0 (s, Ch), 145.8 (s, Cd), 140.9 (s, C1), 135.1 (s, C7), 134.4 (s, *Cc*), 133.7 (s, C8), 131.2 (s, C12), 130.6 (s, Ca), 126.4 (s, C9), 125.9 (s, C3), 125.5 (s, C10), 124.3 (s, Cb), 123.4 (s, Ce), 123.4 (s, C6), 120.3 (s, C5), 119.8 (s, C4), 110.0 (s, C2), 103.1 (s, C11), 29.0 (s, Cf), 24.8 (s, Cg), 24.2 (s, Cg). HRMS (ESI-QTOF) *m*/*z*: [M + H]^+^ Calculated for C_47_H_49_AuN_3_ 852.3586; found 852.3587.

##### Complex **7**

The polymer [AuC≡CR]_*n*_ (66 mg, 0.15 mmol) is added to a solution of *N,N*-dimethylpyridin-4-amine (18 mg, 0.15 mmol) in CH_2_Cl_2_ (5 mL), and the resulting solution is stirred for 2 h. After the reaction time, the reaction volume is reduced to 1 mL, and hexane (10 mL) is added to obtain the product as a white solid (78 mg, 92%), which is then filtered and dried under vacuum. ^1^H NMR (400 MHz, CD_2_Cl_2_) δ 8.14 (d, *J* = 7.7 Hz, 2H, H5), 8.05 (d, *J* = 6.5 Hz, 2H, Ha), 7.60 (d, *J* = 8.2 Hz, 2H, H8), 7.50–7.36 (m, 6H, H2, H3, H9), 7.32–7.24 (m, 2H, H4), 6.59 (d, *J* = 6.5 Hz, 2H, Hb), 3.08 (s, 6H, Hd). ^13^C­{^1^H}-APT NMR (101 MHz, CDCl_3_) δ 150.4 (s, Ca), 141.0 (s, C1), 134.0 (s C8), 126.6 (s, C9), 126.0 (s, C3), 123.5 (s, C6), 120.3 (s, C5), 120.0 (s, C4), 110.1 (s, C2), 107.6 (s, Cb), 39.5 (s, Cd). HRMS (ESI-QTOF) *m*/*z*: [M + Na]^+^ Calculated for C_27_H_22_AuN_3_Na 608.1372; found 608.1388.

##### Complex **8**

The polymer [AuC≡CR]_*n*_ (49 mg, 0.11 mmol) is added to a solution of 1-methylimidazole (9 mg, 0.11 mmol) in CH_2_Cl_2_ (5 mL), and the resulting solution is stirred for 2 h. After the reaction time, the reaction volume is reduced to 1 mL, and hexane (10 mL) is added to obtain the product as a white solid (36 mg, 61%), which is then filtered and dried under vacuum. ^1^H NMR (300 MHz, CDCl_3_) δ 8.13 (d, *J* = 7.7 Hz, 2H, H5), 7.75–7.64 (m, 3H, H8, Hc), 7.51–7.36 (m, 6H, H9, H3, H2), 7.33–7.26 (m, 2H, H4), 7.13 (s, 1H, H^Im^), 7.08 (s, 1H, H^Im^), 3.82 (s, 3H, Hd). The solubility of compound **8** is too low to determine its ^13^C­{^1^H}-APT NMR spectrum. HRMS (ESI-QTOF) *m*/*z*: [M + Na]^+^ Calculated for C_24_H_18_AuN_3_Na 568.1058; found 568.1038.

##### Complex **9**

For this complex, 2 equiv of 9-(4-ethynylphenyl)-9*H*-carbazole are added to the solution instead of one. Following the general procedure, complex **9** was obtained after 12 h of reaction, as a pale white solid (209 mg, 78% yield). ^1^H NMR (400 MHz, CDCl_3_) δ 8.13 (d, *J* = 7.7 Hz, 4H, H5), 7.78–7.60 (m, 12H, H8, Ph), 7.54–7.34 (m, 24H, H9, H3, H2, Ph), 7.32–7.26 (m, 4H, H4), 2.52–2.34 (m, 4H, Ha), 1.74–1.62 (m, 2H, Hb). ^13^C­{^1^H}-APT NMR (101 MHz, CDCl_3_) δ 140.8 (s, C1), 136.2 (s, C7), 133.8 (s, C8), 133.5 (d, *J* = 13.5 Hz, Ph), 131.5 (s, Ph), 129.3 (d, *J* = 10.5 Hz, Ph), 126.7 (s, C9), 126.0 (s, C3), 124.2 (s, C10), 123.5 (s, C6, C12), 120.4 (s, C5), 120.1 (s, C4), 110.0 (s, C2), 103.5 (s, C11), 27.8 (d, *J* = 31.1 Hz, Ca), 24.9 (d, *J* = 2.9 Hz, Cb). ^31^P­{^1^H} NMR (162 MHz, CDCl_3_) 35.7 (s, 2P, PPh_2_CH_2_). HRMS (ESI-QTOF) *m*/*z*: [M + Na]^+^ Calculated for C_67_H_50_Au_2_N_2_NaP_2_ 1361.2672; found 1361.2673.

##### Complex **10**

The polymer [AuC≡CR]_*n*_ (49 mg, 0.10 mmol) is added to a solution of xanthphos (58 mg, 0.10 mmol) in toluene (5 mL), and the resulting solution is stirred for 2 h. After the reaction time, the reaction volume is reduced to 1 mL, and hexane (10 mL) is added to obtain the product **10** as a white solid (93 mg, 61%), which is then filtered and dried under vacuum. ^1^H NMR (300 MHz, toluene-*d*_8_) δ 8.01 (d, *J* = 7.6 Hz, 2H, H5), 7.70 (d, *J* = 8.5 Hz, 2H, H8), 7.54–7.36 (m, 8H, Ph), 7.35–7.14 (m, 8H, H4, H3, H2, Ph), 7.08–7.02 (m, 4H, H9, xanthphos), 6.96–6.86 (m, 10H, Ph), 6.77–6.65 (m, 4H, xanthphos), 1.33 (s, 6H, Me). ^13^C­{^1^H}-APT NMR (101 MHz, toluene*-d*_8_) δ 153.4 (app. t, *J* = 7.1 Hz, xanthphos), 141.5 (s, C1), 135.3 (s, C7), 134.7 (d, *J* = 10.1 Hz, xanthphos), 134.5 (d, *J* = 17.2 Hz, xanthphos), 134.5 (s, xanthphos), 133.7 (s, C8), 132.9 (s, xanthphos), 131.1 (s, xanthphos), 129.6 (s, xanthphos), 127.3 (s, xanthphos), 126.8 (s, C9), 126.1 (s, C3), 124.4 (s, xanthphos), 124.0 (s, C6), 122.2 (app. t, *J* = 8.1 Hz, xanthphos), 120.4 (s, C5), 120.0 (s, C4), 110.4 (s, C2), 104.1 (s, C11), 34.7 (s, *C*(CH_3_)), 30.8 (s, Me). ^31^P­{^1^H} NMR (162 MHz, toluene*-d*_8_) δ 4.5 (s, 2P, xanthphos). HRMS (ESI-QTOF) *m*/*z*: [M + Na]^+^ Calculated for C_59_H_44_AuNNaOP_2_ 1064.2461; found 1064.2456.

##### Complex **12**

**Method A:** 9-(4-ethynylphenyl)-9*H*-carbazole (25 mg, 0.1 mmol) is added to a solution of [Au­(N_3_)­(PPh_3_)] (47 mg, 0.1 mmol) in 5 mL of degassed toluene and under argon atmosphere. The resulting solution is stirred for 1 week at room temperature. After this reaction time, the volume of toluene is reduced to approximately 1 mL, and 10 mL of hexane is then added. The resulting precipitate is filtered, dried under vacuum and final complex **12** is obtained as a white solid (57 mg, 80%).

**Method B:** 9-(4-ethynylphenyl)-9*H*-carbazole (25 mg, 0.1 mmol) is added to a solution of [Au­(N_3_)­(PPh_3_)] (47 mg, 0.1 mmol) in a mixture of 6 mL of CH_2_Cl_2_ and 4 mL of acetonitrile, which has been previously purged with an argon stream for 10 min. [Cu­(NCMe)_4_]­PF_6_ (3 mg, 0.01 mmol) is then added. The resulting solution is stirred under argon atmosphere, at room temperature for 6 h. After this reaction time, the reaction mixture is filtered through silica and dried under vacuum. The obtained oil is dissolved in 1 mL of CH_2_Cl_2_, and complex **12** is finally isolated by precipitation with hexane, followed by filtration and dried under vacuum. Complex **12** is obtained as a white solid (52 mg, 73%).

^1^H NMR (400 MHz, CD_2_Cl_2_) δ 11.96 (br s, 1H, NH), 8.58 (d, *J* = 8.4 Hz, 2H, H8), 8.16 (d, *J* = 7.8 Hz, 2H, H5), 7.72–7.58 (m, 6H, Ph), 7.57–7.37 (m, 15H, Ph, H9, H3, H2), 7.36–7.23 (m, 2H, H4). ^13^C­{^1^H}-APT NMR (101 MHz, CD_2_Cl_2_) δ 152.9 (s, Cq), 141.4 (s, C1), 136.3 (s, C7), 134.7 (d, *J* = 13.8 Hz, Ph), 134.7 (s, (s, Cq), 132.2 (d, *J* = 2.1 Hz, Ph), 130.4 (s, Cq), 129.7 (d, *J* = 11.2 Hz, Ph), 128.0 (s, C8), 127.3 (s, C9), 126.3 (s, C3), 123.6 (s, C6), 120.6 (s, C5), 120.2 (s, C4), 110.3 (s, C2). ^31^P­{^1^H} NMR (162 MHz, CD_2_Cl_2_) δ 43.6 (s, 1P, PPh_3_). HRMS (ESI-QTOF) *m*/*z*: [M]^+^ Calculated for C_38_H_28_AuN_4_NaP; 791.1609; found 791.1626.

##### Complex **13**

**Method A:** 9-(4-ethynylphenyl)-9*H*-carbazole (29 mg, 0.11 mmol) is added to a solution of [Au­(N_3_)­(JohnPhos)] (58 mg, 0.11 mmol) in 5 mL of degassed toluene and under argon atmosphere. The resulting solution is stirred for 1 week at room temperature. After this reaction time, the volume of toluene is reduced to approximately 1 mL, and 10 mL of hexane is then added. The resulting precipitate is filtered and dried under vacuum. Complex **13** is obtained as a white solid (80 mg, 93%).

**Method B:** 9-(4-ethynylphenyl)-9*H*-carbazole (29 mg, 0.11 mmol) is added to a solution of [Au­(N_3_)­(JohnPhos)] (58 mg, 0.11 mmol) in a mixture of 6 mL of CH_2_Cl_2_ and 4 mL of acetonitrile, which has been previously purged with an argon stream for 10 min. [Cu­(NCMe)_4_]­PF_6_ (3 mg, 0.01 mmol) is then added. The resulting solution is stirred under argon atmosphere, at room temperature for 6 h. After this reaction time, the reaction mixture is filtered through silica and dried under vacuum. The obtained oil is dissolved in 1 mL of CH_2_Cl_2_, and complex **13** is finally isolated by precipitation with hexane, followed by filtration and dried under vacuum. Complex **13** is obtained as a white solid (72 mg, 84%).

^1^H NMR (400 MHz, CD_2_Cl_2_) δ 8.15 (d, *J* = 7.8 Hz, 2H, H5), 7.92 (td, *J* = 7.0, 1.8 Hz, 1H, JohnPhos), 7.61–7.47 (m, 7H, H8, JohnPhos), 7.47–7.37 (m, 6H, H9, H3, H2), 7.34–7.18 (m, 5H, H4, JohnPhos), 1.44 (d, *J* = 14.9 Hz, 18H, Me). ^13^C­{^1^H}-APT NMR (101 MHz, CD_2_Cl_2_) δ 150.5 (d, *J* = 15.1 Hz, JohnPhos), 143.2 (d, *J* = 6.1 Hz, JohnPhos), 141.3 (s, C1), 137.9 (d, *J* = 131.7 Hz, JohnPhos), 135.5 (s, C7), 135.0 (d, *J* = 1.3 Hz, JohnPhos), 133.5 (d, *J* = 1.3 Hz, C8), 133.4 (d, *J* = 7.3 Hz, JohnPhos), 130.7 (d, *J* = 2.2 Hz, JohnPhos), 129.7 (s, JohnPhos), 129.3 (s, JohnPhos), 128.2 (s, JohnPhos), 127.7 (d, *J* = 40.0 Hz, C12), 127.2 (d, *J* = 6.0 Hz, JohnPhos), 127.0 (s, C9), 126.3 (s, C3), 126.3 (s, C10), 123.7 (s, C6), 120.5 (s, C5), 120.3 (s, C4), 110.3 (s, C2), 101.3 (d, *J* = 23.4 Hz, C11), 37.8 (d, *J* = 22.6 Hz, *C*Me_3_), 31.2 (d, *J* = 7.0 Hz, *C*H_3_). ^31^P­{^1^H} NMR (162 MHz, CD_2_Cl_2_) δ 64.5 (s, 1P, JohnPhos). HRMS (ESI-QTOF) *m*/*z*: [M]^+^ Calculated for C_40_H_41_AuN_4_P: 805.2729; found 805.2758.

### Crystallography

Crystals were mounted on a MiTeGen Crystal micromount and transferred to the cold gas stream of a Bruker D8 VENTURE diffractometer. Data were collected using monochromated MoKα radiation (λ = 0.71073 Å). Scan type ϖ. Absorption corrections based on multiple scans were applied using SADABS.^42^ The structures were solved by direct methods and refined on F^2^ using the program SHELXT-2018.^43^ All non-hydrogen atoms were refined anisotropically.

## Supplementary Material



## Notes

All NMR spectra, all absorption, excitation, and emission spectra, TADF data, and TD-DFT calculations (PDF)
