# Supplementary material for: Alkynyl-Gold Carbazole Hybrids: Luminescence and Functionalization via iClick Reactions
Source: Inorg Chem. 2025 Aug 20;64(34):17399–408. doi: 10.1021/acs.inorgchem.5c02714 (PMC12406202; doi:10.1021/acs.inorgchem.5c02714)
Supplement: Supplementary file 1 [file ic5c02714_si_001.pdf]

SUPPORTING INFORMATION (SI)

Alkynyl-Gold Carbazole Hybrids: Luminescence and Functionalization *via* iClick Reactions

Roberto Berbés Martínez,[a,b] Juan V. Alegre-Requena,[a] Raquel P. Herrera,[b,*] M. Concepción Gimeno[a,*]

[a] Departamento de Química Inorgánica, Instituto de Síntesis Química y Catálisis Homogénea (ISQCH) CSIC-Universidad de Zaragoza, C/ Pedro Cerbuna 12, 50009 Zaragoza, Spain. gimeno@unizar.es

[b] Laboratorio de Organocatálisis Asimétrica, Departamento de Química Orgánica, Instituto de Síntesis Química y Catálisis Homogénea (ISQCH) CSIC-Universidad de Zaragoza, C/ Pedro Cerbuna 12, 50009 Zaragoza, Spain. raquelph@unizar.es

CONTENTS

1. Synthesis of starting precursors S2

2. Synthesis and characterization of gold complexes 1-10, 12 and 13 ....S2

3. ^{1}H, $^{13}C\{^{1}H\}$-APT and $^{31}P\{^{1}H\}$ NMR spectra S9

4. Temperature-dependent NMR study of complex 10 ....S25

5. Absorption, excitation and emission spectra S26

6. Complex 2 TADF graphic and lifetimes table S39

7. Computational methods S40

8. Bibliography S44

1. Synthesis of starting precursors

Synthesis of [AuCl(L)] (L = PR_3, CNCy) precursors

To a solution of [AuCl(tht)] (1 mmol) in dichloromethane (10 mL) is added 1 equiv. of the chosen phosphine. The mixture is stirred for 30 min and then, hexane (15 mL) is added to obtain the desired product as a white precipitate, which is filtered and washed with more hexane. After drying them under vacuum, the products are obtained in quantitative yield (>95%) and used without further purification.

Synthesis of the polymer [AuC≡CR]n (C≡CR=9-(4-ethynylphenyl)-9*H*-carbazole)

9-(4-Ethynylphenyl)-9*H*-carbazole (133 mg, 0.5 mmol) is added to a solution of [AuCl(tht)] (160 mg, 0.5 mmol) in CH_2Cl_2 (20 mL). The resulting yellow solution is stirred for 5 minutes, after which triethylamine (0.1 mL) is added, leading to the formation of a yellow precipitate. The mixture is then stirred for an additional hour. The desired product is obtained in a 49% yield (108 mg) by filtration, followed by successive washes with methanol (3 × 5 mL) and diethyl ether (3 × 5 mL).

Synthesis of [Au(N_3)(PR_3)] derivatives

To a solution of [AuCl(PR_3)] (1 mmol) in toluene (10 mL), AgOAc (167 mg, 1 mmol) is added, and the resulting solution is stirred, protected from sunlight, for 12 hours at room temperature. After this reaction time, the solution is passed through a Celite filter, which is then washed with an excess of toluene (3 x 5 mL). $TMSN_3$ (1 mmol) is then added to the resulting solution and stirred for another 12 hours at room temperature. After the reaction time, the volume is reduced by half under vacuum, and the desired product is precipitated with cold hexane, isolated by filtration and dried under vacuum.

2. Synthesis and characterization of gold complexes 1-10, 12 and 13

Synthesis of alkynyl gold complexes 1-6 and 9

For complexes **1-6** and **9**: To a solution of KOH (0.3 mmol, 1.5 equiv) in MeOH (10 mL), the alkyne 9-(4-ethynylphenyl)-9*H*-carbazole (0.2 mmol, 1 equiv), and [AuCl(L)] (0.2 mmol, 1 equiv) were added. The mixture was stirred overnight at room temperature, and the resulting precipitate was isolated by filtration. The precipitate was then washed with cold methanol (3 x 5 mL) and diethyl ether (3 x 5 mL), and dried under vacuum.

For complex **9**, 2 equivalents of the ligand 9-(4-ethynylphenyl)-9*H*-carbazole are used instead of 1, and 3 equivalents of KOH instead of 1.5.

Complex 1

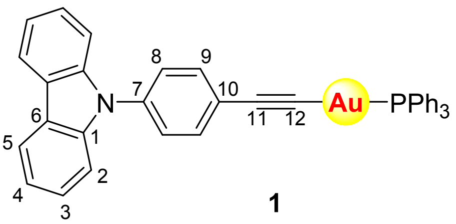

1

Following the general procedure, complex **1** was obtained after 12 hours of reaction, as a pale white solid (116 mg, 80% yield). ^{1}H NMR (400 MHz, $CDCl_3$) δ 8.13 (d, *J* = 7.8 Hz, 2H, H5), 7.73 (d, *J* = 8.5 Hz, 2H, H8), 7.62–7.37 (m, 21H, PPh_3, H3, H2, H9), 7.30–7.25 (m, 2H, H4). $^{13}C\{^1H\}$-APT NMR (101 MHz, $CDCl_3$) δ 140.9 (s, C1), 136.2 (s, C7), 134.5 (d, *J* = 13.8 Hz, Ph), 133.9 (s, C8), 133.5 (d, *J* = 145.4 Hz, P-C), 131.7 (d, *J* = 2.4 Hz, Ph), 129.8 (d, *J* = 54.5 Hz, C12), 129.3 (d, *J* = 11.3 Hz, Ph), 126.7 (s, C9), 126.1 (s, C3), 124.2 (d, *J* = 3.0 Hz, C10), 123.5 (s, C6), 120.3 (s, C5), 120.0 (s, C4), 110.0 (s, C2), 103.5 (d, *J* = 26.3 Hz, C11). $^{31}P\{^1H\}$ NMR (162 MHz, $CDCl_3$) δ 42.4 (s, 1P, PPh_3). HRMS (ESI-QTOF) m/z: $[M+Na]^+$ Calculated for $C_{38}H_{27}AuNNaP$ 748.1439; found 748.1439.

Complex 2

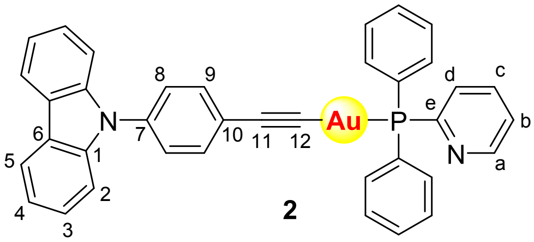

2

Following the general procedure, complex **2** was obtained after 12 hours of reaction, as a pale-yellow solid (137 mg, 94% yield). ^{1}H NMR (400 MHz, $CDCl_3$) δ 8.82 (d, *J* = 4.3 Hz, 1H, Ha), 8.16 (d, *J* = 7.7 Hz, 2H, H5), 8.06 (t, *J* = 7.6 Hz, 1H, Hd), 7.84–7.74 (m, 7H, Hc, H8, Ph), 7.57–7.37 (m, 13H, Hb, H3, H2, H9, Ph), 7.33–7.28 (m, 2H, H4). $^{13}C\{^1H\}$-APT NMR (101 MHz, $CDCl_3$) δ 155.3 (d, *J* = 77.1 Hz, Ce), 151.5 (d, *J* = 14.5 Hz, Ca), 140.8 (s, C1), 136.6 (d, *J* = 11.0 Hz, Cc), 136.2 (s, C7), 134.8 (d, *J* = 13.8 Hz, Ph), 133.9 (s, C8), 131.9 (d, *J* = 33.1 Hz, Cd), 131.8 (d, *J* = 2.3 Hz, Ph), 129.5 (d, *J* = 57.0 Hz, C12), 129.1 (d, *J* = 11.5 Hz, Ph), 126.7 (s, C9), 126.1 (s, C3), 125.3 (d, *J* = 2.2 Hz, Cb), 124.2 (s, C10), 123.5 (s, C6), 120.4 (s, C5), 120.1 (s, C4), 110.0 (s, C2), 103.5 (s, C11). $^{31}P\{^1H\}$ NMR (121 MHz, $CDCl_3$) δ 41.2 (s, 1P, PPh_2Py). HRMS (ESI-QTOF) m/z: $[M+Na]^+$ Calculated for $C_{37}H_{26}AuN_2NaP$ 749.1391; found 749.1391.

Complex 3

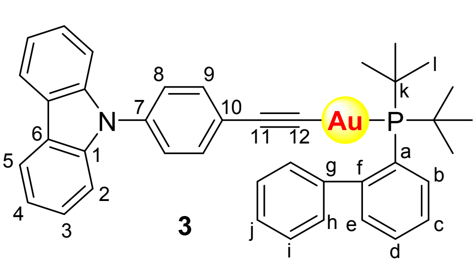

3

Following the general procedure, complex **3** was obtained after 12 hours of reaction, as a pale-yellow solid (143 mg, 93% yield). ^{1}H NMR (400 MHz, $CDCl_3$) δ 8.14 (d, *J* = 7.7 Hz, 2H, H5), 7.89 (td, *J* = 7.1, 1.5 Hz, 1H, JohnPhos), 7.71–7.65 (m, 2H, H8), 7.65–7.38 (m, 11H, JohnPhos, H2, H3, H9), 7.34–7.16 (m, 5H, JohnPhos, H4), 1.45 (d, *J* = 15.0 Hz, 18H, CH_3). $^{13}C\{^1H\}$-APT NMR (101 MHz, $CDCl_3$) δ 150.4 (d, *J* = 15.0 Hz, JohnPhos), 142.6 (d, *J* = 6.1 Hz, JohnPhos), 141.0 (s, C1), 135.9 (d, *J* = 131.5 Hz, Ca), 135.4 (s, C7), 134.5 (d, *J* = 1.2 Hz, JohnPhos), 133.6 (s, C8), 133.2 (d, *J* = 7.3 Hz, JohnPhos), 130.4 (d, *J* = 2.1 Hz, JohnPhos), 129.4 (s, JohnPhos), 129.2 (s, JohnPhos), 128.2 (s, JohnPhos), 127.6 (d, *J* = 39.9 Hz, C12), 126.8 (d, *J* = 5.9 Hz, JohnPhos), 126.5 (s,

C9), 126.0 (s, C3), 125.6 (d, J = 2.4 Hz, C10), 123.5 (s, C6), 120.3 (s, C5), 119.9 (s, C4), 110.1 (s, C2), 101.6 (d, J = 23.2 Hz, C11), 37.7 (d, J = 22.4 Hz, CMe$_3$), 31.2 (d, J = 7.0 Hz, CH$_3$). ^{31}P{^{1}H} NMR (162 MHz, CDCl$_3$) δ 64.5 (s, 1P, JohnPhos). HRMS (ESI-QTOF) m/z: [M+Na]$^+$ Calculated for $C_{40}H_{39}AuNNaP$ 784.2378; found 784.2378.

Complex 4

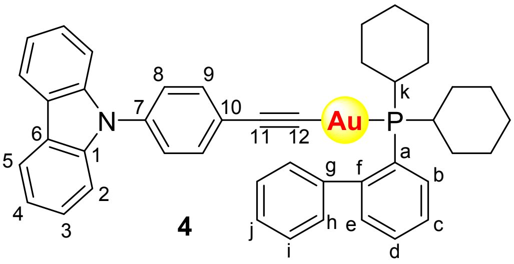

4

Following the general procedure, complex **4** was obtained after 12 hours of reaction, as a white solid (160 mg, 98% yield). ^{1}H NMR (300 MHz, CDCl$_3$) δ 8.14 (d, J = 7.7 Hz, 2H, H5), 7.88–7.78 (m, 1H, JohnPhos), 7.75–7.66 (m, 2H, H8), 7.61–7.36 (m, 11H, JohnPhos, H2, H3, H9), 7.35–7.16 (m, 5H, JohnPhos, H4), 2.22–2.06 (m, 2H, Hk), 2.05–1.92 (m, 2H, H^{Cy}), 1.88–1.50 (m, 10H, H^{Cy}), 1.46–1.11 (m, 8H, H^{Cy}). ^{13}C{^{1}H}-APT NMR (101 MHz, CDCl$_3$) δ 149.0 (d, J = 10.1 Hz, JohnPhos), 141.8 (d, J = 5.0 Hz, JohnPhos), 140.9 (s, C1), 136.9 (d, J = 135.3 Hz, Ca), 135.6 (s, C7), 135.2 (d, J = 9.1 Hz, JohnPhos), 133.7 (s, C8), 132.4 (d, J = 7.1 Hz, JohnPhos), 130.6 (d, J = 2.0 Hz, JohnPhos), 129.3 (s, JohnPhos), 128.9 (s, JohnPhos), 128.3 (s, JohnPhos), 127.5 (d, J = 9.1 Hz, JohnPhos), 126.5 (s, C9), 126.4 (d, J = 46.5 Hz, C12), 126.0 (s, C3), 125.1 (d, J = 2.0 Hz, C10), 123.4 (s, C6), 120.3 (s, C5), 119.9 (s, C4), 110.0 (s, C2), 102.0 (s, d, J = 25.3 Hz, C11), 36.6 (d, J = 30.1 Hz, CH^{Cy}), 31.2 (d, J = 5.3 Hz, CH$_2^{Cy}$), 29.6 (s, CH$_2^{Cy}$), 26.8 (d, J = 4.4 Hz, CH$_{2Cy}$), 26.7 (d, J = 6.6 Hz, CH$_{2Cy}$), 25.7 (s, CH$_2^{Cy}$). ^{31}P{^{1}H} NMR (121 MHz, CDCl$_3$) δ 50.4 (s, 1P, ^{Cy}JohnPhos). HRMS (ESI-QTOF) m/z: [M+Na]$^+$ Calculated for $C_{44}H_{43}AuNNaP$ 836.2691; found 836.2691.

Complex 5

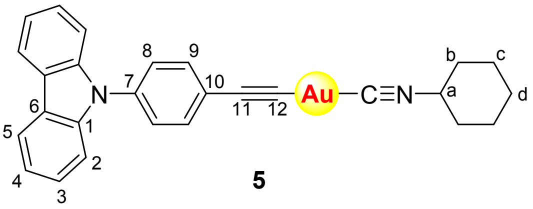

5

Following the general procedure, compound **5** was obtained after 12 hours of reaction, as a white solid (93 mg, 82% yield). ^{1}H NMR (400 MHz, CDCl$_3$) δ 8.13 (d, J = 7.7 Hz, 2H, H5), 7.68 (d, J = 8.4 Hz, 2H, H8), 7.45 (d, J = 8.5 Hz, 2H, H9), 7.43–7.36 (m, 4H, H2, H3), 7.32 – 7.23 (m, 2H, H4), 3.88 (tt, J = 8.1, 3.8 Hz, 1H, CH-NC), 2.07–1.95 (m, 2H, H^{Cy}), 1.88–1.69 (m, 4H, H^{Cy}), 1.57–1.40 (m, 4H, H^{Cy}). ^{13}C{^{1}H}-APT NMR (101 MHz, CDCl$_3$) δ 140.9 (s, C1), 136.3 (s, C7), 134.0 (s, C8), 126.7 (s, C9), 126.1 (s, C3), 124.0 (s, C10), 123.5 (s, C6), 123.4 (s, C12), 120.4 (s, C5), 120.1 (s, C4), 110.0 (s, C2), 102.7 (s, C11), 54.8 (s, Ca), 31.7 (s, C^{Cy}), 24.7 (s, C^{Cy}), 22.7 (s, C^{Cy}). HRMS (ESI-QTOF) m/z: [M+H]$^+$ Calculated for $C_{27}H_{24}AuN_2$ 573.1600; found 573.1573.

Complex 6

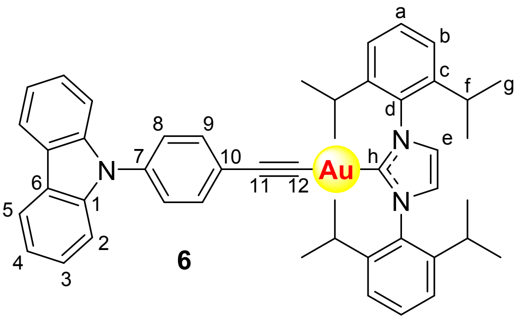

Following the general procedure, complex **6** was obtained after 12 hours of reaction, as a white solid (121 mg, 71% yield). ^{1}H NMR (300 MHz, $CDCl_3$) δ 8.03 (d, *J* = 7.7 Hz, 2H, H5), 7.51–7.42 (m, 4H, H8, Ha), 7.33–7.13 (m, 12H, H9+H4+H3+H2+Hb), 7.07 (s, 2H, He), 2.57 (hept, *J* = 6.8 Hz, 4H, Hf), 1.35 (d, *J* = 6.8 Hz, 12H), 1.17 (d, *J* = 6.9 Hz, 12H). $^{13}C\{^1H\}$-APT NMR (101 MHz, $CDCl_3$) δ 191.0 (s, Ch), 145.8 (s, Cd), 140.9 (s, C1), 135.1 (s, C7), 134.4 (s, Cc), 133.7 (s, C8), 131.2 (s, C12), 130.6 (s, Ca), 126.4 (s, C9), 125.9 (s, C3), 125.5 (s, C10), 124.3 (s, Cb), 123.4 (s, Ce), 123.4 (s, C6), 120.3 (s, C5), 119.8 (s, C4), 110.0 (s, C2), 103.1 (s, C11), 29.0 (s, Cf), 24.8 (s, Cg), 24.2 (s, Cg). HRMS (ESI-QTOF) m/z: $[M+H]^+$ Calculated for $C_{47}H_{49}AuN_3$ 852.3586; found 852.3587.

Complex 7

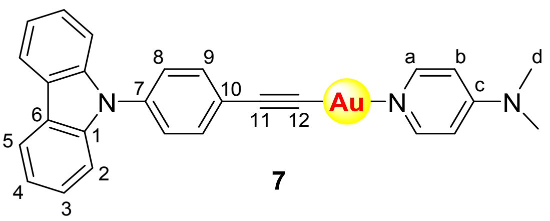

The polymer $[AuC{\equiv}CR]_n$ (66 mg, 0.15 mmol) is added to a solution of *N*,*N*-dimethylpyridin-4-amine (18 mg, 0.15 mmol) in CH_2Cl_2 (5 mL), and the resulting solution is stirred for 2 hours. After the reaction time, the reaction volume is reduced to 1 mL, and hexane (10 mL) is added to obtain the product as a white solid (78 mg, 92%), which is then filtered and dried under vacuum. ^{1}H NMR (400 MHz, CD_2Cl_2) δ 8.14 (d, *J* = 7.7 Hz, 2H, H5), 8.05 (d, *J* = 6.5 Hz, 2H, Ha), 7.60 (d, *J* = 8.2 Hz, 2H, H8), 7.50–7.36 (m, 6H, H2, H3, H9), 7.32– 7.24 (m, 2H, H4), 6.59 (d, *J* = 6.5 Hz, 2H, Hb), 3.08 (s, 6H, Hd). $^{13}C\{^1H\}$-APT NMR (101 MHz, $CDCl_3$) δ 150.4 (s, Ca), 141.0 (s, C1), 134.0 (s C8), 126.6 (s, C9), 126.0 (s, C3), 123.5 (s, C6), 120.3 (s, C5), 120.0 (s, C4), 110.1 (s, C2), 107.6 (s, Cb), 39.5 (s, Cd). HRMS (ESI-QTOF) m/z: $[M+Na]^+$ Calculated for $C_{27}H_{22}AuN_3Na$ 608.1372; found 608.1388.

Complex 8

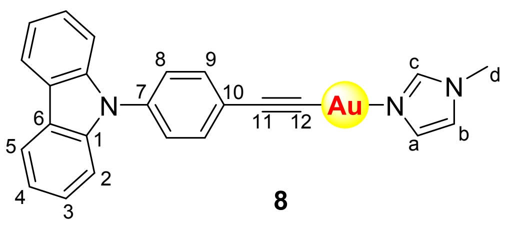

The polymer $[AuC{\equiv}CR]_n$ (49 mg, 0.11 mmol) is added to a solution of 1-methyl-1*H*-imidazole (9 mg, 0.11 mmol) in CH_2Cl_2 (5 mL), and the resulting solution is stirred for 2 hours. After the reaction time, the reaction volume is reduced to 1 mL, and hexane (10 mL) is added to obtain the product as a white solid (36 mg, 61%), which is then filtered and dried under vacuum. ^{1}H NMR (300 MHz, $CDCl_3$) δ 8.13 (d, *J* = 7.7 Hz, 2H, H5), 7.75–7.64 (m, 3H, H8, Hc), 7.51–7.36 (m, 6H, H9, H3, H2), 7.33–7.26 (m, 2H, H4), 7.13 (s, 1H, H^{Im}), 7.08 (s, 1H, H^{Im}), 3.82 (s, 3H, Hd). The solubility of compound **8** is too low to determine its $^{13}C\{^1H\}$-APT NMR spectrum. HRMS (ESI-QTOF) m/z: $[M+Na]^+$ Calculated for $C_{24}H_{18}AuN_3Na$ 568.1058; found 568.1038.

Complex 9

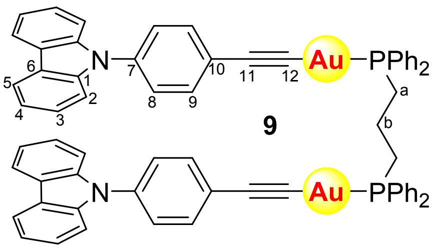

For this complex, 2 equivalents of 9-(4-ethynylphenyl)-9*H*-carbazole are added to the solution instead of one. Following the general procedure, complex **9** was obtained after 12 hours of reaction, as a pale white solid (209 mg, 78% yield). ^{1}H NMR (400 MHz, $CDCl_3$) δ 8.13 (d, *J* = 7.7 Hz, 4H, H5), 7.78– 7.60 (m, 12H, H8, Ph), 7.54–7.34 (m, 24H, H9, H3, H2, Ph), 7.32–7.26 (m, 4H, H4), 2.52–2.34 (m, 4H, Ha), 1.74–1.62 (m, 2H, Hb). ^{13}C{^{1}H}-APT NMR (101 MHz, $CDCl_3$) δ 140.8 (s, C1), 136.2 (s, C7), 133.8 (s, C8), 133.5 (d, *J* = 13.5 Hz, Ph), 131.5 (s, Ph), 129.3 (d, *J* = 10.5 Hz, Ph), 126.7 (s, C9), 126.0 (s, C3), 124.2 (s, C10), 123.5 (s, C6, C12), 120.4 (s, C5), 120.1 (s, C4), 110.0 (s, C2), 103.5 (s, C11), 27.8 (d, *J* = 31.1 Hz, Ca), 24.9 (d, *J* = 2.9 Hz, Cb). ^{31}P{^{1}H} NMR (162 MHz, $CDCl_3$) 35.7 (s, 2P, PPh_2CH_2). HRMS (ESI-QTOF) m/z: $[M+Na]^+$ Calculated for $C_{67}H_{50}Au_2N_2NaP_2$ 1361.2672; found 1361.2673.

Complex 10

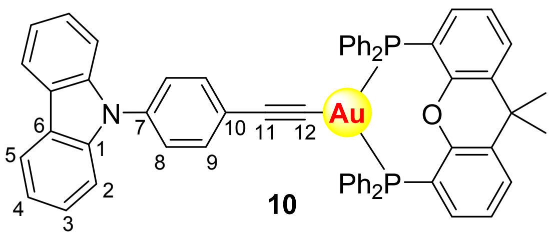

The polymer $[AuC{\equiv}CR]_n$ (49 mg, 0.10 mmol) is added to a solution of xanthphos (58 mg, 0.10 mmol) in toluene (5 mL), and the resulting solution is stirred for 2 hours. After the reaction time, the reaction volume is reduced to 1 mL, and hexane (10 mL) is added to obtain the product **10** as a white solid (93 mg, 61%), which is then filtered and dried under vacuum. ^{1}H NMR (300 MHz, toluene-d_8) δ 8.01 (d, *J* = 7.6 Hz, 2H, H5), 7.70 (d, *J* = 8.5 Hz, 2H, H8), 7.54–7.36 (m, 8H, Ph), 7.35–7.14 (m, 8H, H4, H3, H2, Ph), 7.08 –7.02 (m, 4H, H9, xanthphos), 6.96–6.86 (m, 10H, Ph), 6.77–6.65 (m, 4H, xanthphos), 1.33 (s, 6H, Me). ^{13}C{^{1}H}-APT NMR (101 MHz, toluene-d_8) δ 153.4 (app. t, *J* = 7.1 Hz, xanthphos), 141.5 (s, C1), 135.3 (s, C7), 134.7 (d, *J* = 10.1 Hz, xanthphos), 134.5 (d, *J* = 17.2 Hz, xanthphos), 134.5 (s, xanthphos), 133.7 (s, C8), 132.9 (s, xanthphos), 131.1 (s, xanthphos), 129.6 (s, xanthphos), 127.3 (s, xanthphos), 126.8 (s, C9), 126.1 (s, C3), 124.4 (s, xanthphos), 124.0 (s, C6), 122.2 (app. t, *J* = 8.1 Hz, xanthphos), 120.4 (s, C5), 120.0 (s, C4), 110.4 (s, C2), 104.1 (s, C11), 34.7 (s, $C(CH_3)$), 30.8 (s, Me). ^{31}P{^{1}H} NMR (162 MHz, toluene-d_8) δ 4.5 (s, 2P, xanthphos). HRMS (ESI-QTOF) m/z: $[M+Na]^+$ Calculated for $C_{59}H_{44}AuNNaOP_2$ 1064.2461; found 1064.2456.

Complex 12

Method A: 9-(4-ethynylphenyl)-9*H*-carbazole (25 mg, 0.1 mmol) is added to a solution of $[Au(N_3)(PPh_3)]$ (47 mg, 0.1 mmol) in 5 mL of degassed toluene and under argon atmosphere. The resulting solution is stirred for one week at room temperature. After this reaction time, the volume of toluene is reduced to approximately 1 mL, and 10 mL of hexane is then added. The resulting precipitate is filtered under vacuum and final complex **12** is obtained as a white solid (57 mg, 80%).

Method B: 9-(4-ethynylphenyl)-9*H*-carbazole (25 mg, 0.1 mmol) is added to a solution of [Au(N_3)(PPh_3)] (47 mg, 0.1 mmol) in a mixture of 6 mL of CH_2Cl_2 and 4 mL of acetonitrile, which has been previously purged with an argon stream for 10 minutes. [Cu(NCMe)$_4$]PF_6 (3 mg, 0.01 mmol) is then added. The resulting solution is stirred under argon atmosphere, at room temperature for 6 hours. After this reaction time, the reaction mixture is filtered through silica and dried under vacuum. The obtained oil is dissolved in 1 mL of CH_2Cl_2, and complex **12** is finally isolated by precipitation with hexane, followed filtration under vacuum. Complex **12** is obtained as a white solid (52 mg, 73%).

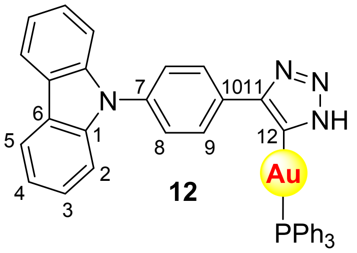

12

^{1}H NMR (400 MHz, CD_2Cl_2) δ 11.96 (br s, 1H, NH), 8.58 (d, *J* = 8.4 Hz, 2H, H8), 8.16 (d, *J* = 7.8 Hz, 2H, H5), 7.72–7.58 (m, 6H, Ph), 7.57– 7.37 (m, 15H, Ph, H9, H3, H2), 7.36–7.23 (m, 2H, H4). ^{13}C{^{1}H}-APT NMR (101 MHz, CD_2Cl_2) δ 152.9 (s, Cq), 141.4 (s, C1), 136.3 (s, C7), 134.7 (d, *J* = 13.8 Hz, Ph), 134.7 (s, (s, Cq), 132.2 (d, *J* = 2.1 Hz, Ph), 130.4 (s, Cq), 129.7 (d, *J* = 11.2 Hz, Ph), 128.0 (s, C8), 127.3 (s, C9), 126.3 (s, C3), 123.6 (s, C6), 120.6 (s, C5), 120.2 (s, C4), 110.3 (s, C2). ^{31}P{^{1}H} NMR (162 MHz, CD_2Cl_2) δ 43.6 (s, 1P, PPh_3). HRMS (ESI-QTOF) m/z: [M]$^+$ Calculated for $C_{38}H_{28}AuN_4NaP$; 791.1609; found 791.1626.

Complex 13

Method A: 9-(4-ethynylphenyl)-9*H*-carbazole (29 mg, 0.11 mmol) is added to a solution of [Au(N_3)(JohnPhos)] (58 mg, 0.11 mmol) in 5 mL of degassed toluene and under argon atmosphere. The resulting solution is stirred for one week at room temperature. After this reaction time, the volume of toluene is reduced to approximately 1 mL, and 10 mL of hexane is then added. The resulting precipitate is filtered under vacuum and final complex **13** is obtained as a white solid (80 mg, 93%).

Method B: 9-(4-ethynylphenyl)-9*H*-carbazole (29 mg, 0.11 mmol) is added to a solution of [Au(N_3)(JohnPhos)] (58 mg, 0.11 mmol) in a mixture of 6 mL of CH_2Cl_2 and 4 mL of acetonitrile, which has been previously purged with an argon stream for 10 minutes. [Cu(NCMe)$_4$]PF_6 (3 mg, 0.01 mmol) is then added. The resulting solution is stirred under argon atmosphere, at room temperature for 6 hours. After this reaction time, the reaction mixture is filtered through silica and dried under vacuum. The obtained oil is dissolved in 1 mL of CH_2Cl_2, and complex **13** is finally isolated by precipitation with hexane, followed filtration under vacuum. Complex **13** is obtained as a white solid (72 mg, 84%).

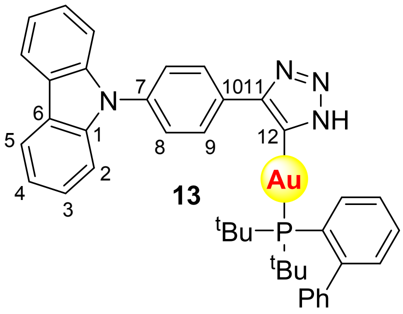

13

^{1}H NMR (400 MHz, CD_2Cl_2) δ 8.15 (d, *J* = 7.8 Hz, 2H, H5), 7.92 (td, *J* = 7.0, 1.8 Hz, 1H, JohnPhos), 7.61–7.47 (m, 7H, H8, JohnPhos), 7.47–7.37 (m, 6H, H9, H3, H2), 7.34–7.18 (m, 5H, H4, JohnPhos), 1.44 (d, *J* = 14.9 Hz, 18H, Me). ^{13}C{^{1}H}-APT NMR (101 MHz, CD_2Cl_2) δ 150.5 (d, *J* = 15.1 Hz, JohnPhos), 143.2 (d, *J* = 6.1 Hz, JohnPhos), 141.3 (s, C1), 137.9 (d, *J* = 131.7 Hz, JohnPhos), 135.5 (s, C7), 135.0 (d, *J* = 1.3 Hz, JohnPhos), 133.5 (d, *J* = 1.3 Hz, C8), 133.4 (d, *J* = 7.3 Hz, JohnPhos), 130.7 (d, *J* = 2.2 Hz, JohnPhos), 129.7 (s, JohnPhos), 129.3 (s, JohnPhos),

128.2 (s, JohnPhos), 127.7 (d, J = 40.0 Hz, C12), 127.2 (d, J = 6.0 Hz, JohnPhos), 127.0 (s, C9), 126.3 (s, C3), 126.3 (s, C10), 123.7 (s, C6), 120.5 (s, C5), 120.3 (s, C4), 110.3 (s, C2), 101.3 (d, J = 23.4 Hz, C11), 37.8 (d, J = 22.6 Hz, CMe$_3$), 31.2 (d, J = 7.0 Hz, CH$_3$). ^{31}P{^{1}H} NMR (162 MHz, CD_2Cl_2) δ 64.5 (s, 1P, JohnPhos). HRMS (ESI-QTOF) m/z: $[M]^+$ Calculated for $C_{40}H_{41}AuN_4P$: 805.2729; found 805.2758.

3. ^{1}H, $^{13}C\{^{1}H\}$-APT and $^{31}P\{^{1}H\}$ NMR spectra

Figure S1. ^{1}H NMR (400 MHz, $CDCl_3$) spectrum of gold complex **1**

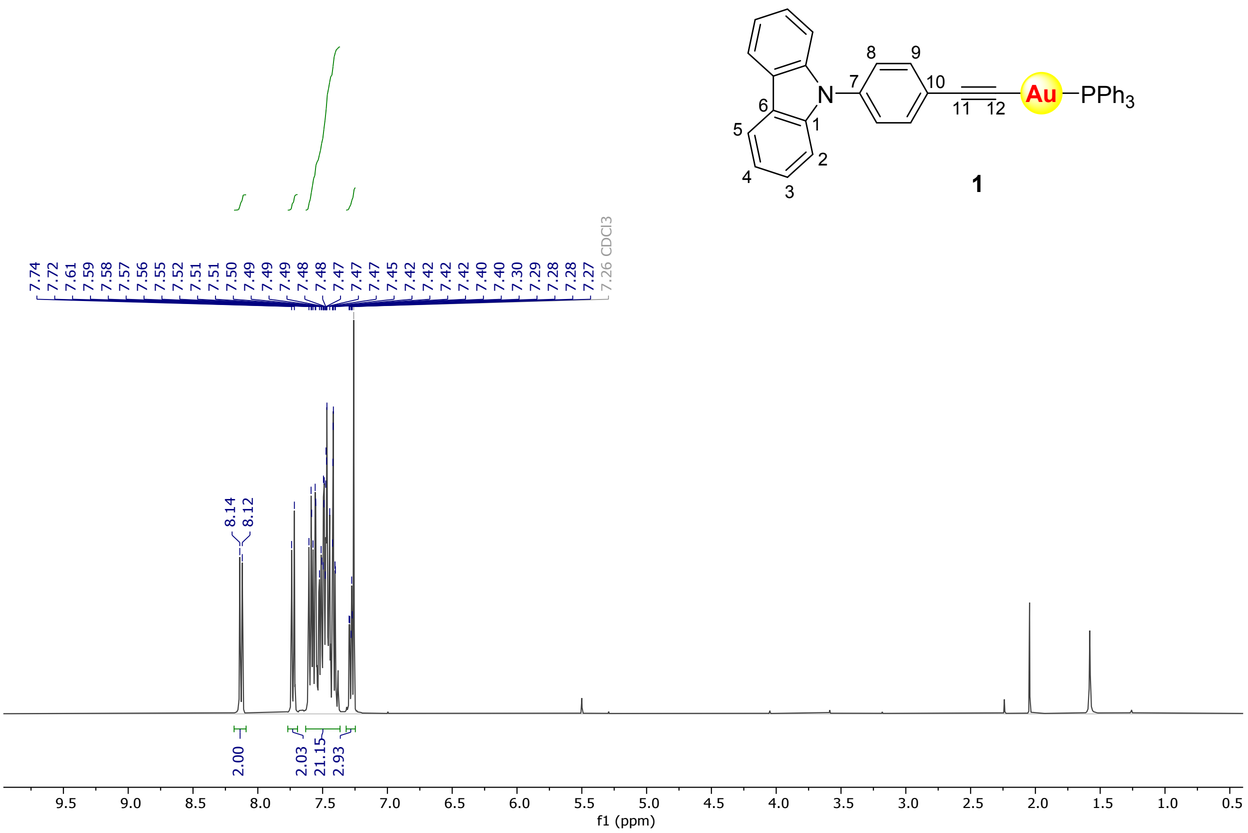

Figure S2. $^{13}C\{^{1}H\}$-APT NMR (101 MHz, $CDCl_3$) spectrum of gold complex **1**

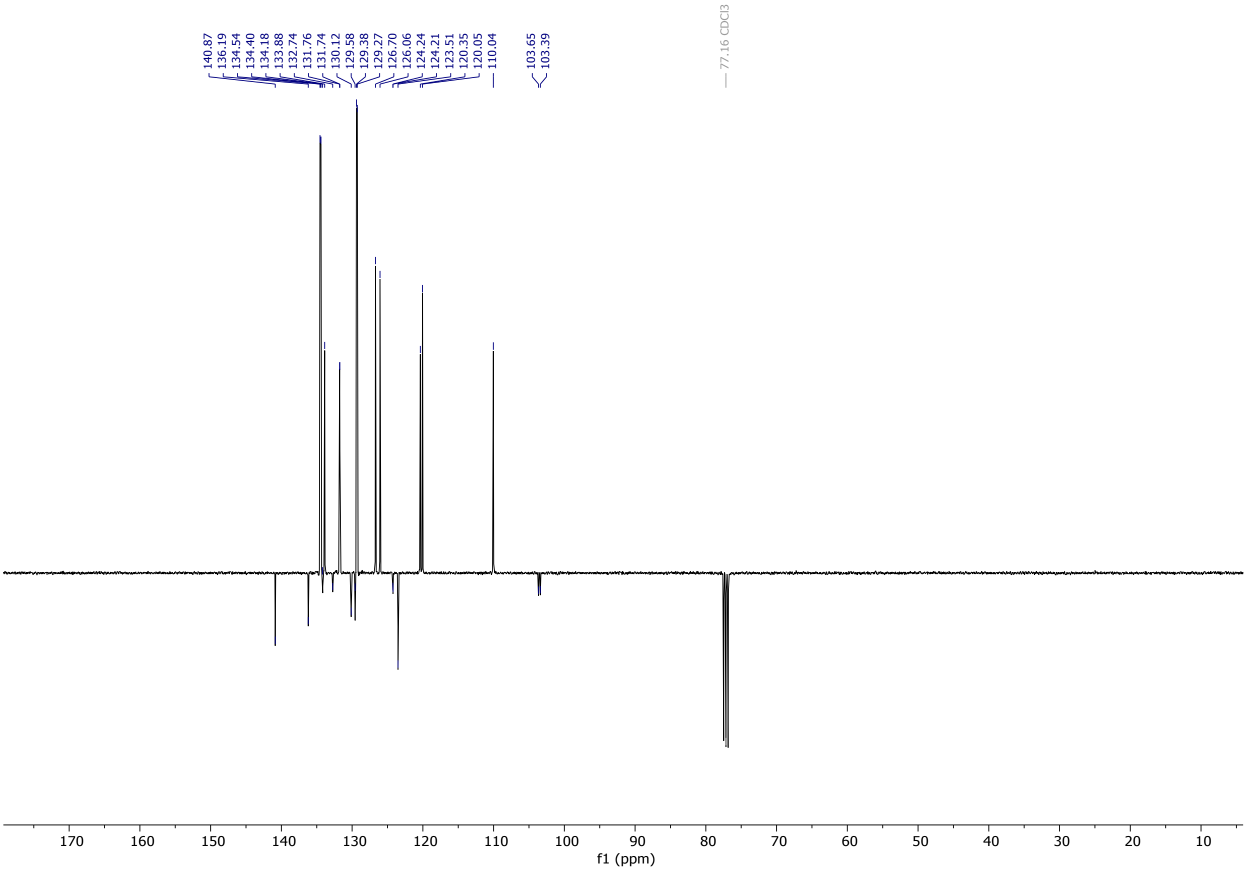

Figure S3. $^{31}P\{^{1}H\}$ NMR (162 MHz, $CDCl_3$) spectrum of gold complex **1**

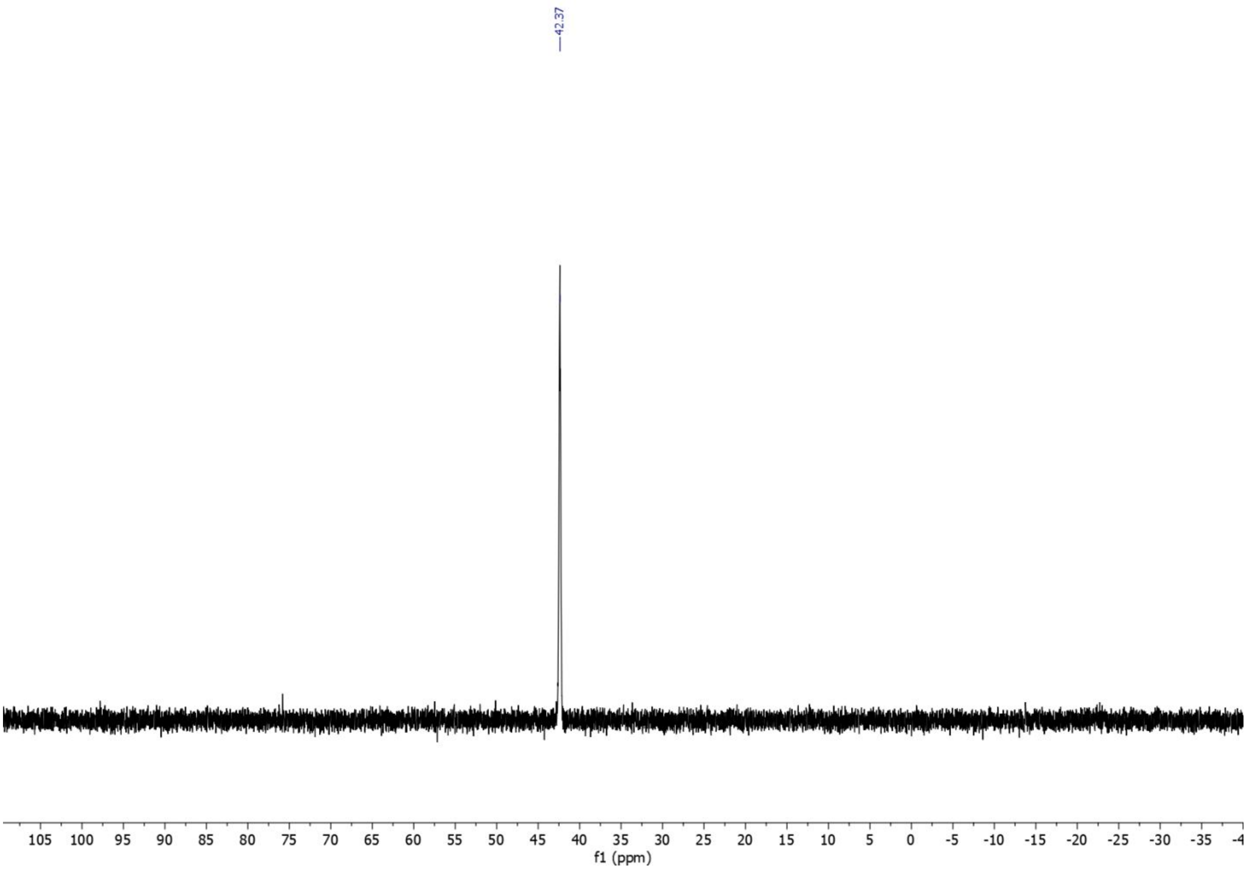

Figure S4. ^{1}H NMR (400 MHz, $CDCl_3$) spectrum of gold complex **2**

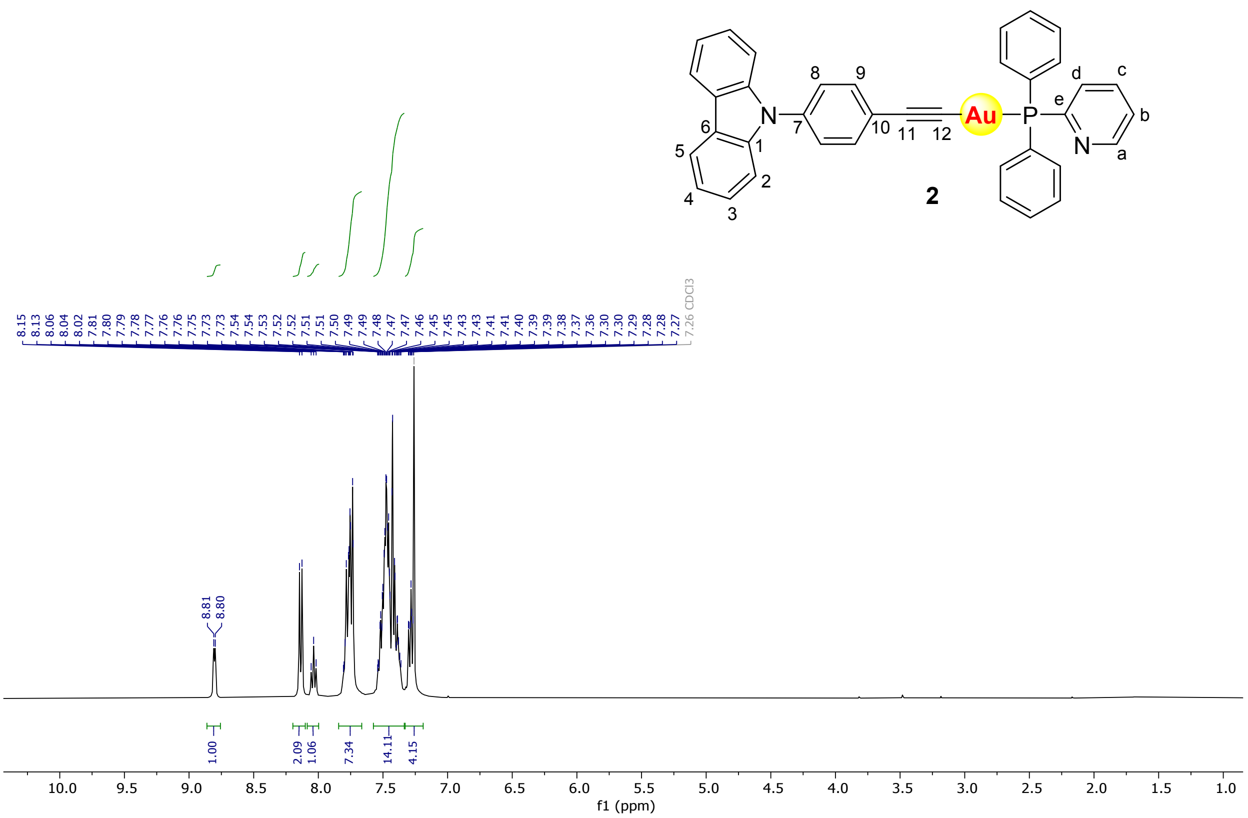

Figure S5. $^{13}C\{^{1}H\}$-APT NMR (101 MHz, $CDCl_3$) spectrum of gold complex **2**

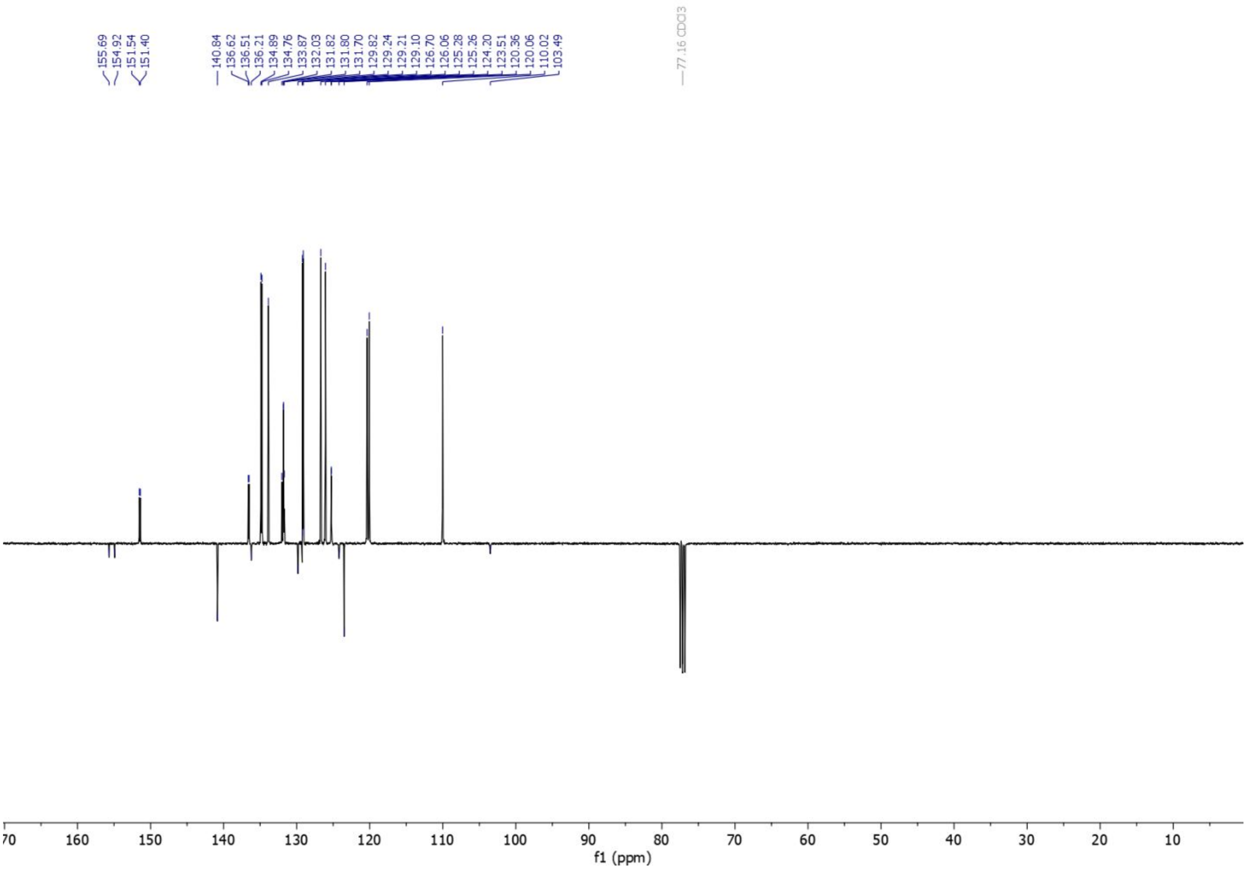

Figure S6. $^{31}P\{^{1}H\}$ NMR (121 MHz, $CDCl_3$) spectrum of gold complex **2**

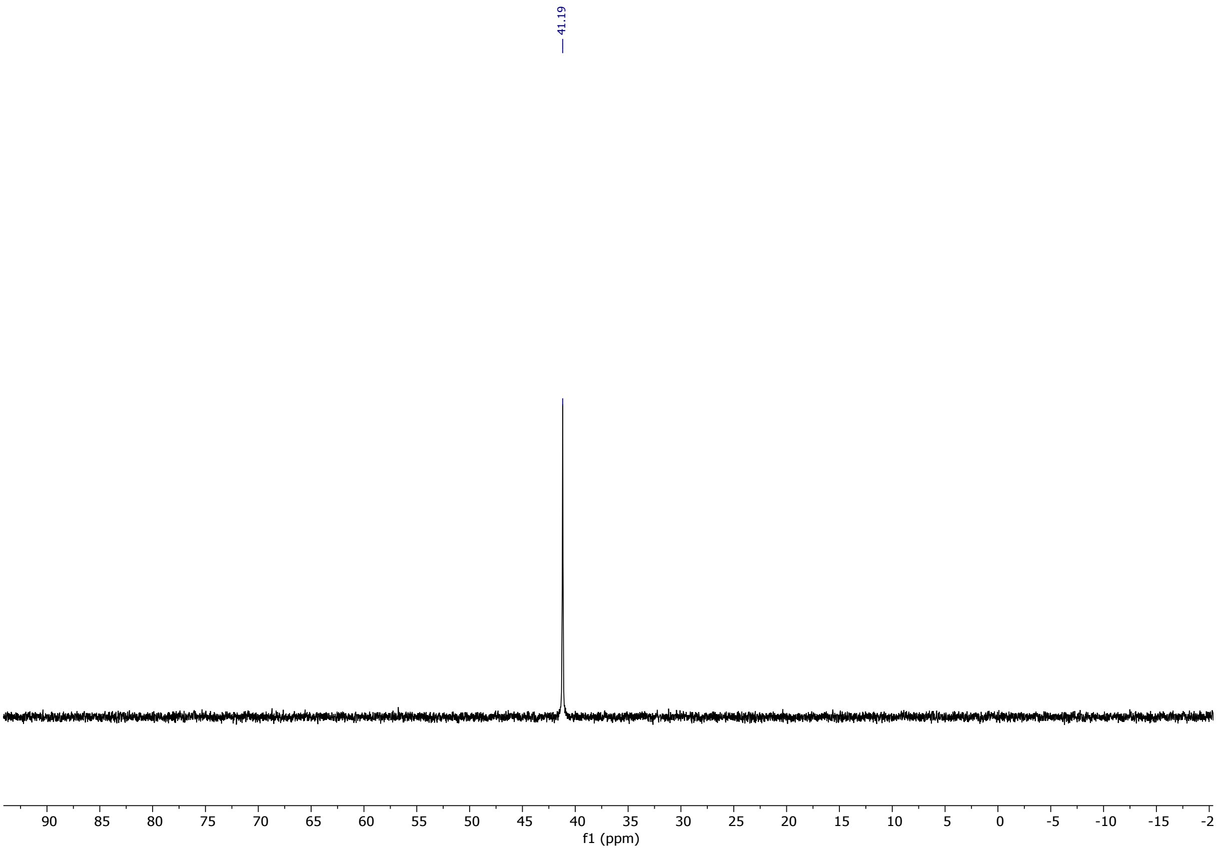

Figure S7. ^{1}H NMR (400 MHz, $CDCl_3$) spectrum of gold complex **3**

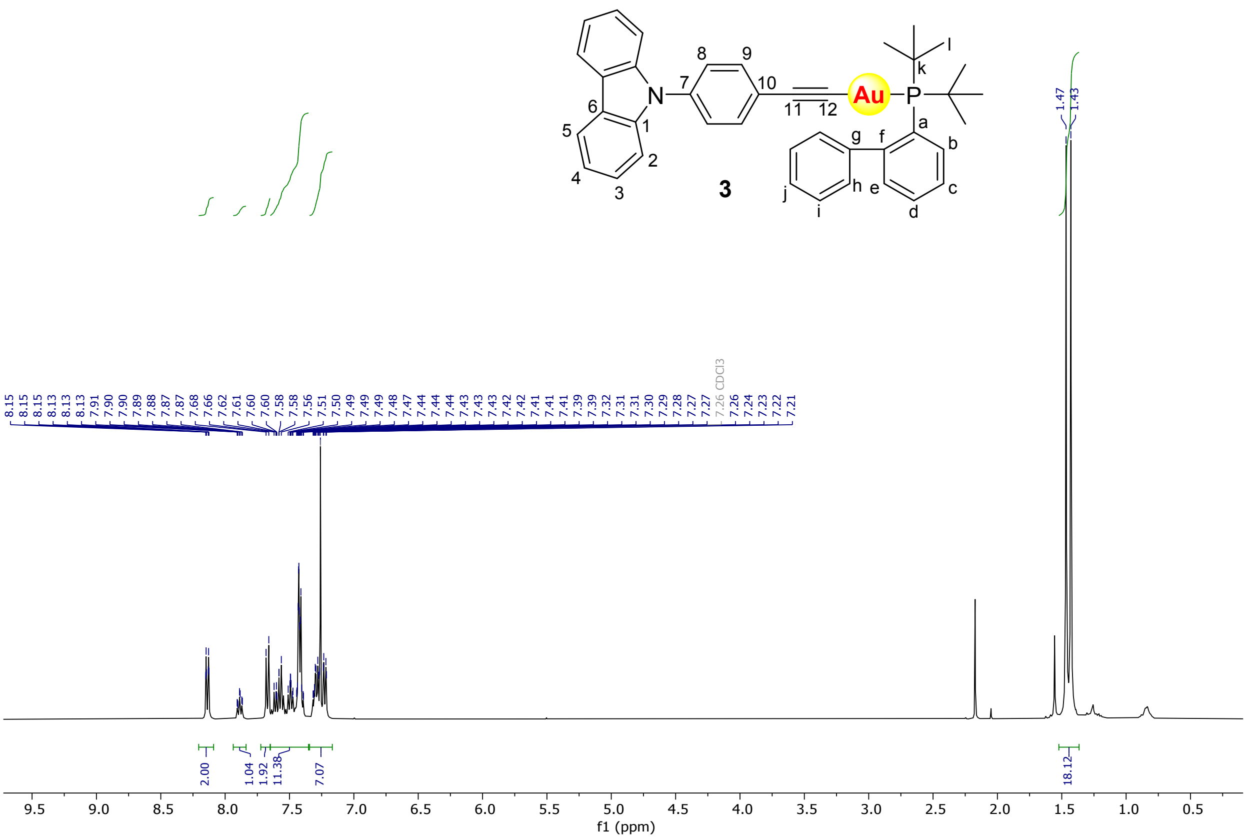

Figure S8. ^{13}C{^{1}H}-APT NMR (101 MHz, $CDCl_3$) spectrum of gold complex **3**

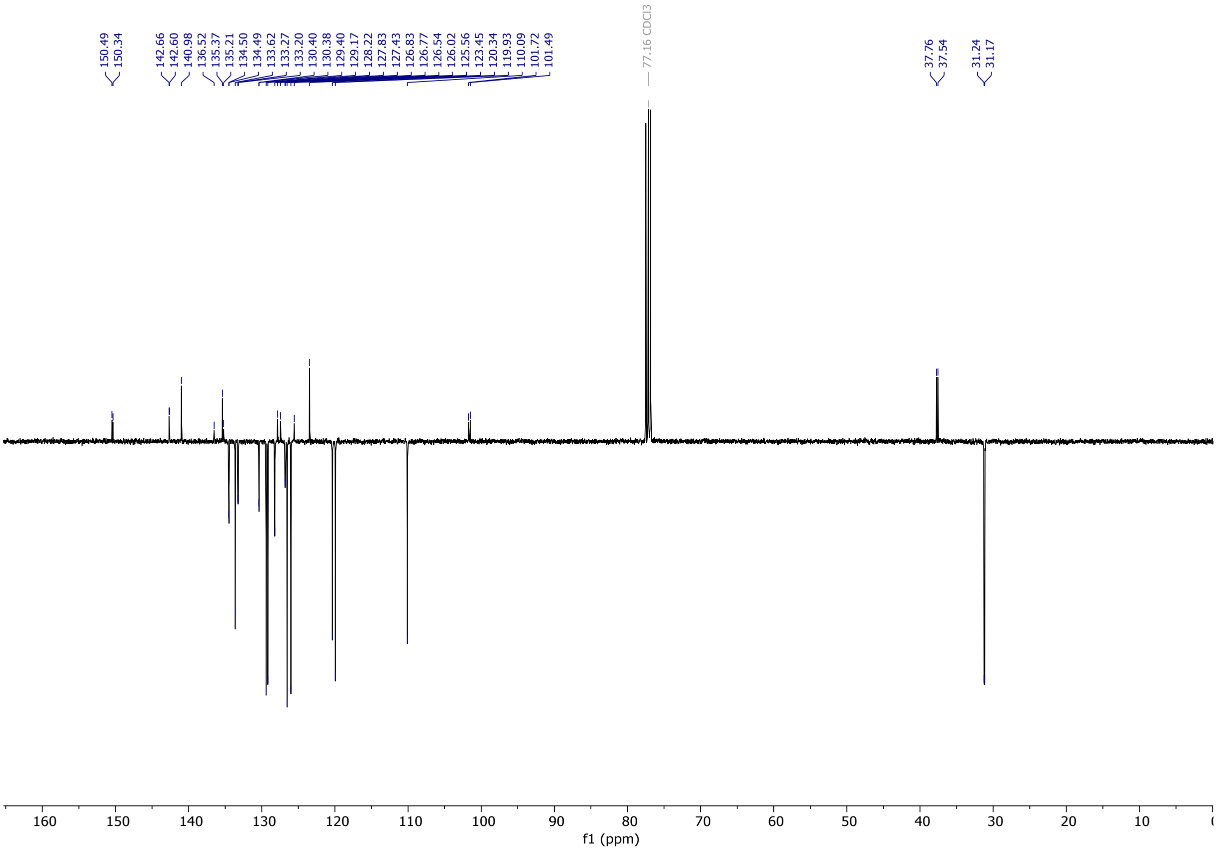

Figure S9. ^{31}P NMR (162 MHz, $CDCl_3$) spectrum of gold complex **3**

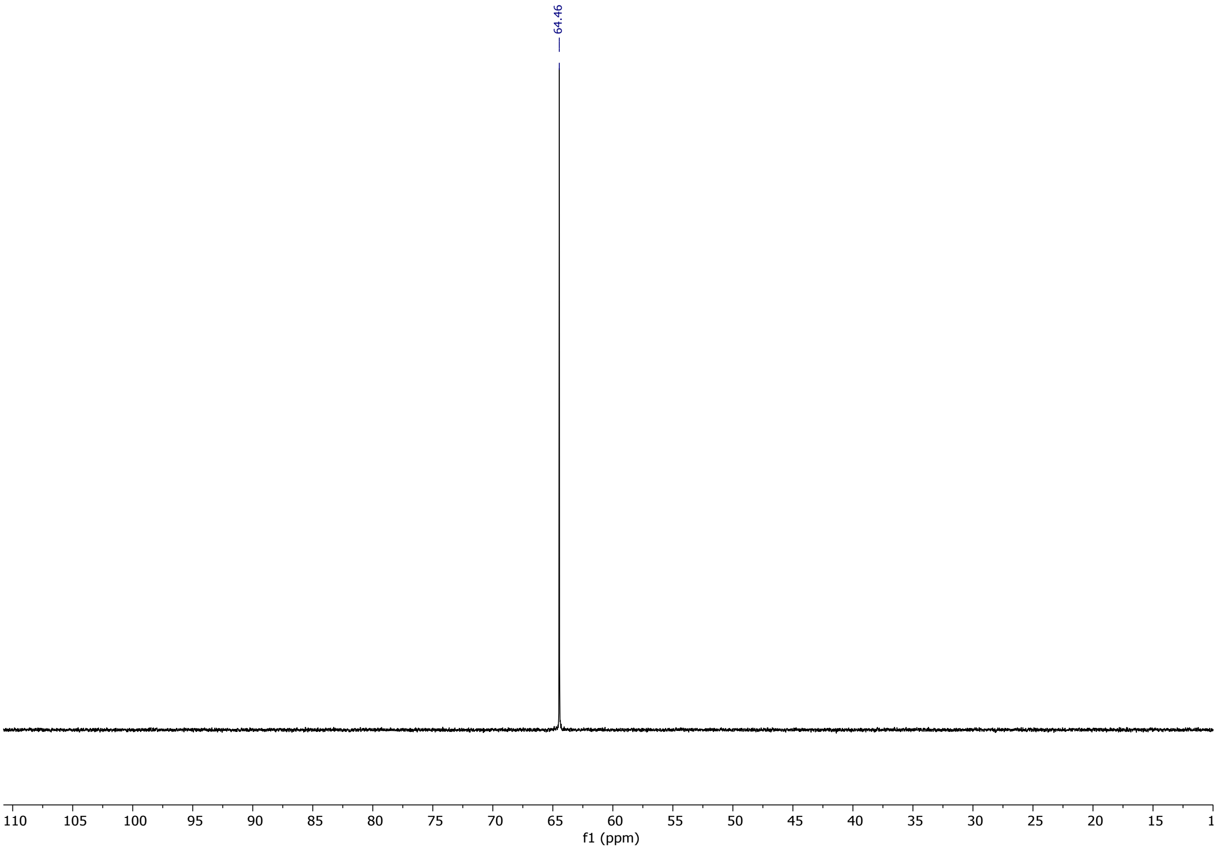

Figure S10. ^{1}H NMR (400 MHz, $CDCl_3$) spectrum of gold complex **4**

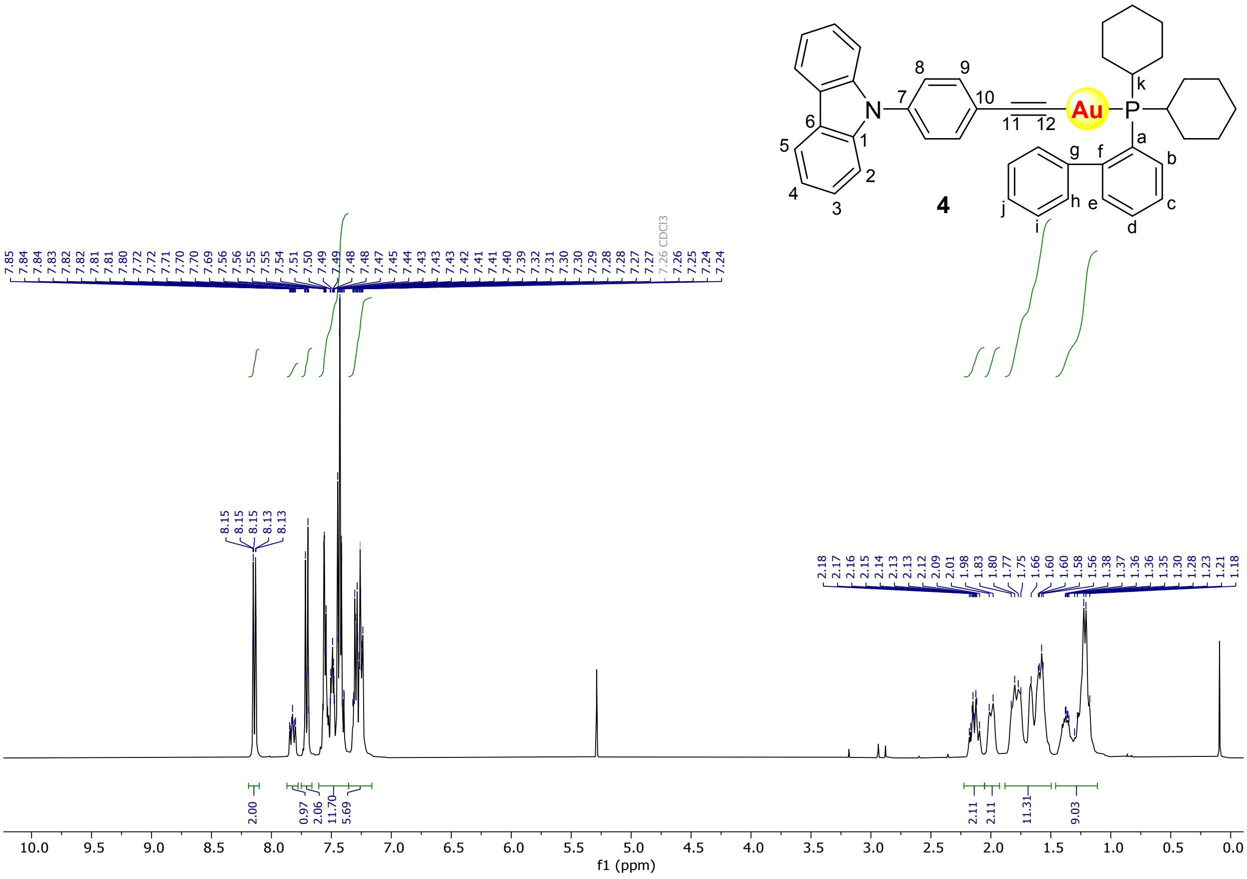

Figure S11. $^{13}C\{^1H\}$-APT NMR (101 MHz, $CDCl_3$) spectrum of gold complex **4**

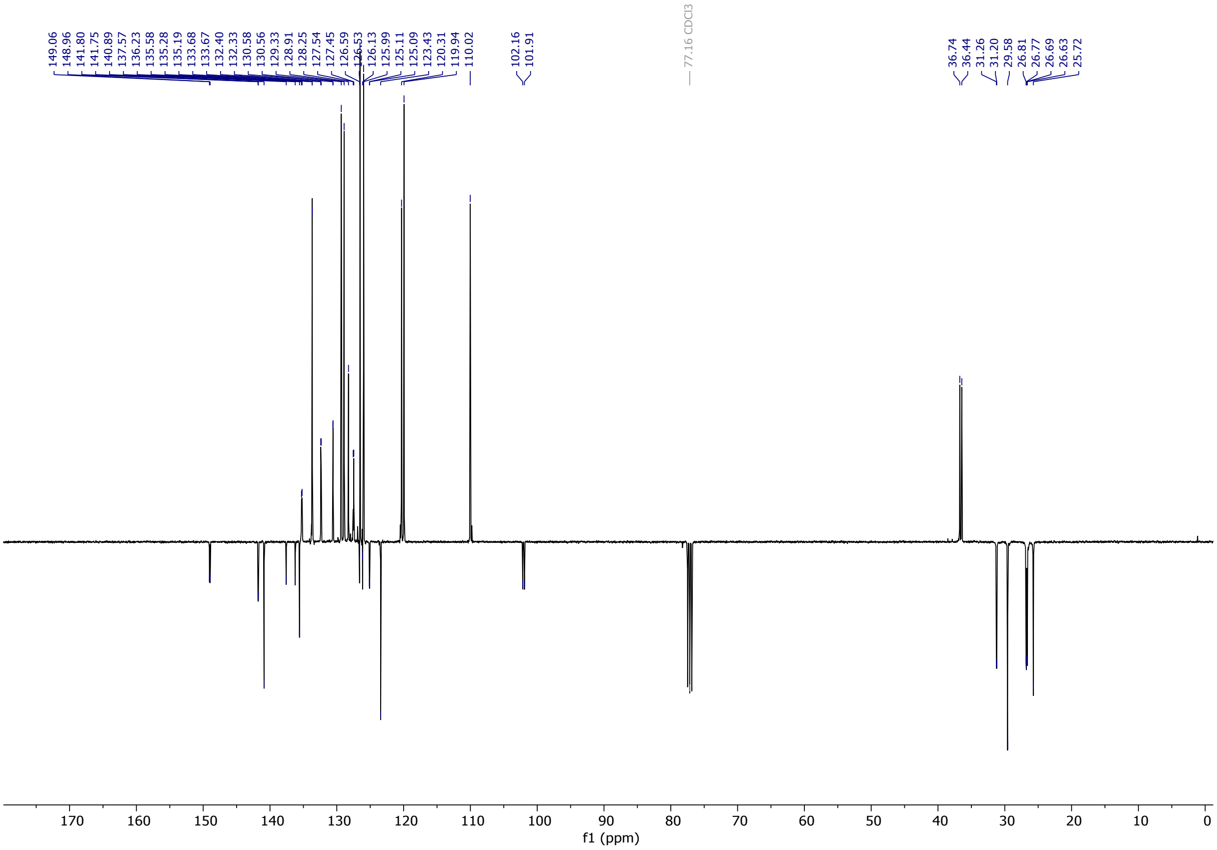

Figure S12. ^{31}P NMR (121 MHz, $CDCl_3$) spectrum of gold complex **4**

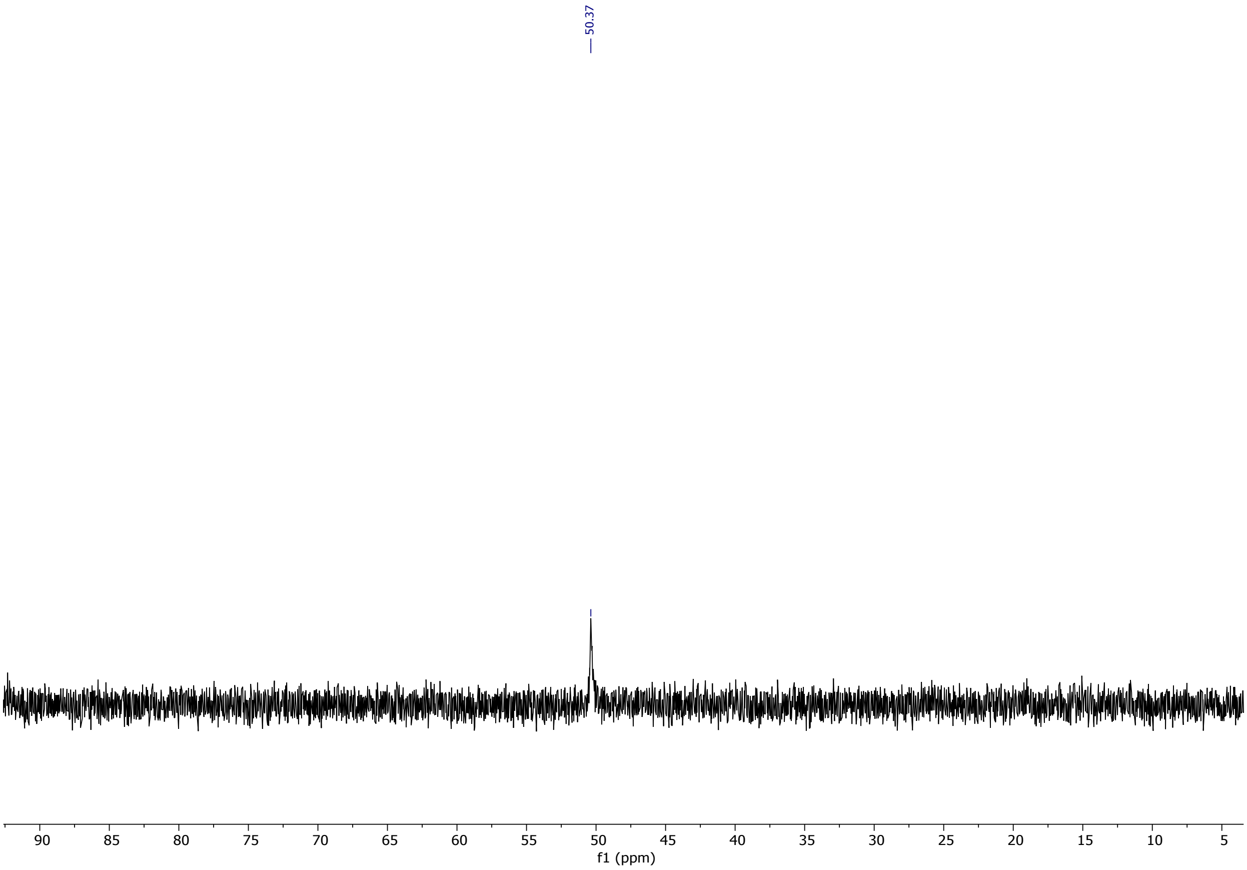

Figure S13. ^{1}H NMR (400 MHz, $CDCl_3$) spectrum of gold complex **5**

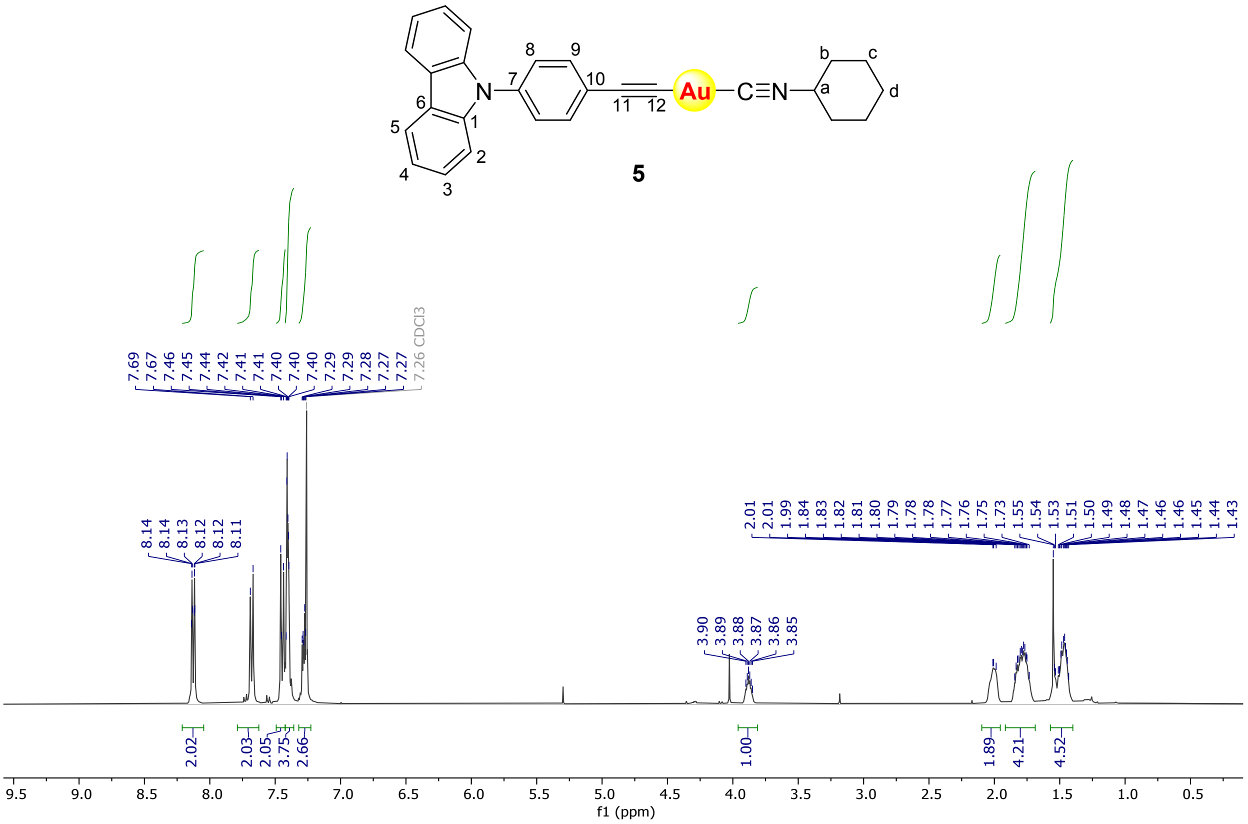

Figure S14. ^{13}C{^{1}H}-APT NMR (101 MHz, $CDCl_3$) spectrum of gold complex **5**

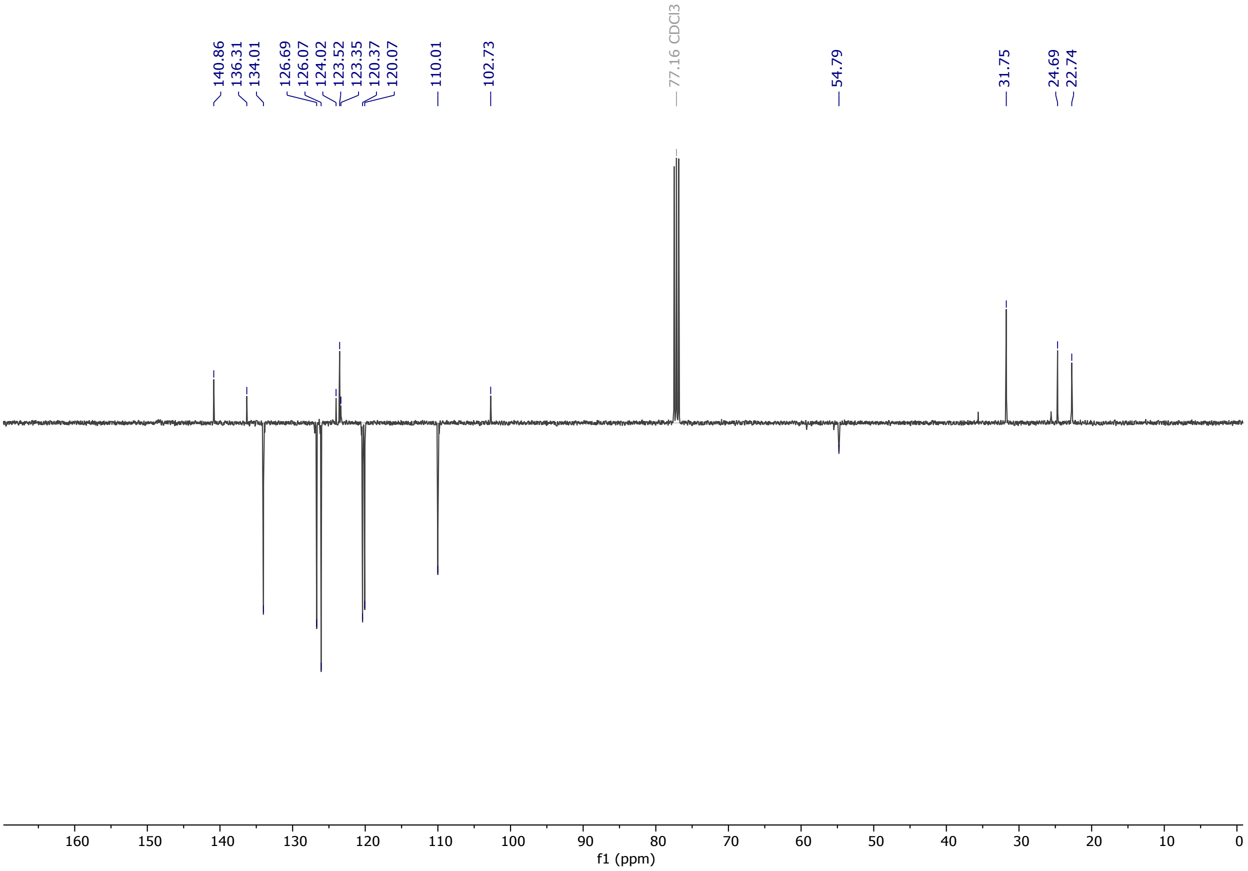

Figure S15. ^{1}H NMR (300 MHz, $CDCl_3$) spectrum of gold complex **6**

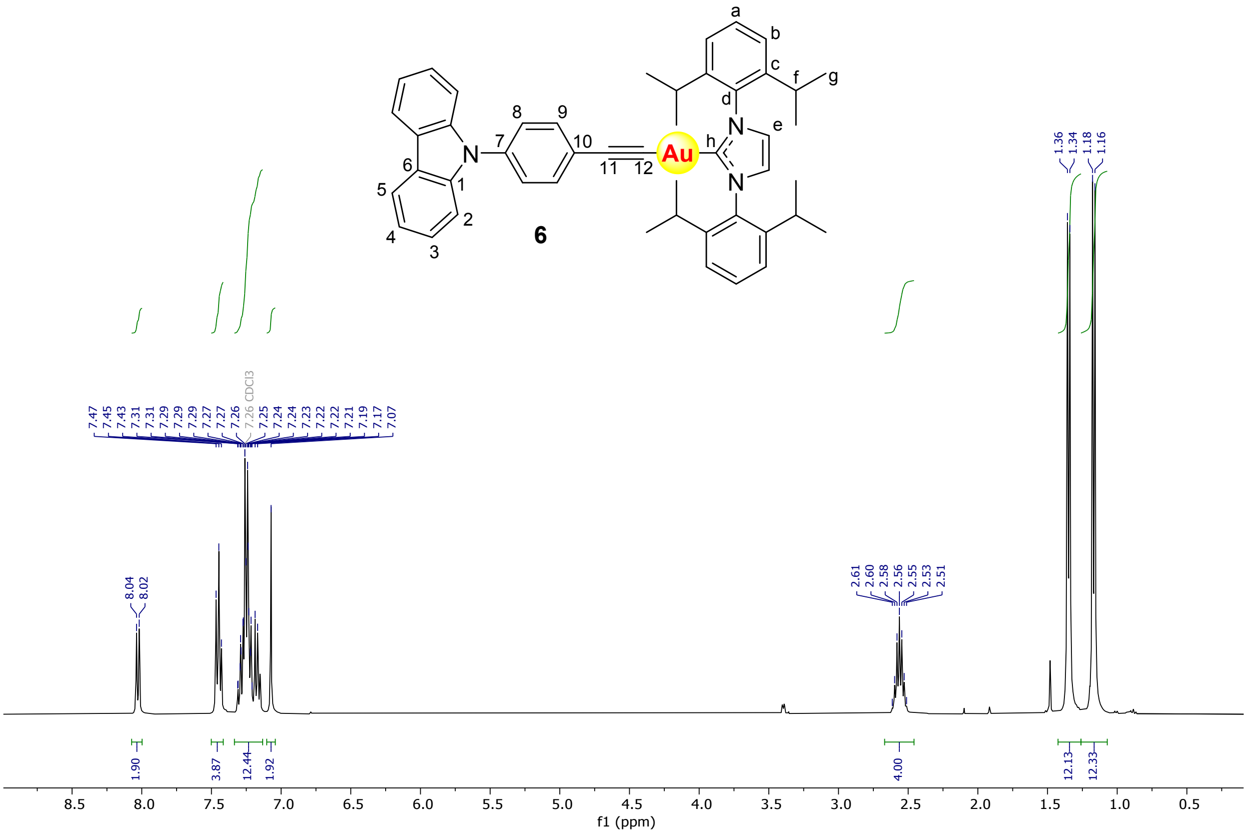

Figure S16. $^{13}C\{^{1}H\}$-APT NMR (101 MHz, $CDCl_3$) spectrum of gold complex **6**

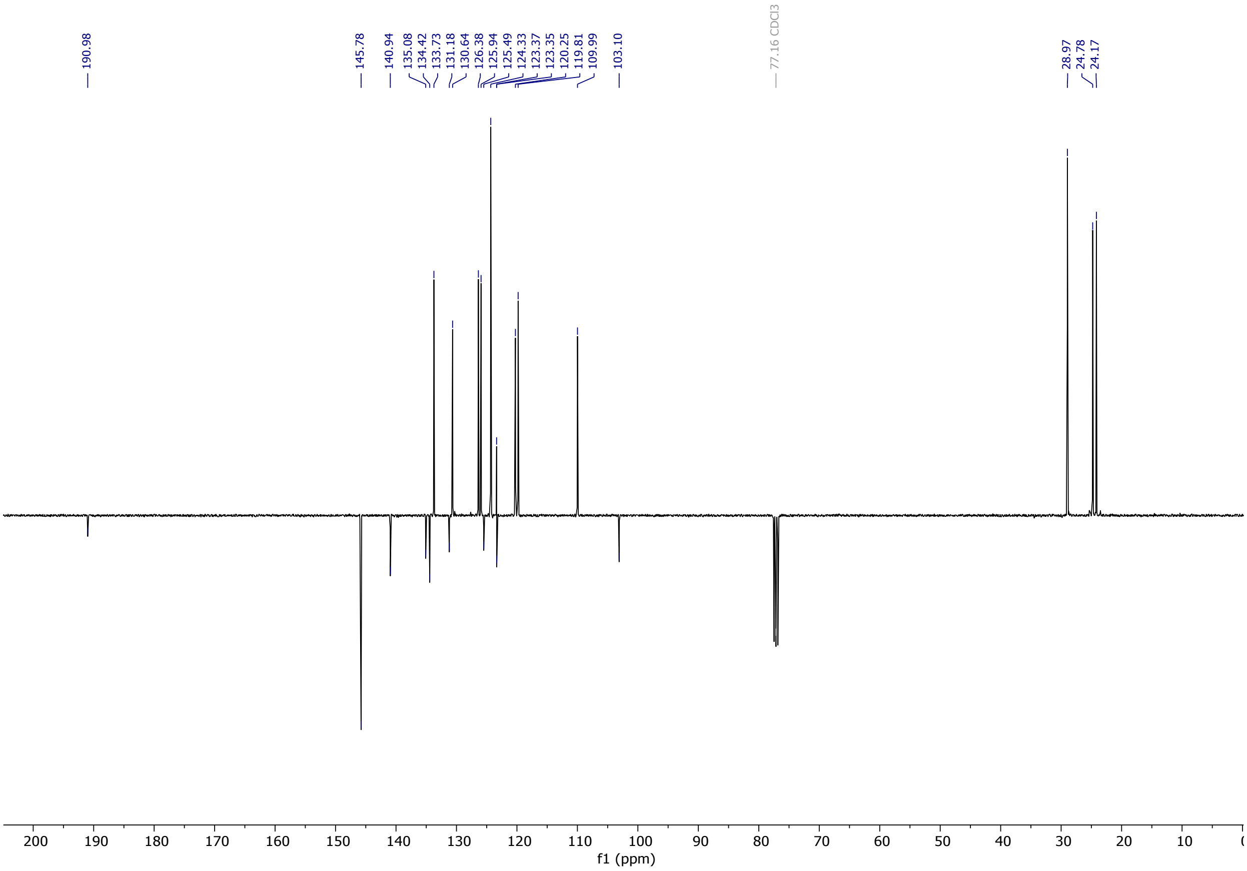

Figure S17. ^{1}H NMR (400 MHz, CD_2Cl_2) spectrum of gold complex **7**

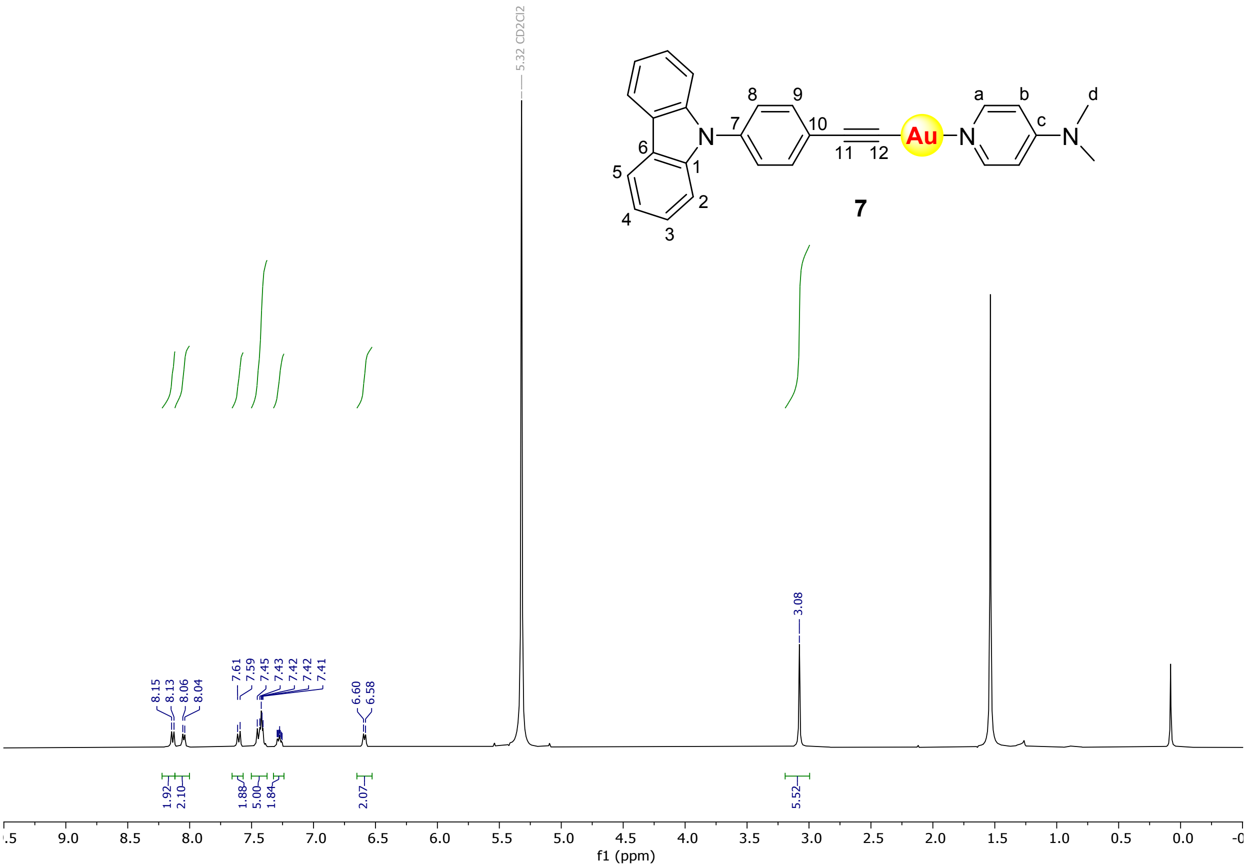

Figure S18. $^{13}C\{^{1}H\}$-APT NMR (101 MHz, $CDCl_3$) spectrum of gold complex **7**

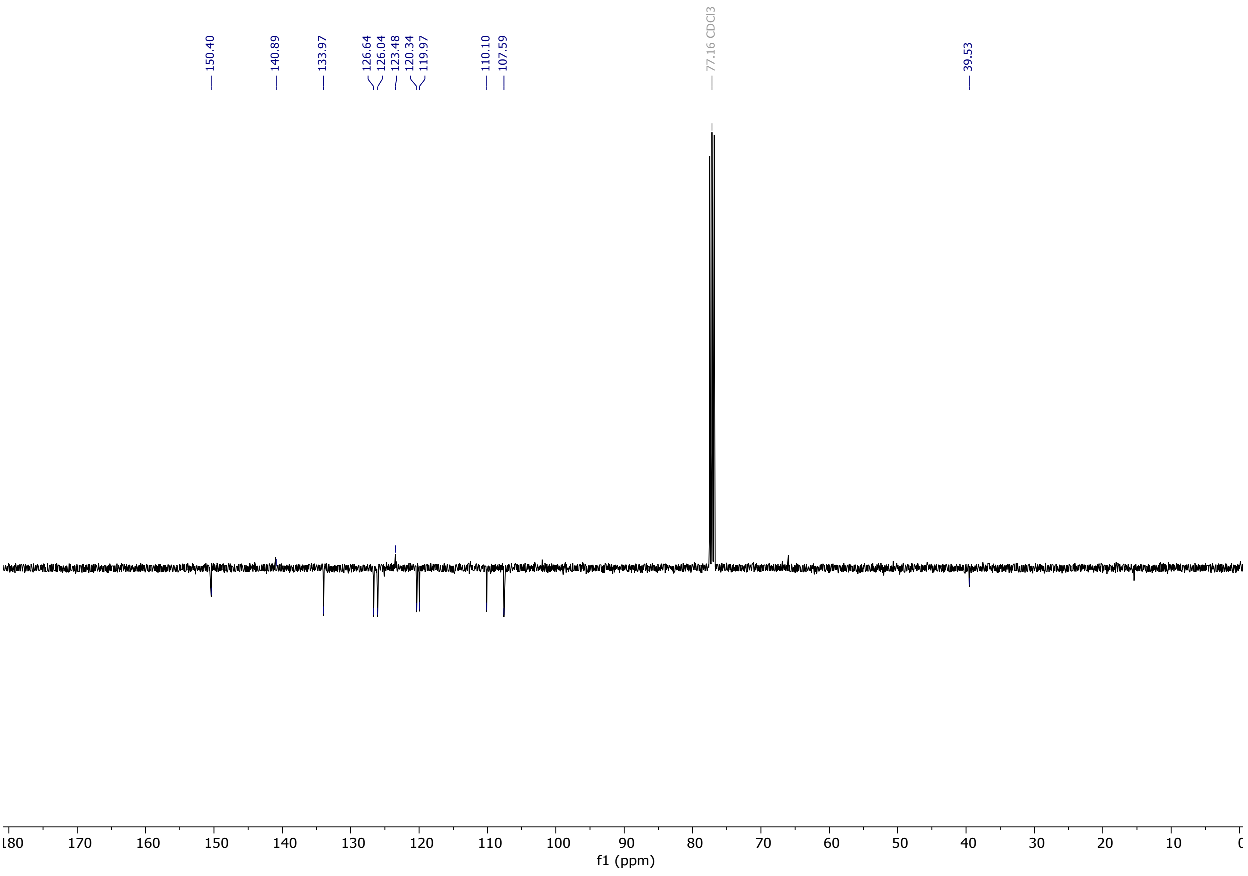

Figure S19. ^{1}H NMR (300 MHz, $CDCl_3$) spectrum of gold complex **8**

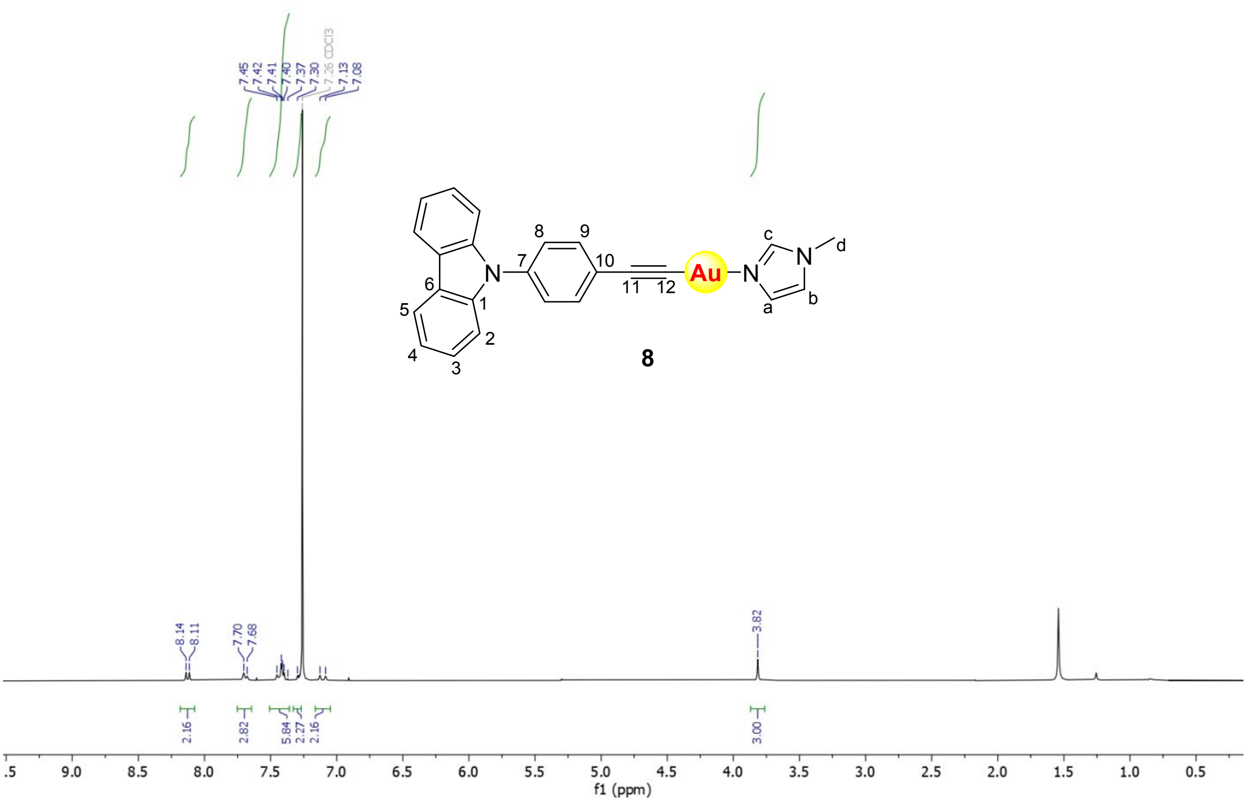

Figure S20. ^{1}H NMR (400 MHz, $CDCl_3$) spectrum of gold complex **9**

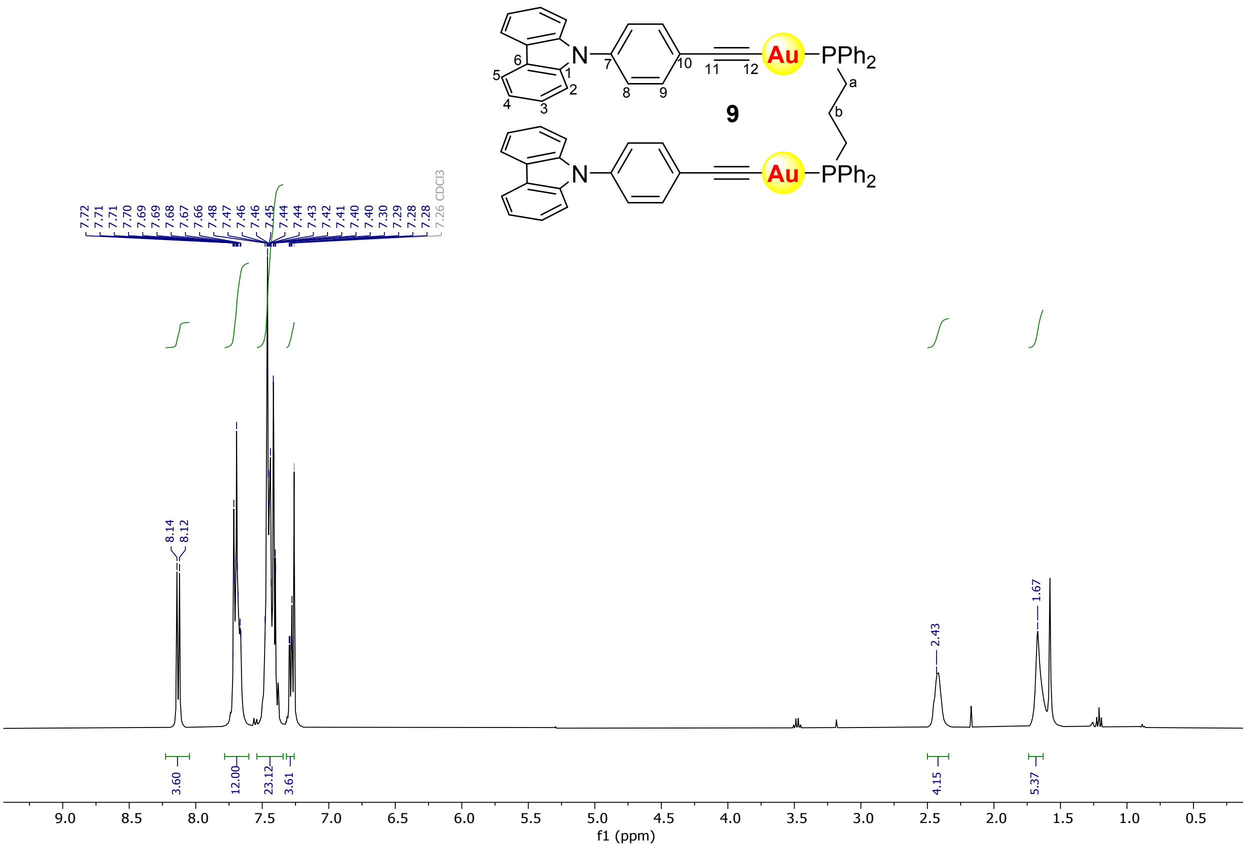

Figure S21. $^{13}C\{^{1}H\}$-APT NMR (101 MHz, $CDCl_3$) spectrum of gold complex **9**

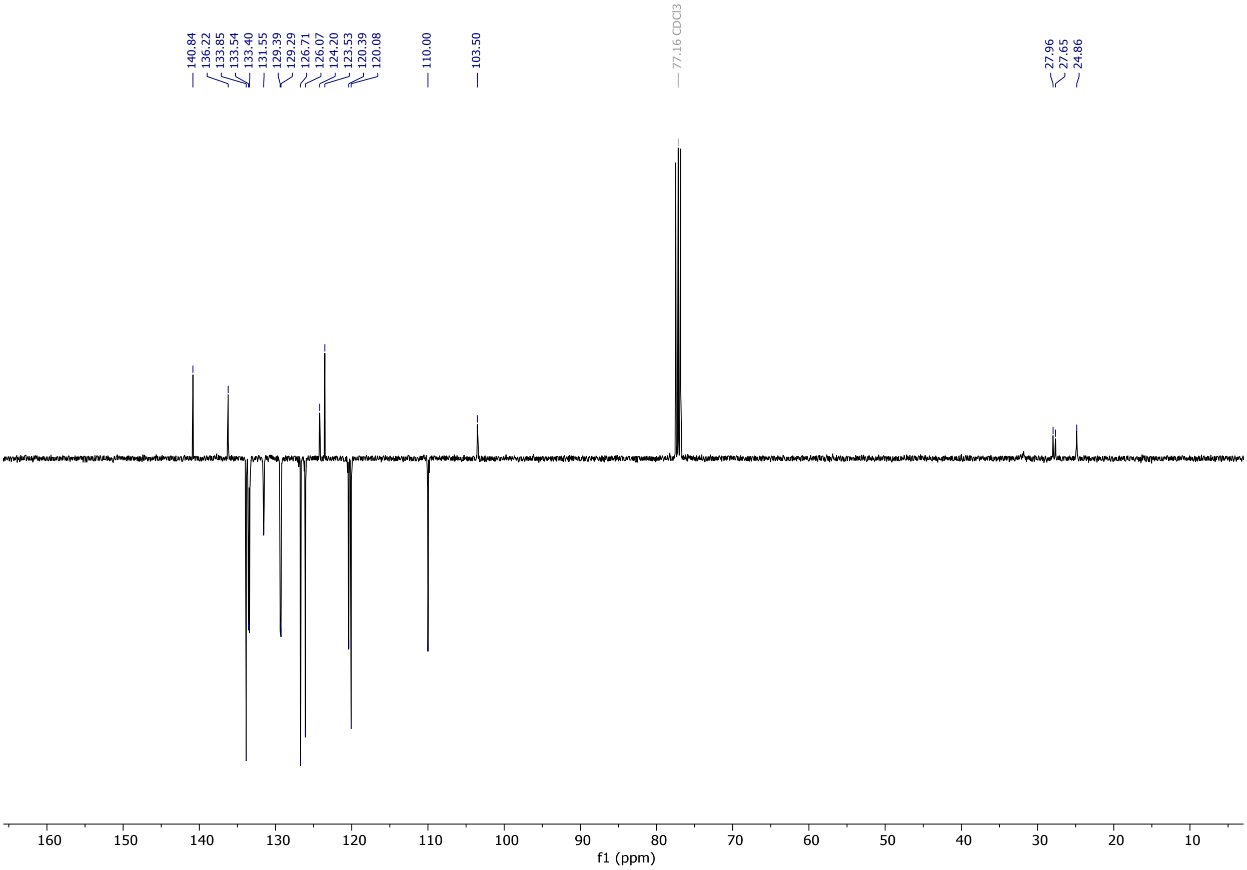

Figure S22. ^{31}P NMR (162 MHz, $CDCl_3$) spectrum of gold complex **9**

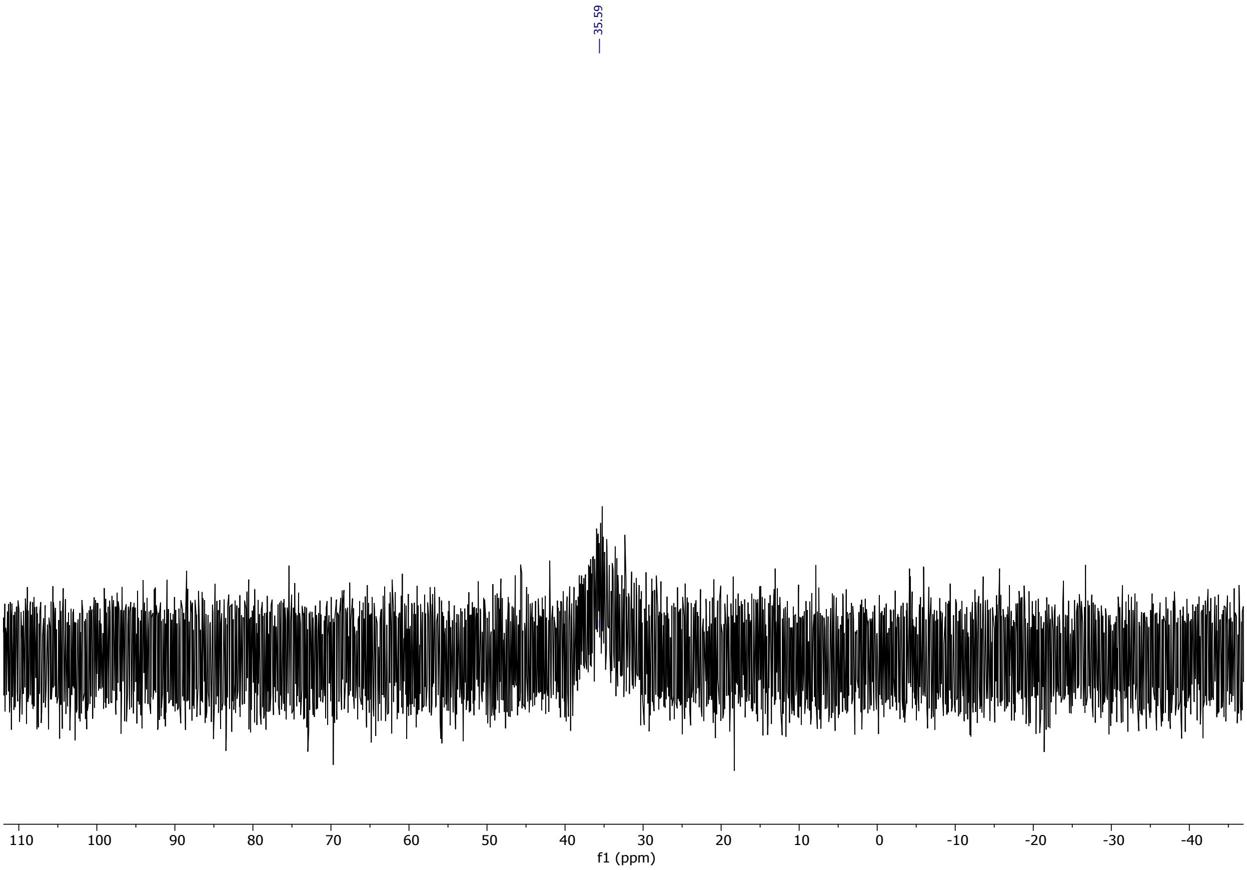

Figure S23. ^{1}H NMR (400 MHz, toluene-d_8) spectrum of gold complex **10**

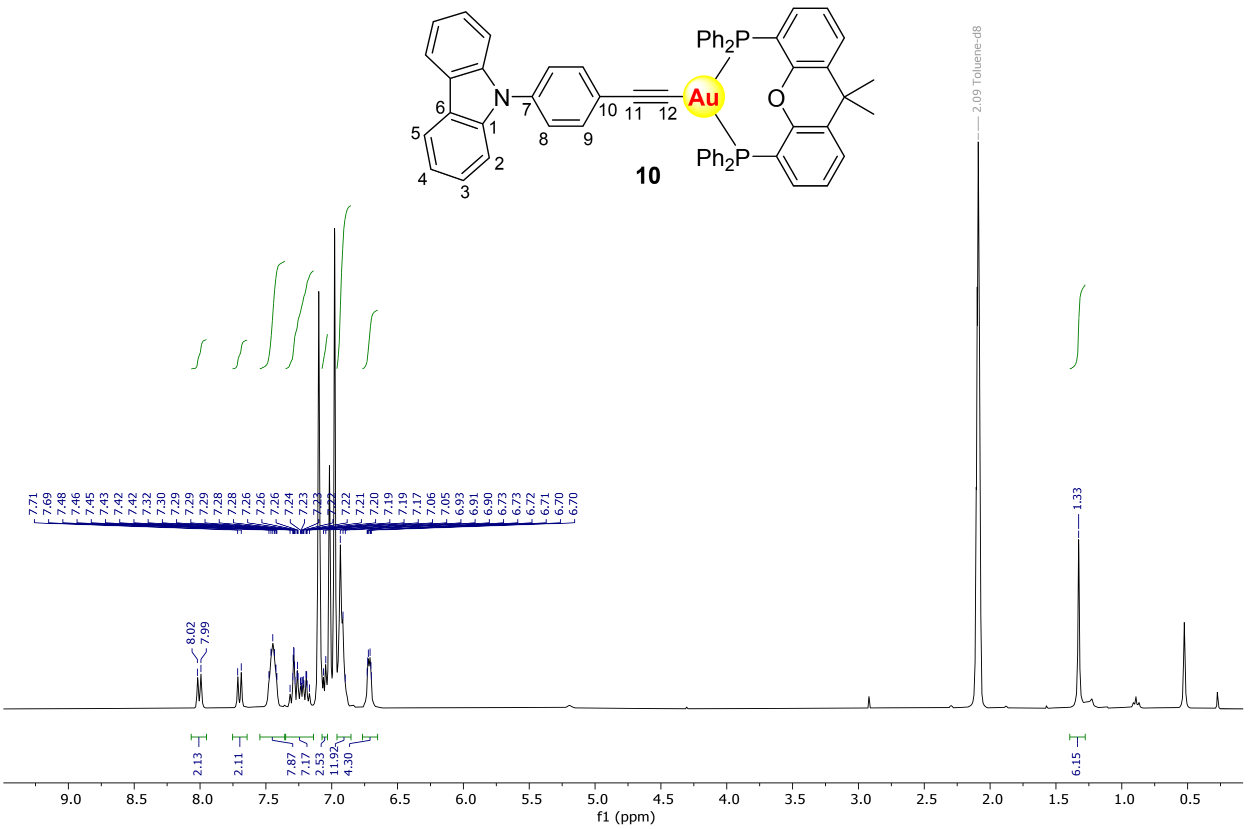

Figure S24. ^{13}C{^{1}H}-APT NMR (101 MHz, toluene-d_8) spectrum of gold complex **10**

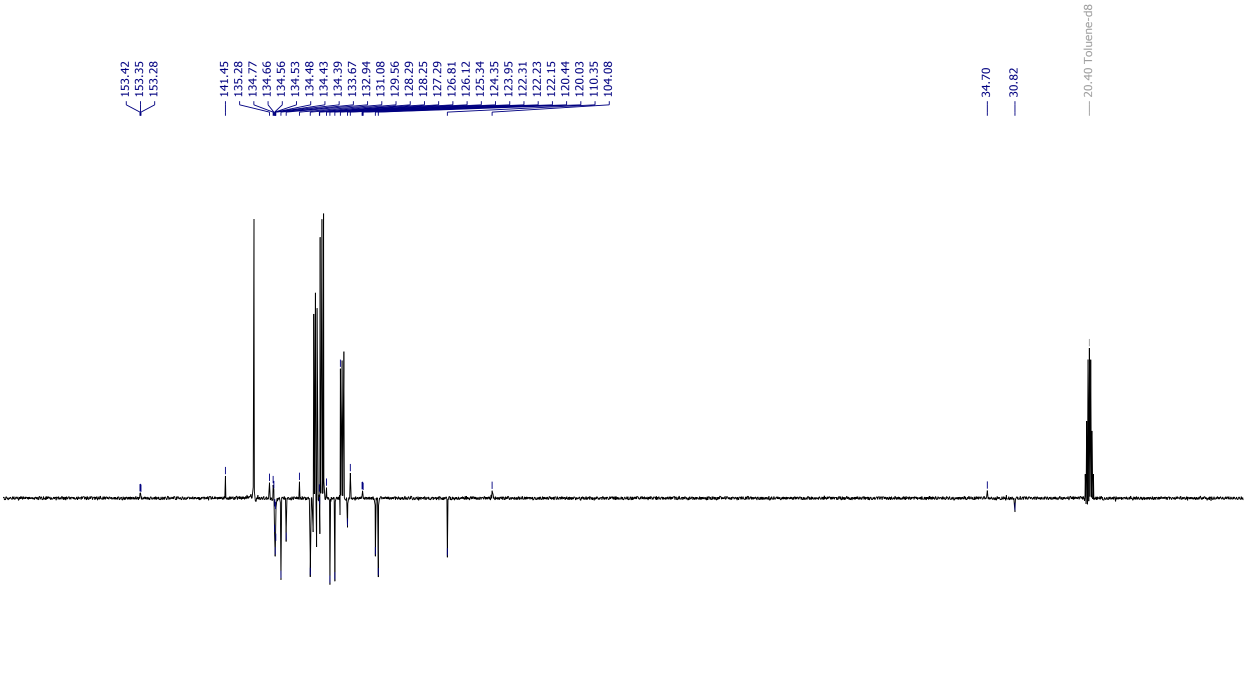

Figure S25. ^{31}P NMR (162 MHz, $CDCl_3$) spectrum of gold complex **10**

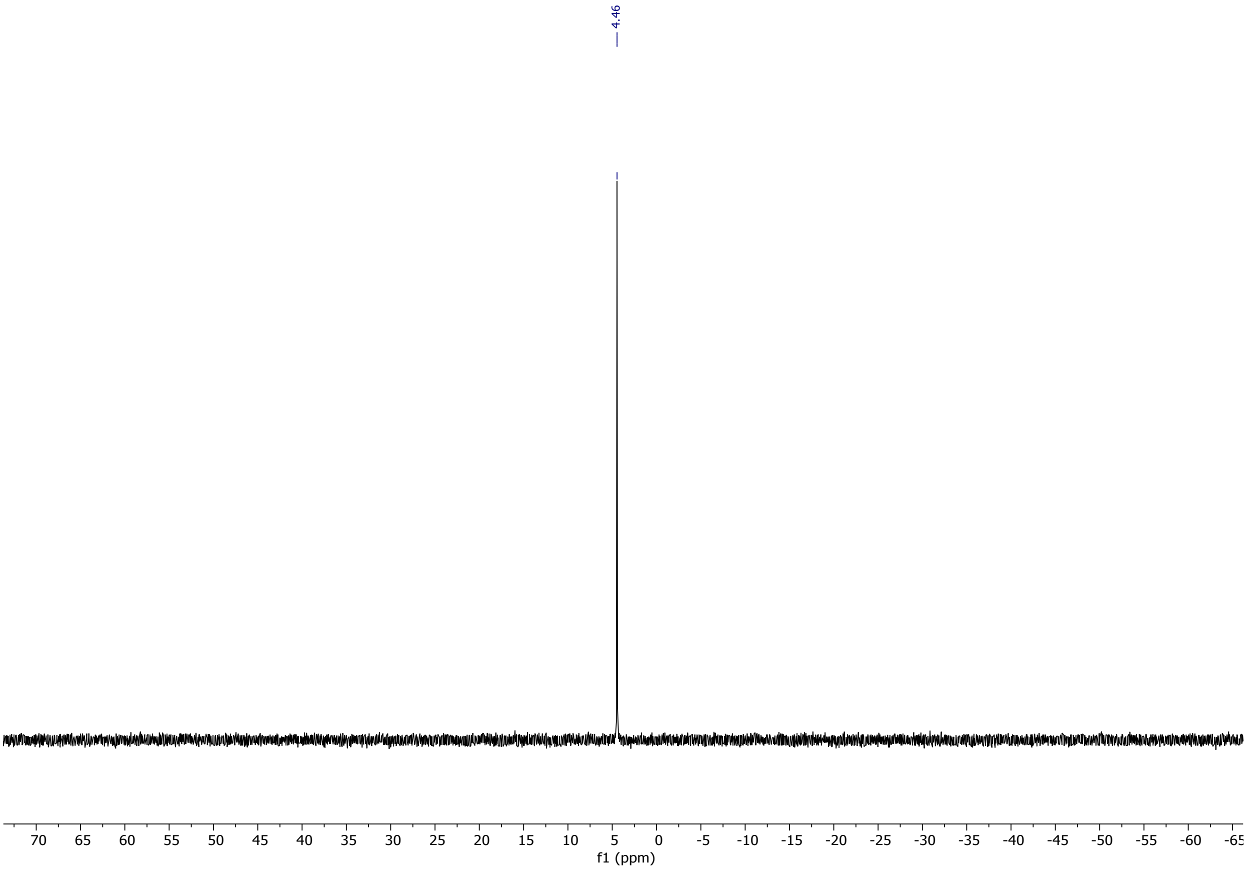

Figure S26. ^{1}H NMR (400 MHz, CD_2Cl_2) spectrum of gold complex **12**

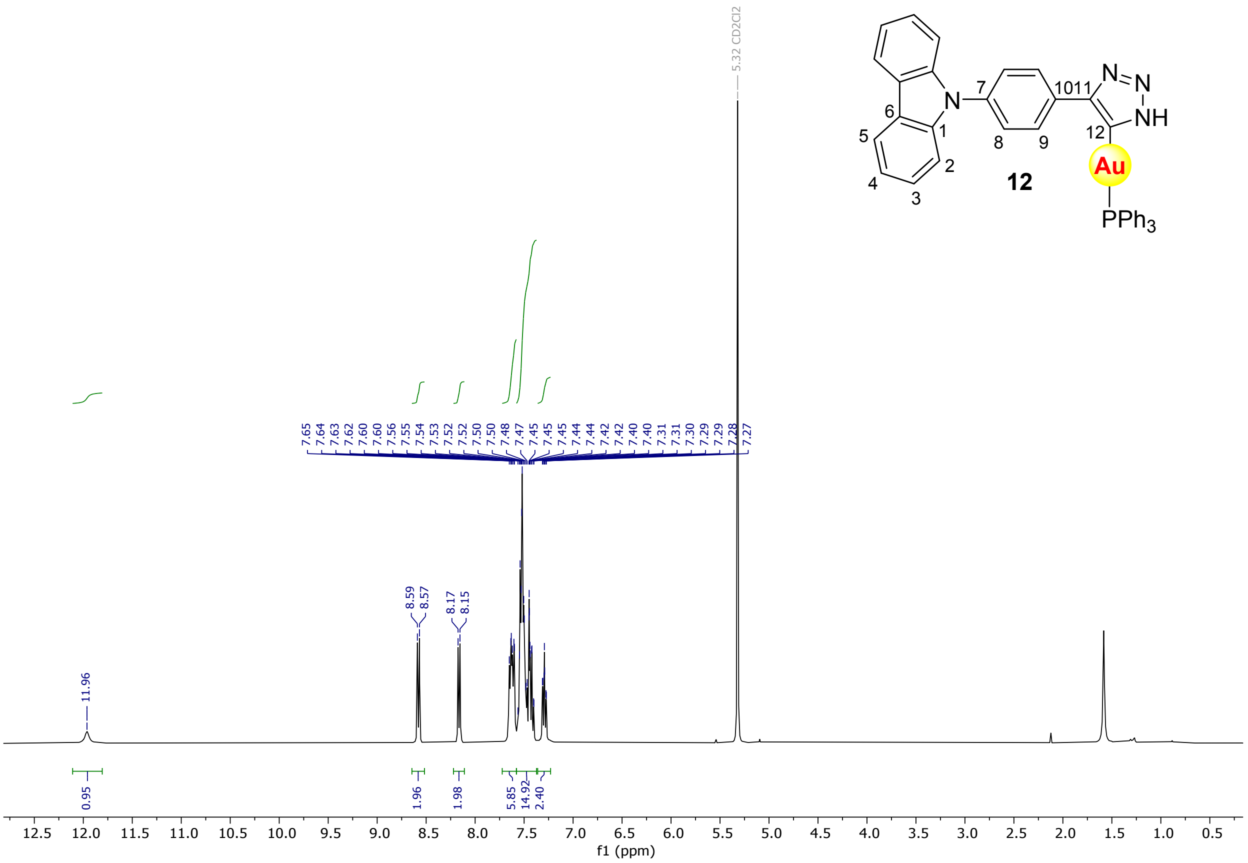

Figure S27. $^{13}C\{^{1}H\}$-APT NMR (101 MHz, CD_2Cl_2) spectrum of gold complex **12**

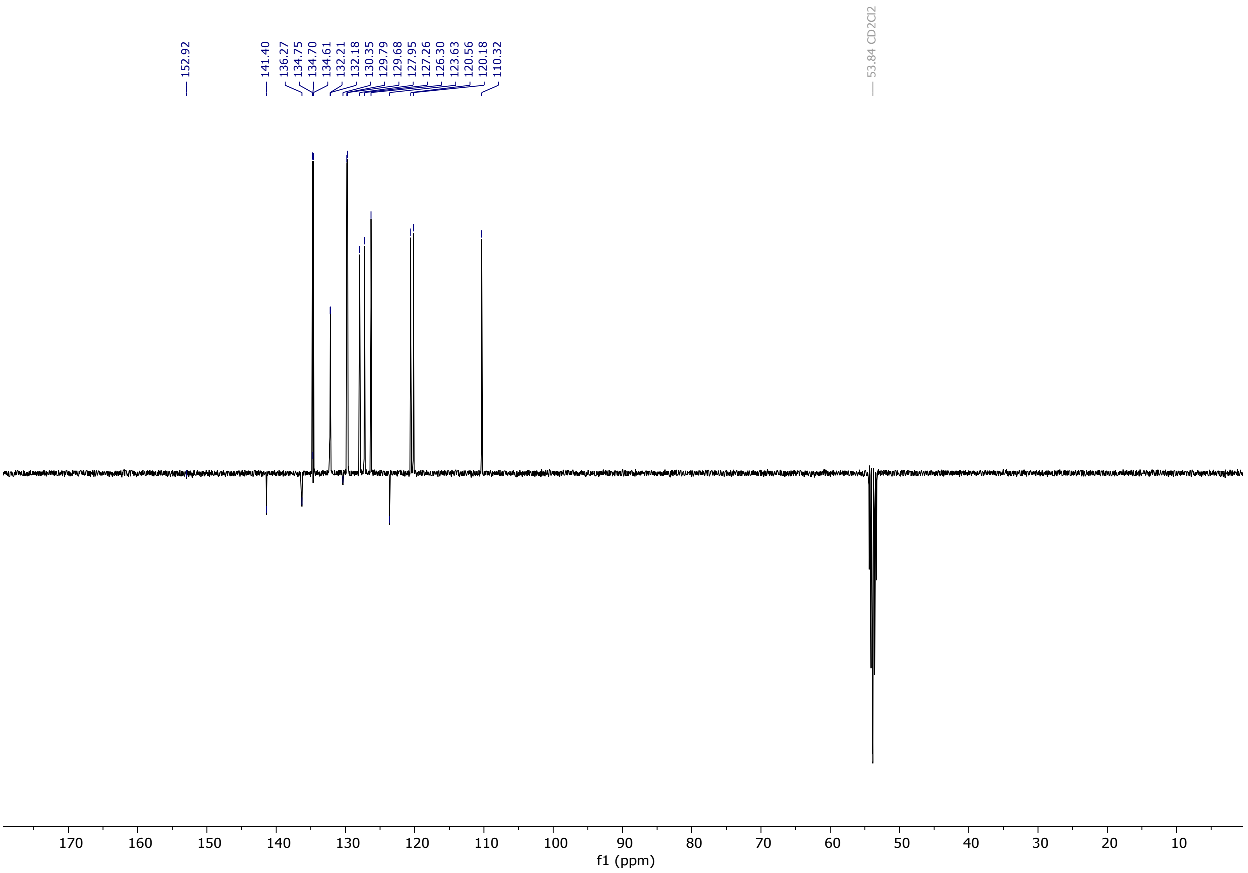

Figure S28. $^{31}P\{^{1}H\}$ NMR (162 MHz, CD_2Cl_2) spectrum of gold complex **12**

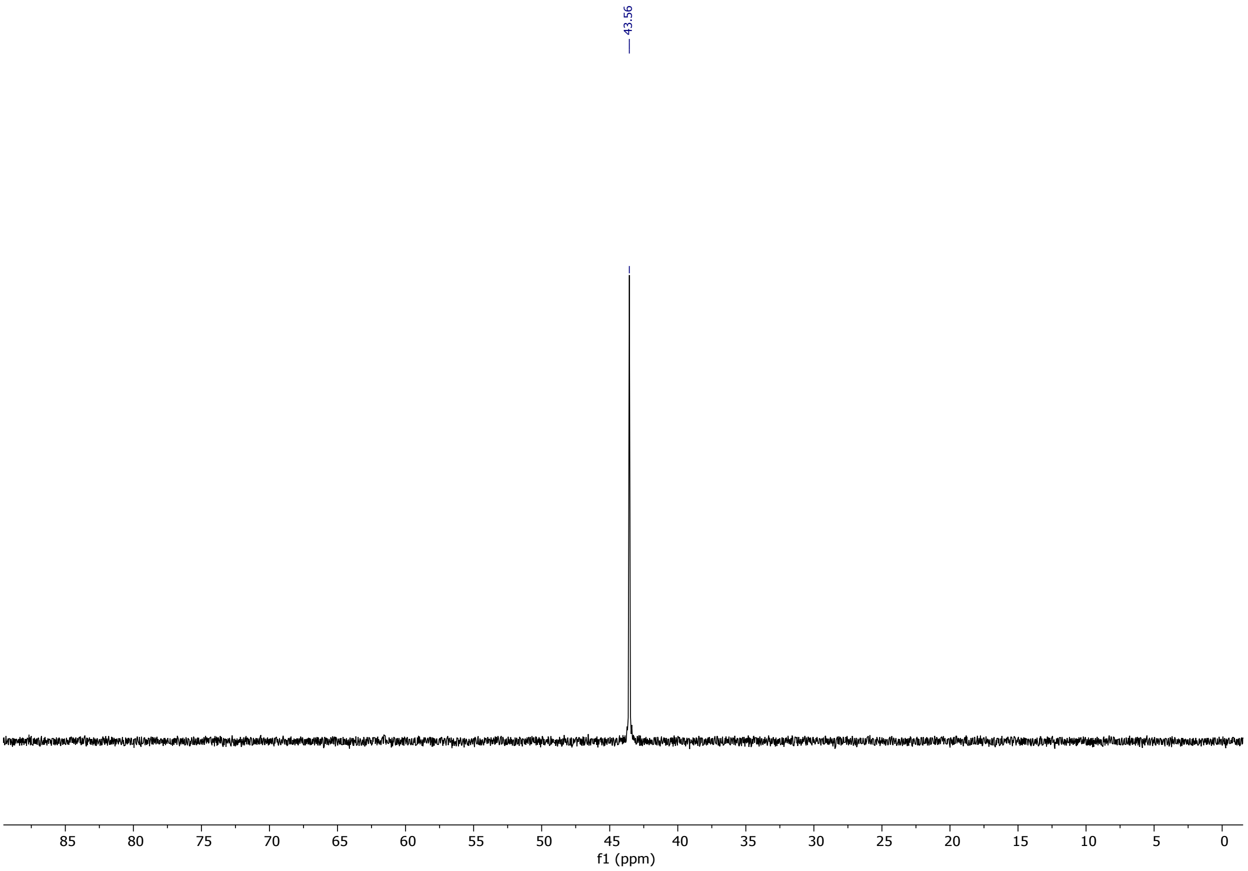

Figure S29. ^{1}H NMR (400 MHz, CD_2Cl_2) spectrum of gold complex **13**

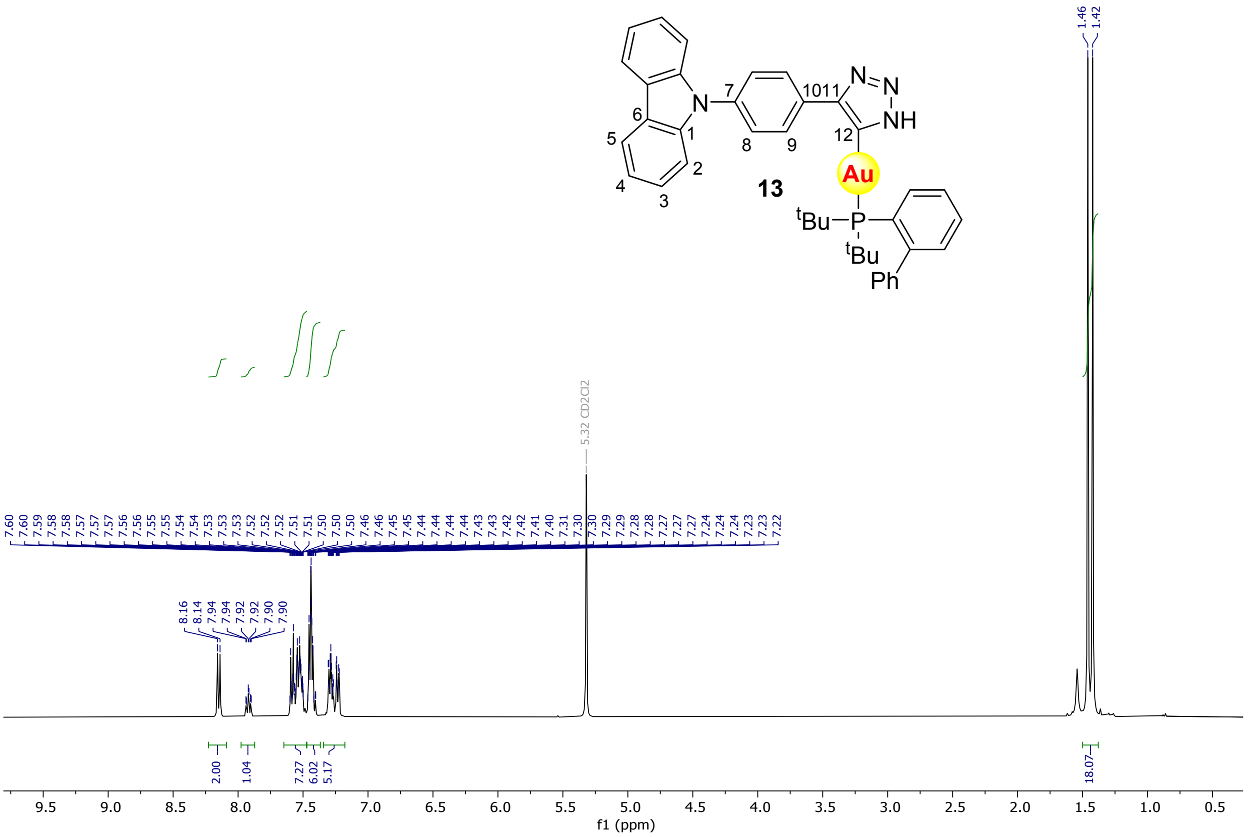

Figure S30. $^{13}C\{^{1}H\}$-APT NMR (101 MHz, CD_2Cl_2) spectrum of gold complex **13**

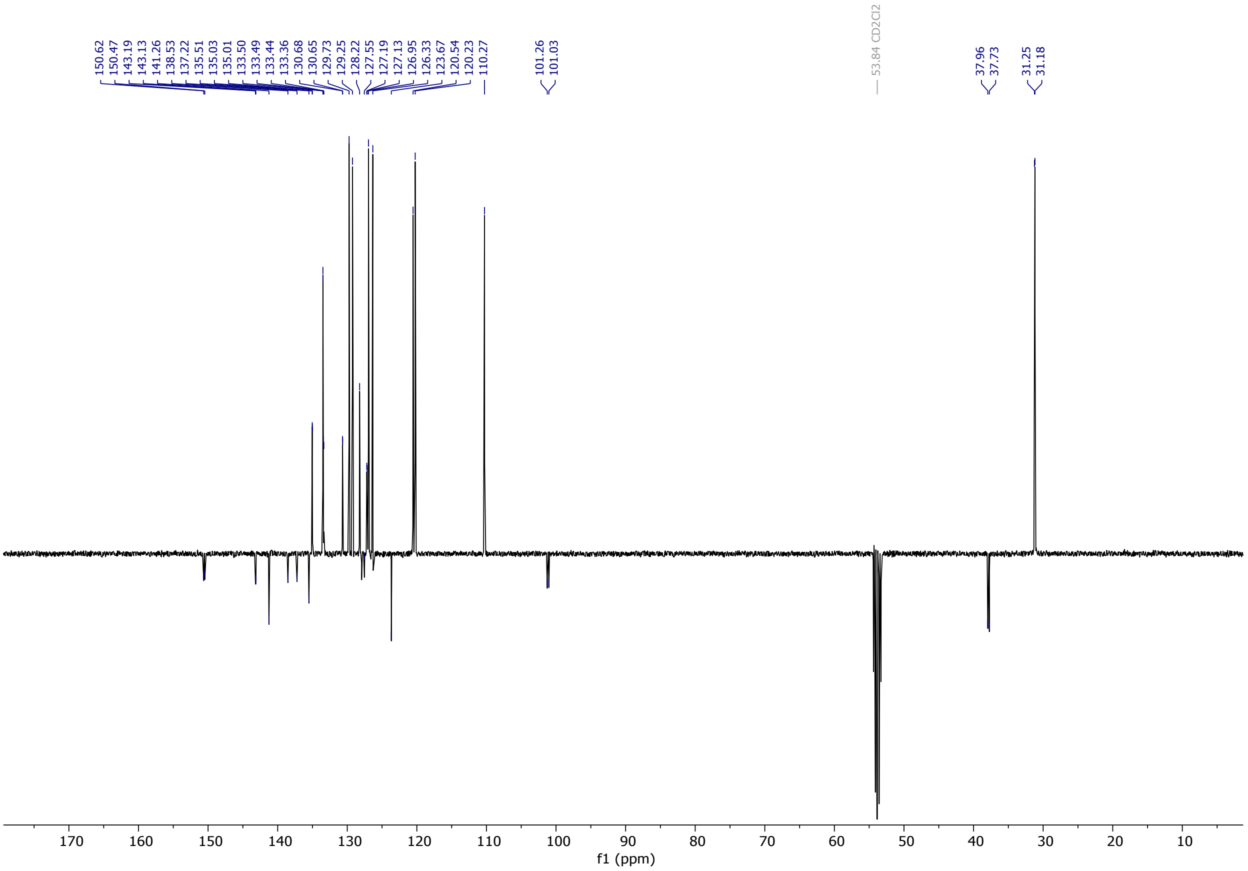

Figure S31. $^{31}P\{^{1}H\}$ NMR (162 MHz, CD_2Cl_2) spectrum of gold complex **13**

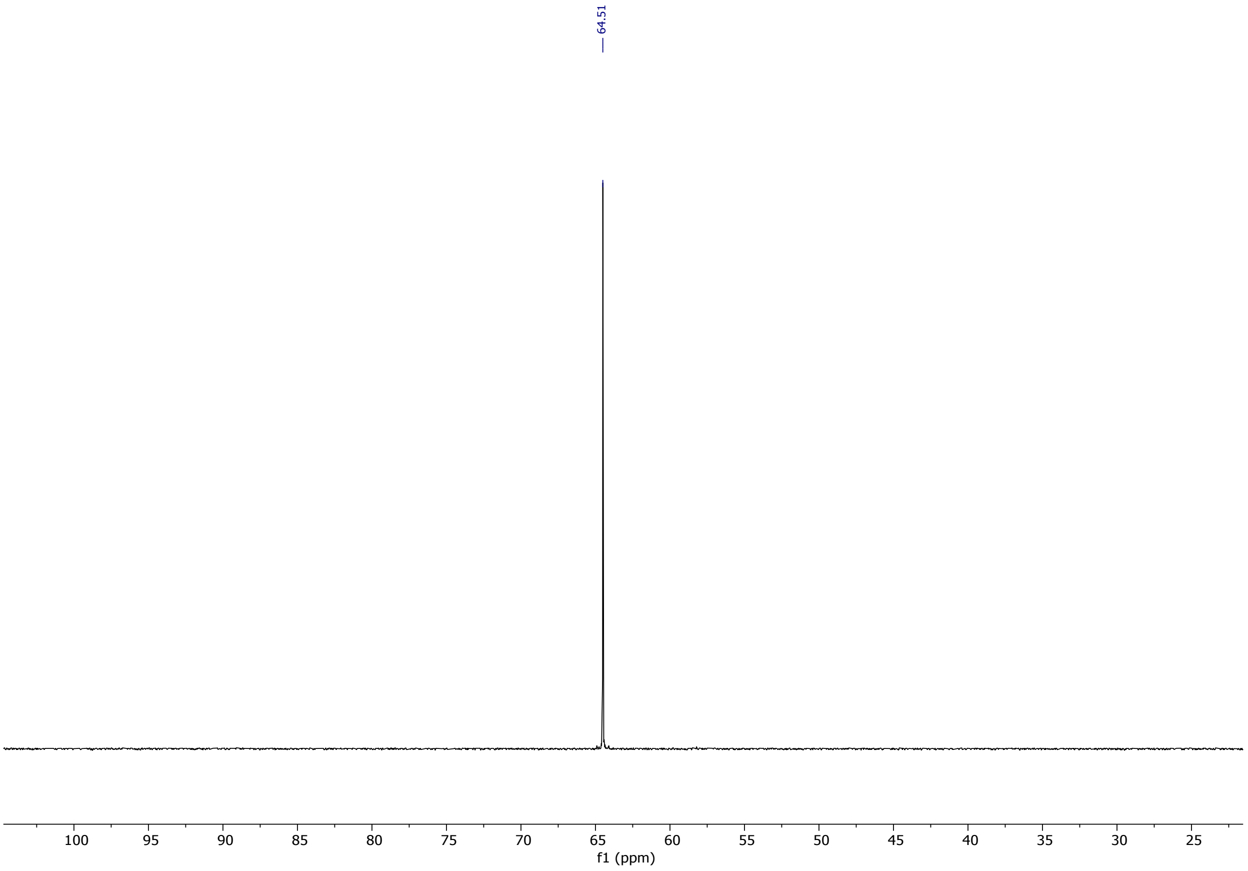

4. Temperature-dependent NMR study of complex 10

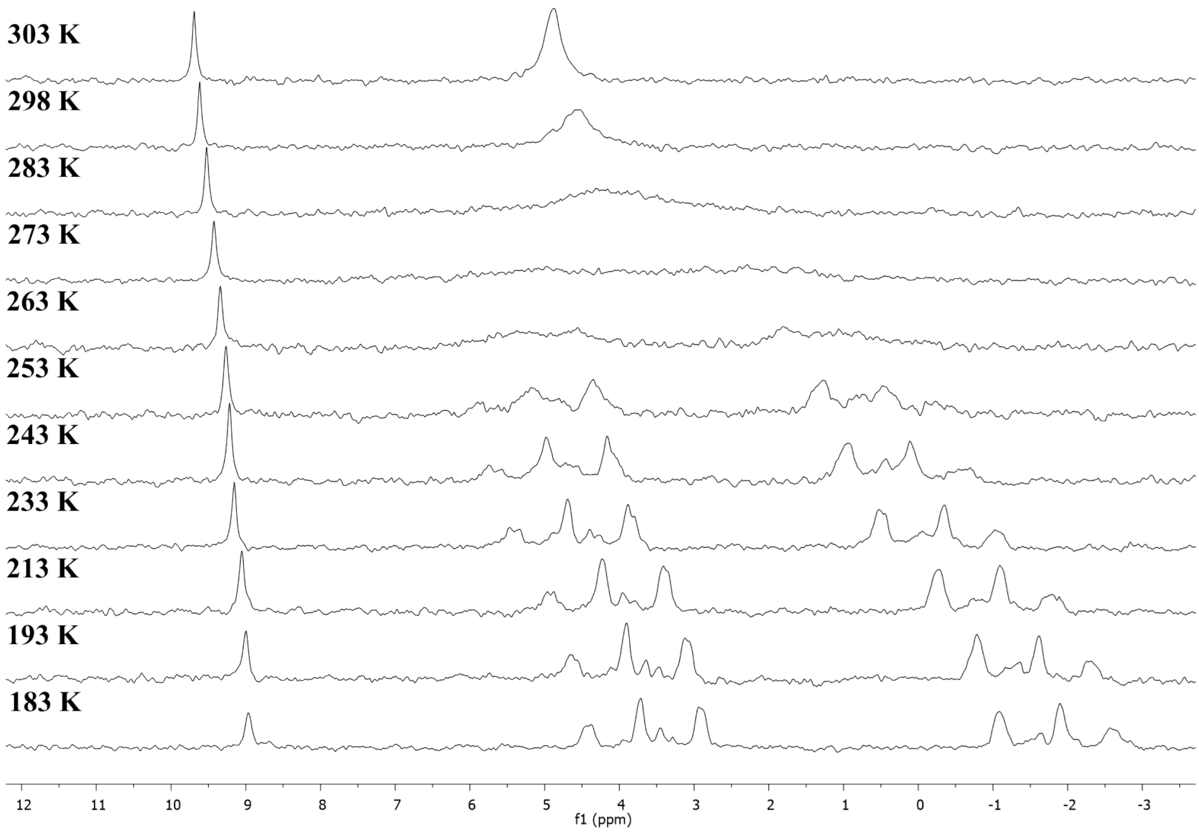

Figure S32. $^{31}P\{^1H\}$ NMR spectra stacking at different temperatures for complex **10**.

5. Absorption, excitation and emission spectra

Figure S33. Normalized excitation and emission bands of **EPC**

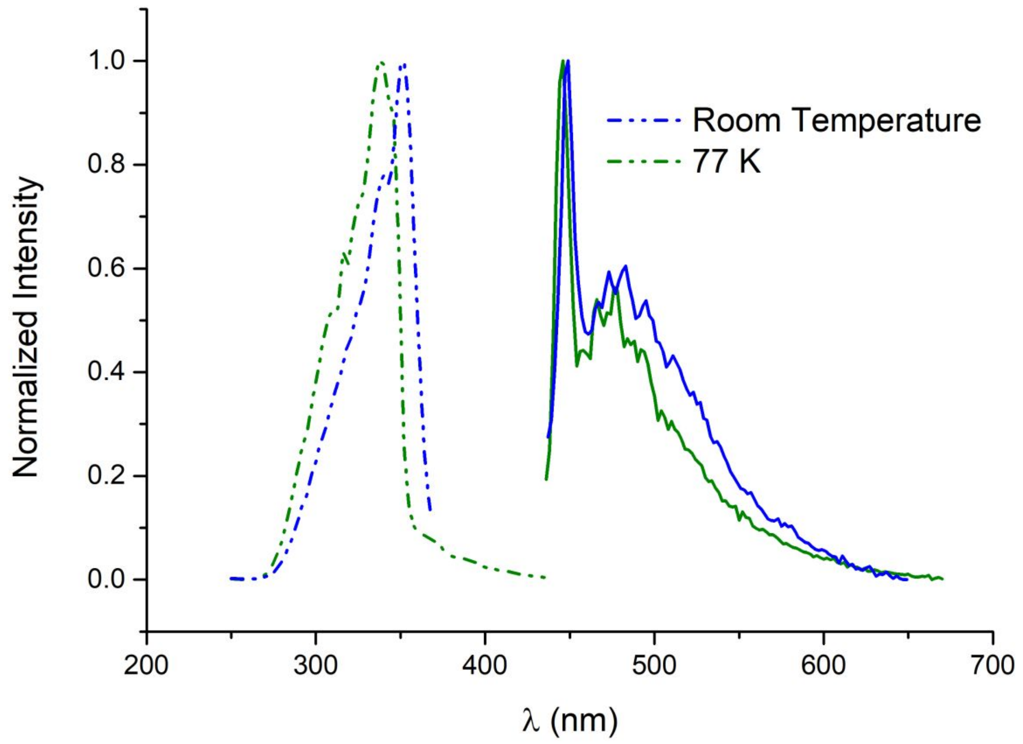

Figure S34. Absorption spectrum of **EPC**

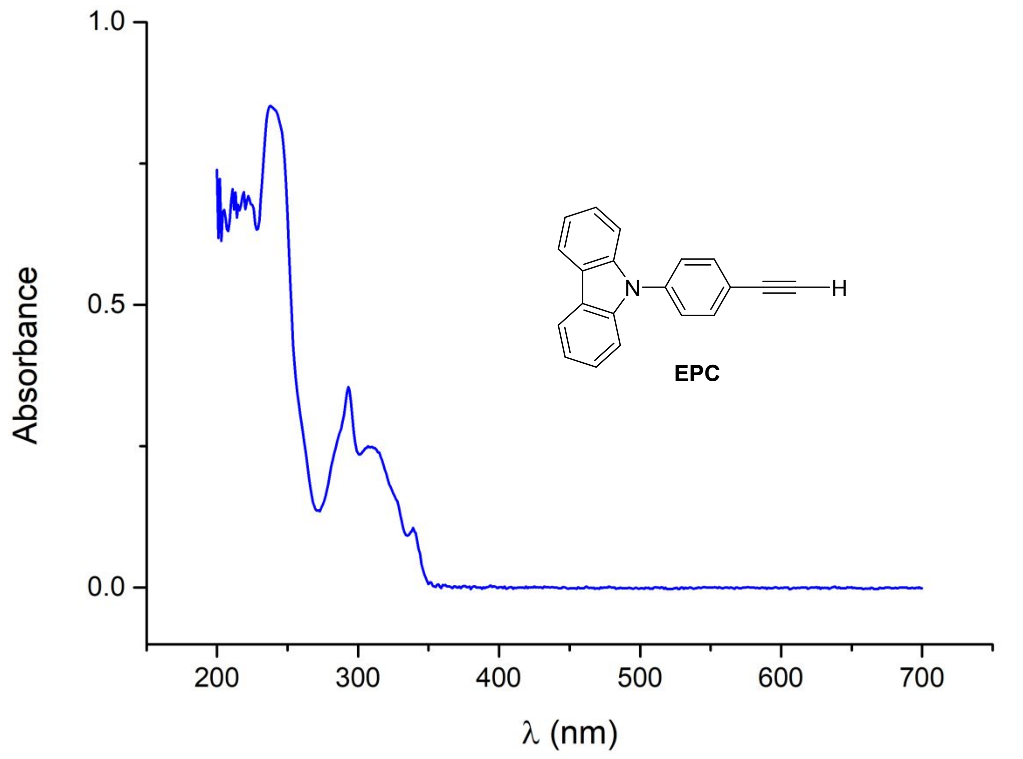

Figure S35. Normalized excitation and emission bands of gold complex **1**

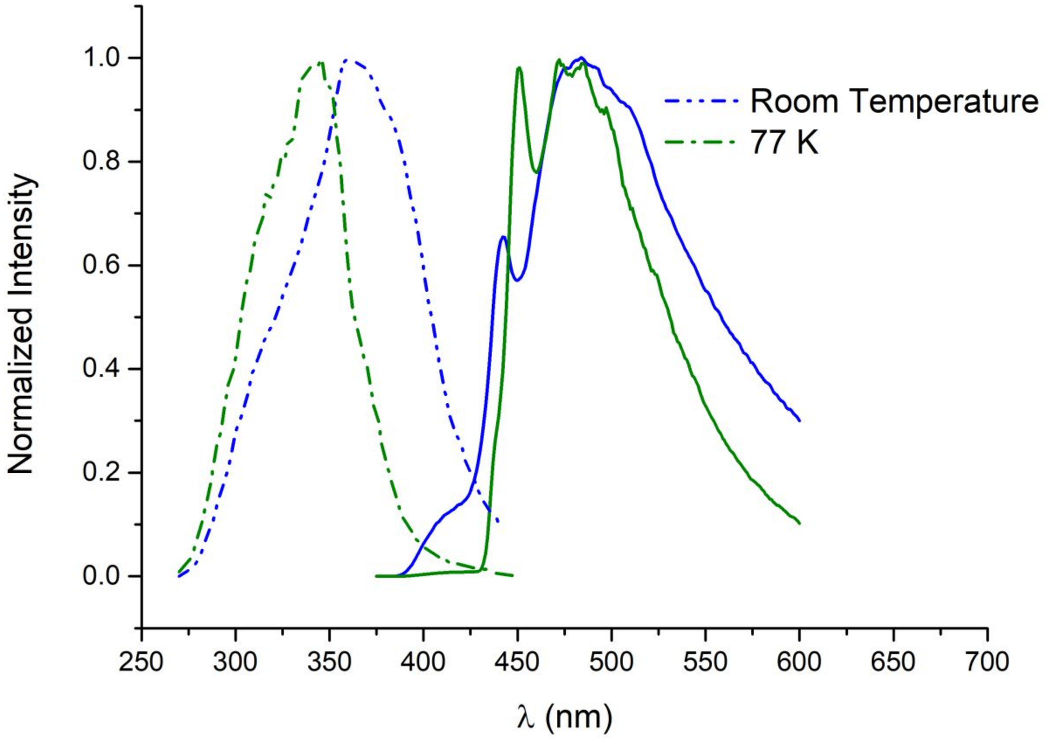

Figure S36. Absorption spectrum of gold complex **1**

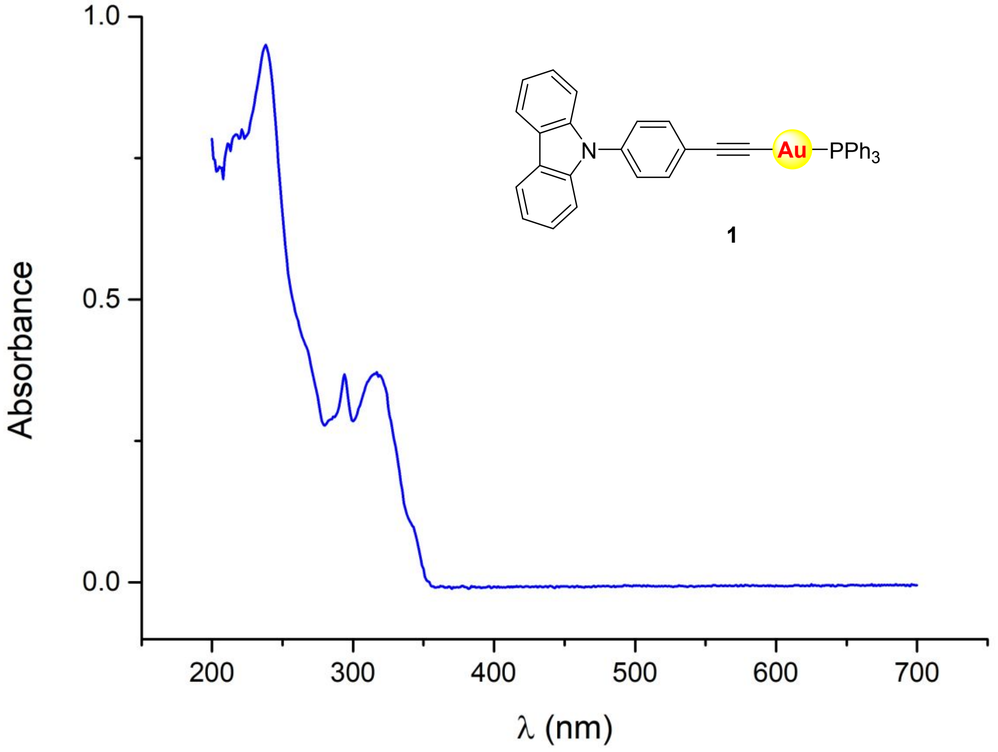

Figure S37. Normalized excitation and emission bands of complex **2**

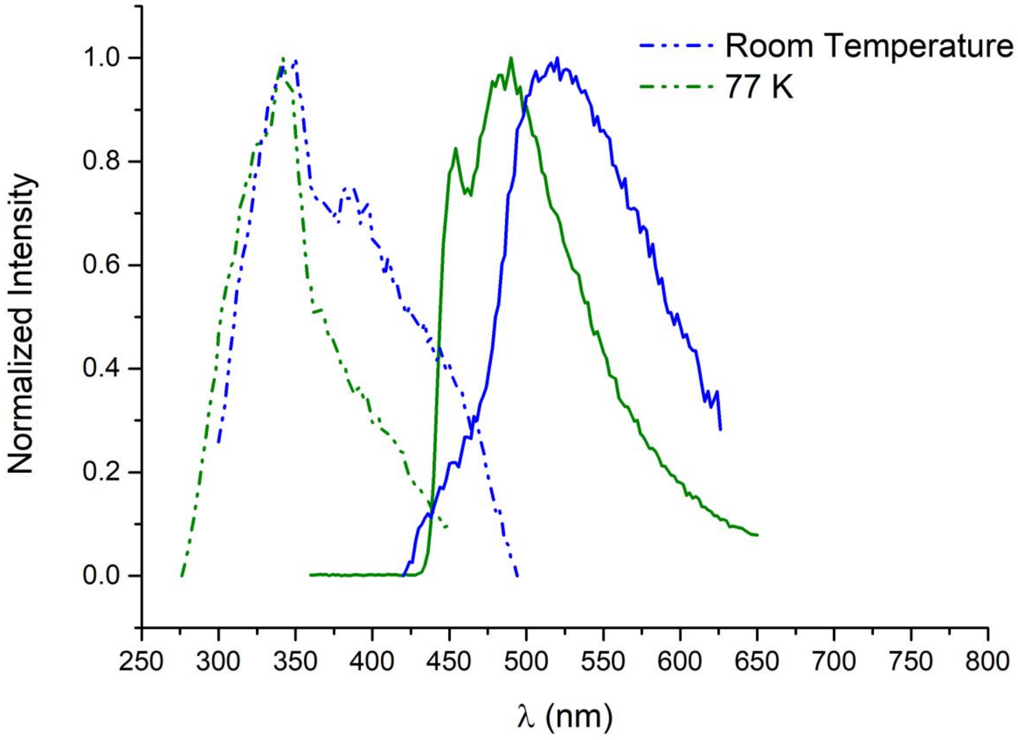

Figure S38. Absorption spectrum of gold complex **2**

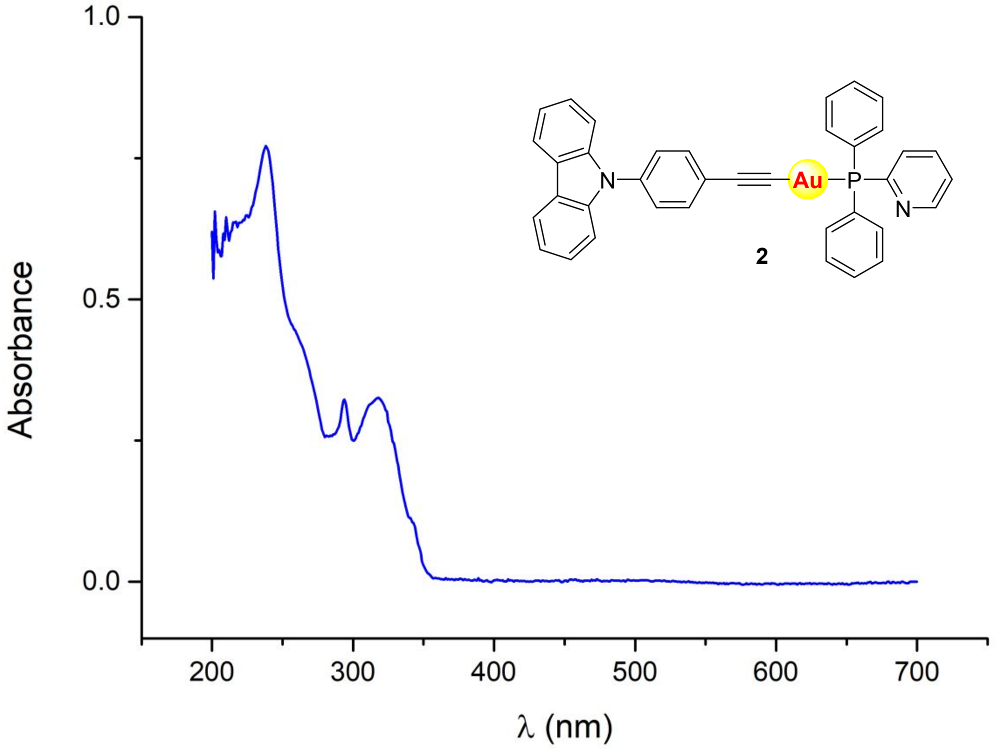

Figure S39. Normalized excitation and emission bands of gold complex **3**

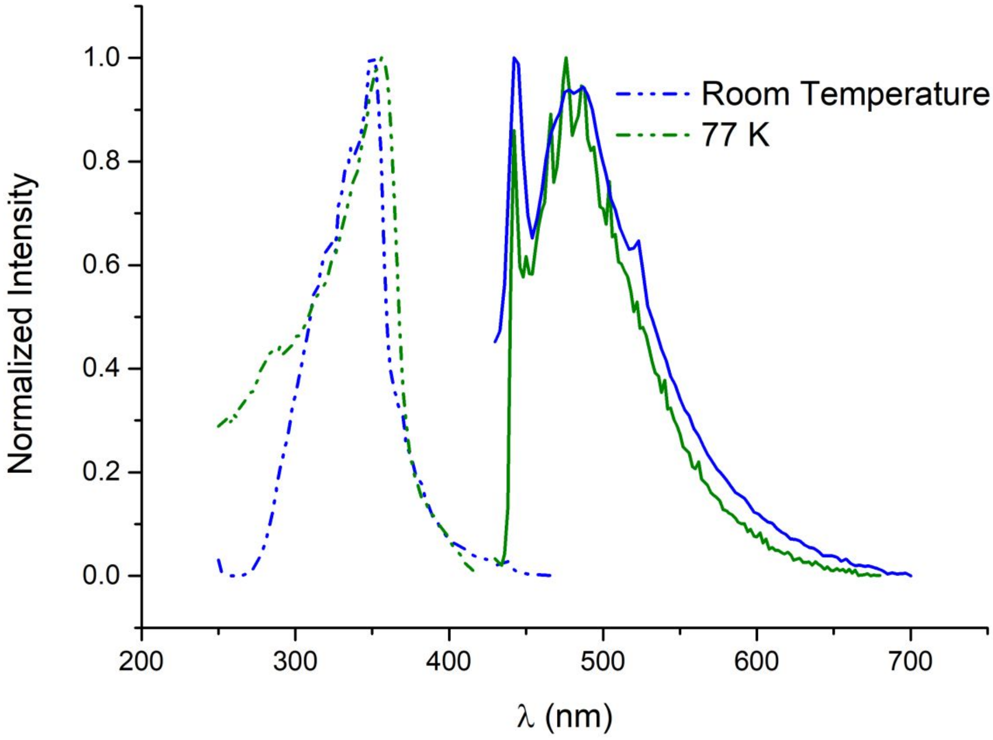

Figure S40. Absorption spectrum of gold complex **3**

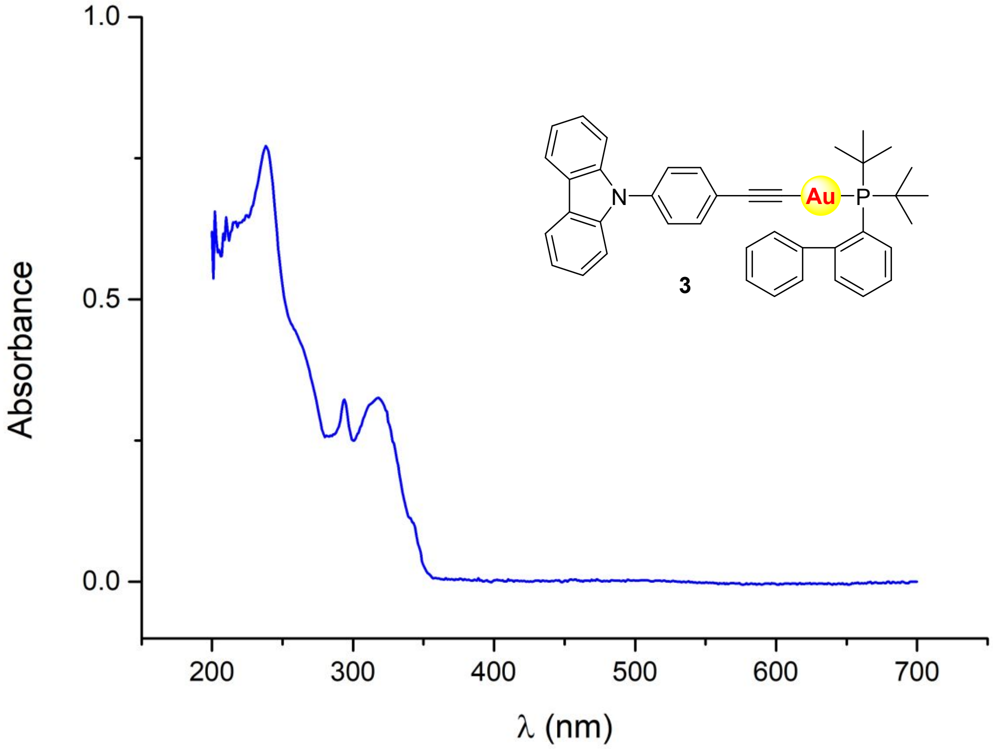

Figure S41. Normalized excitation and emission bands of gold complex **4**

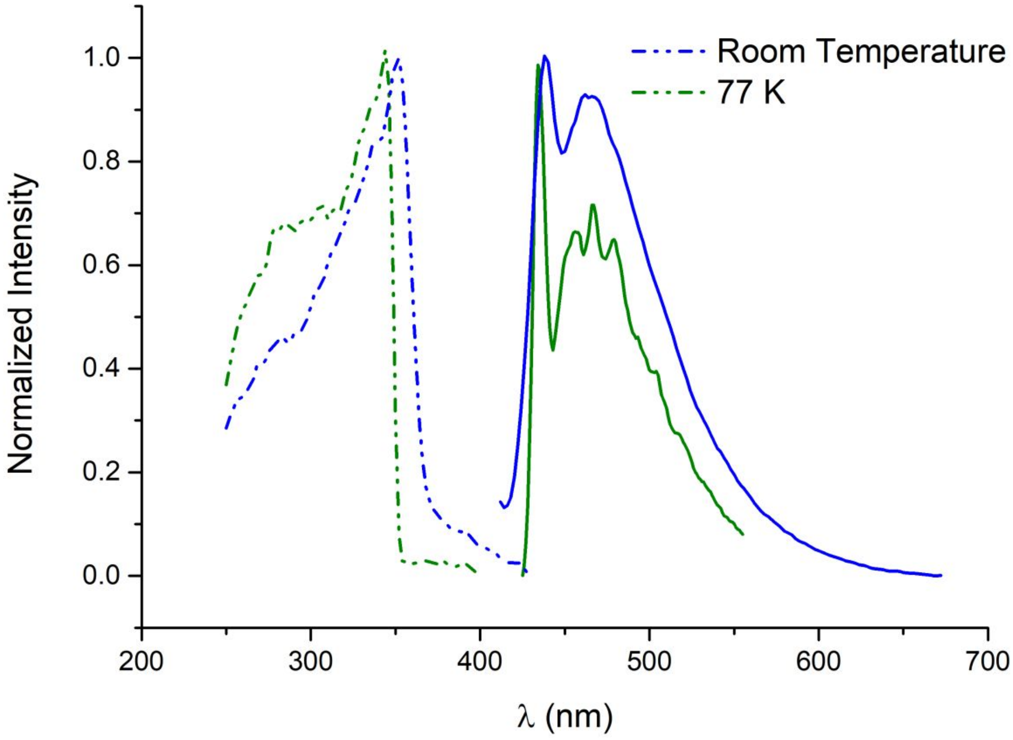

Figure S42. Absorption spectrum of gold complex **4**

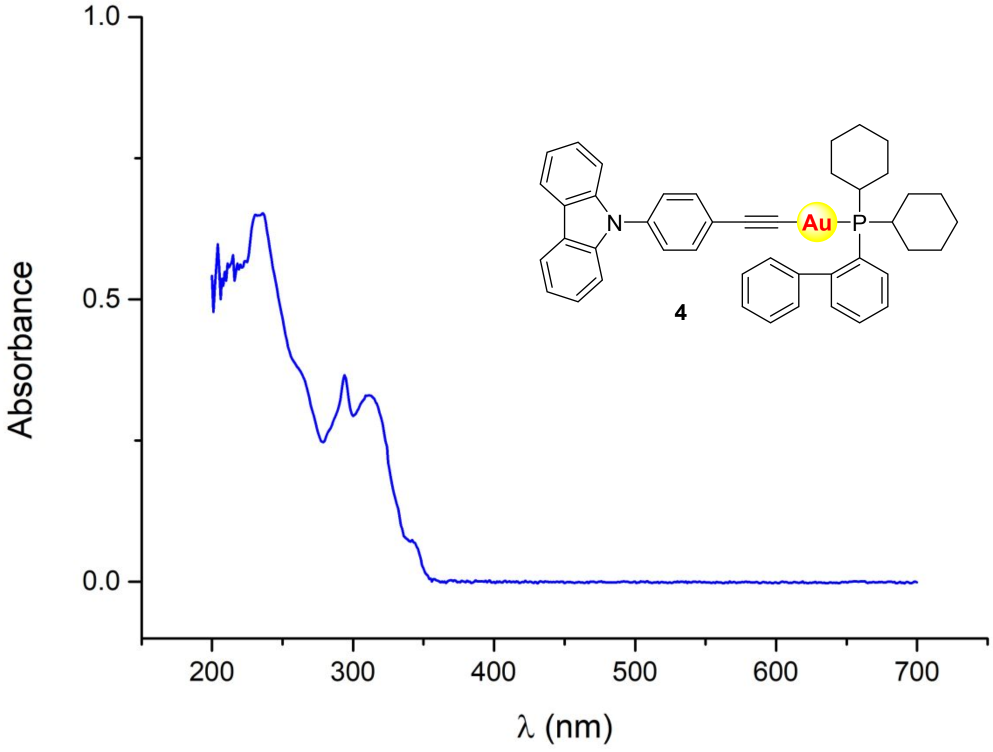

Figure S43. Normalized excitation and emission bands of gold complex **5**

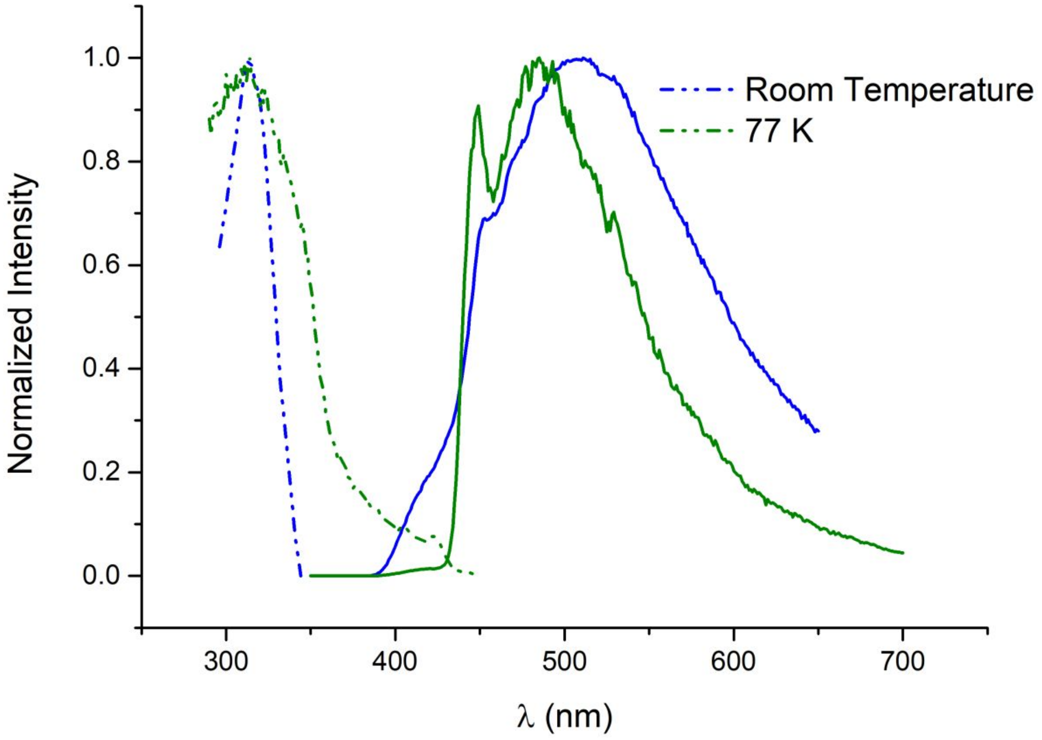

Figure S44. Absorption spectrum of gold complex **5**

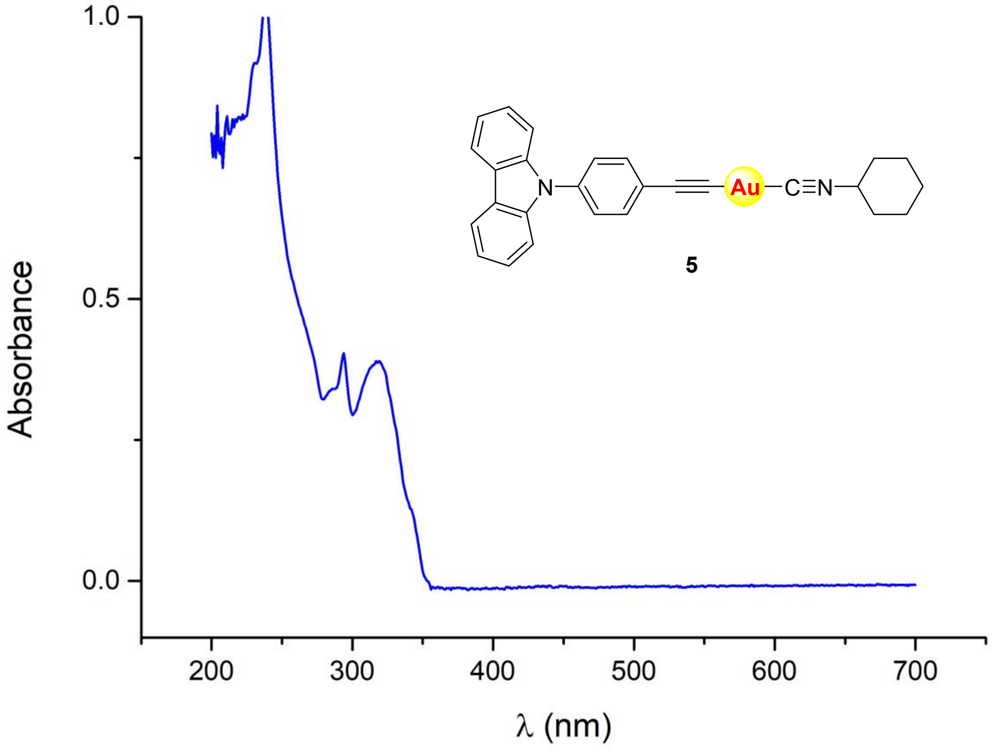

Figure S45. Normalized excitation and emission bands of gold complex **6**

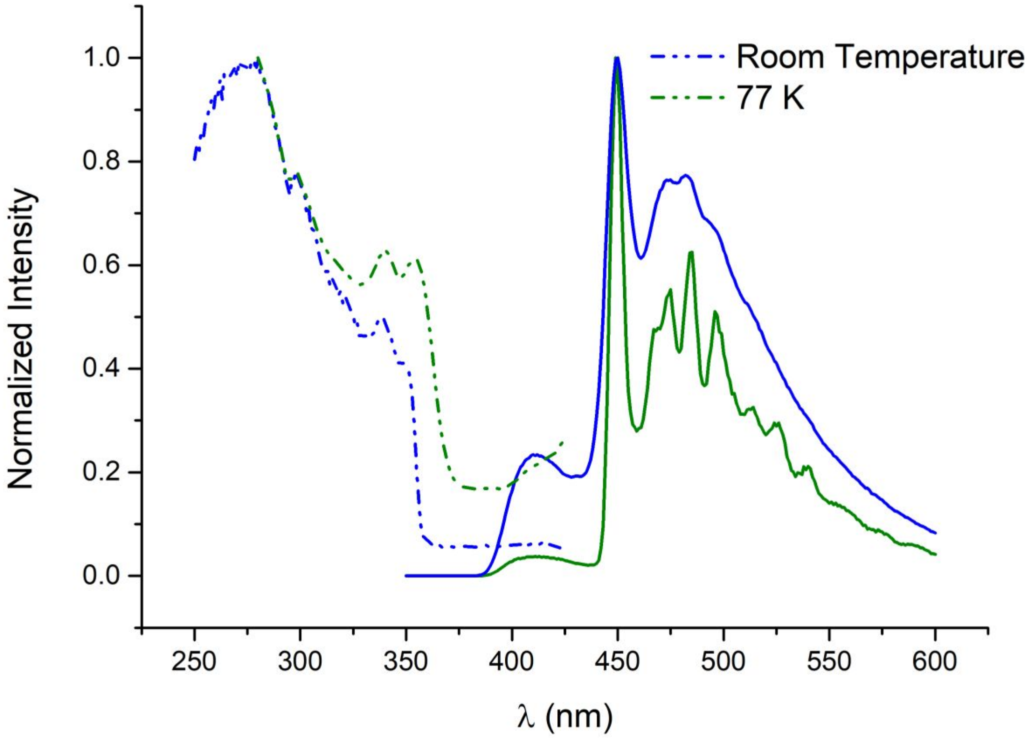

Figure S46. Absorption spectrum of gold complex **6**

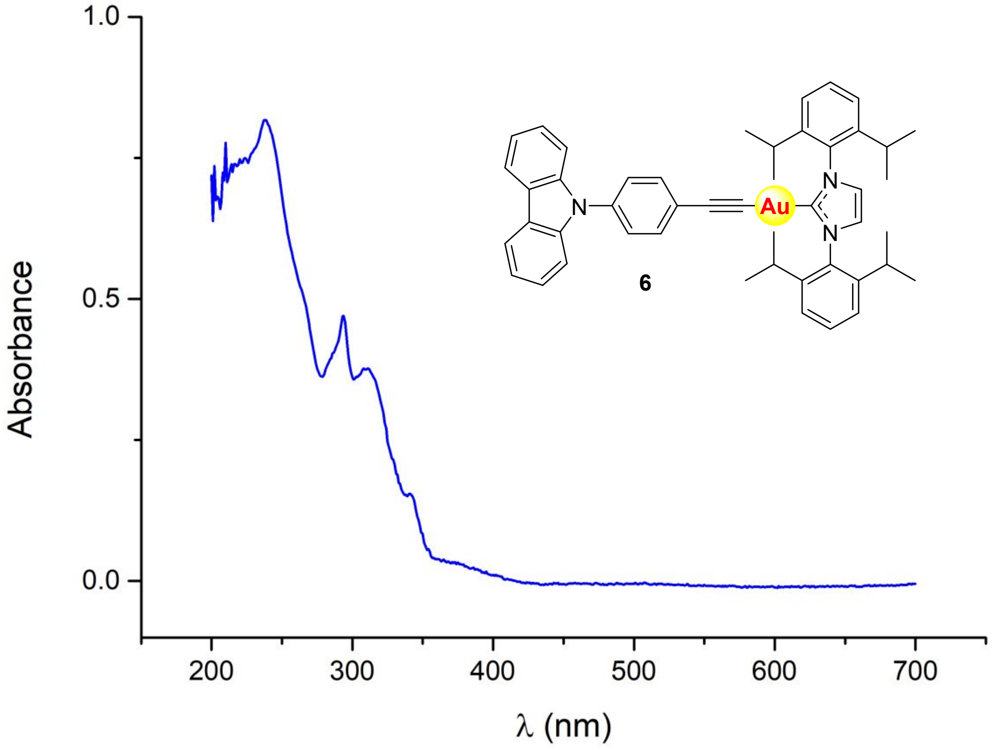

Figure S47. Normalized excitation and emission bands of gold complex **7**

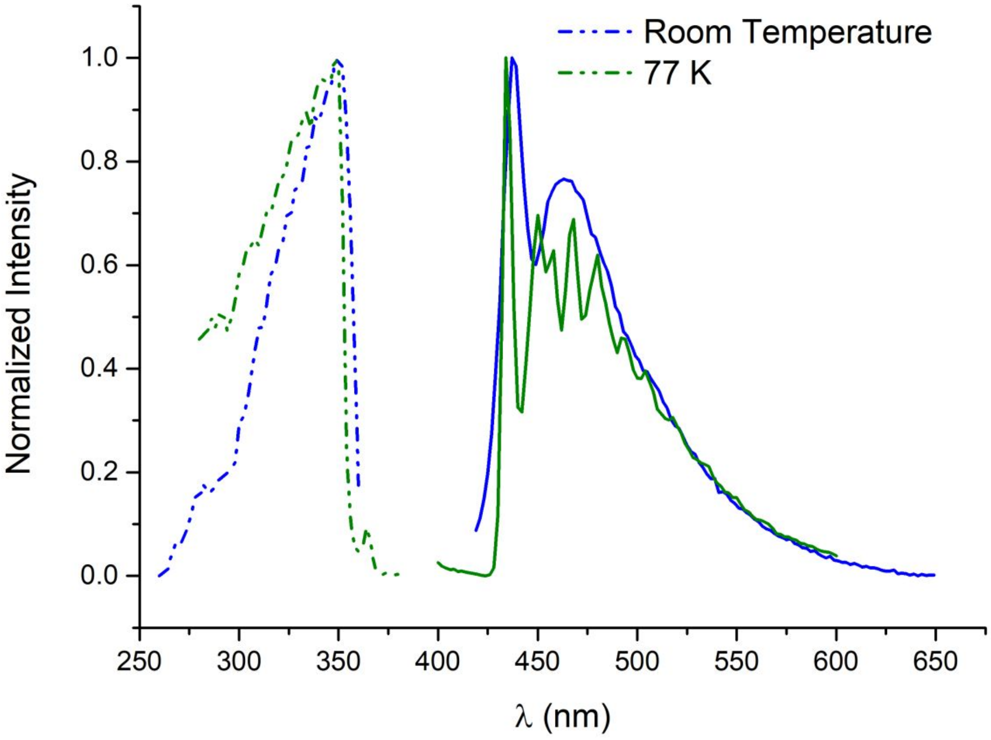

Figure S48. Absorption spectrum of gold complex **7**

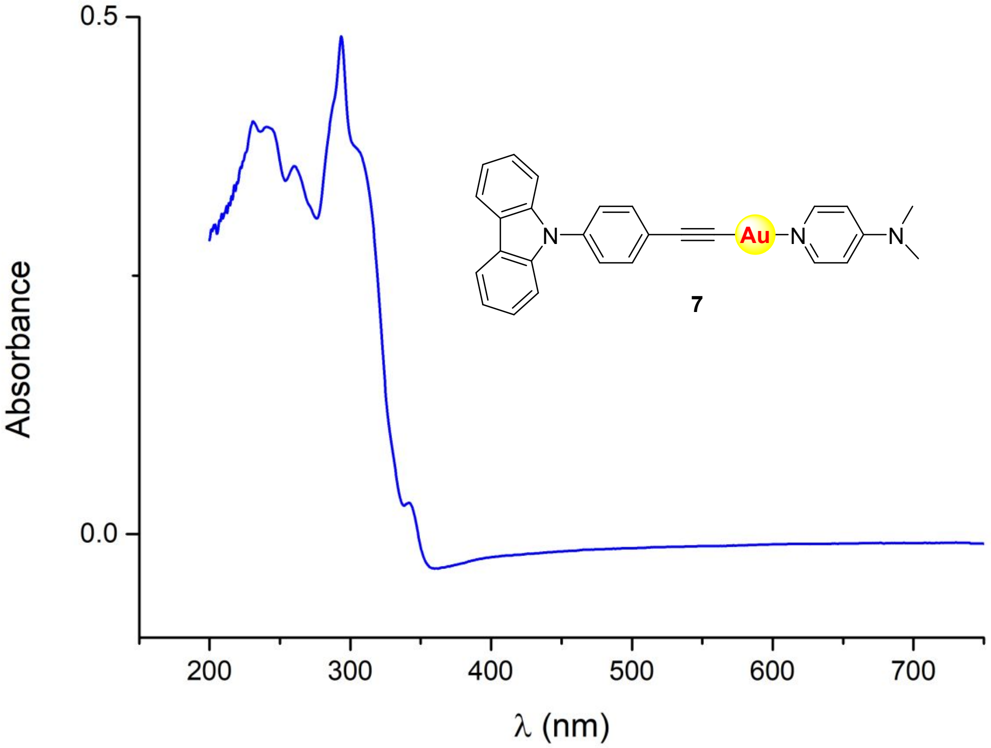

Figure S49. Normalized excitation and emission bands of gold complex **8**

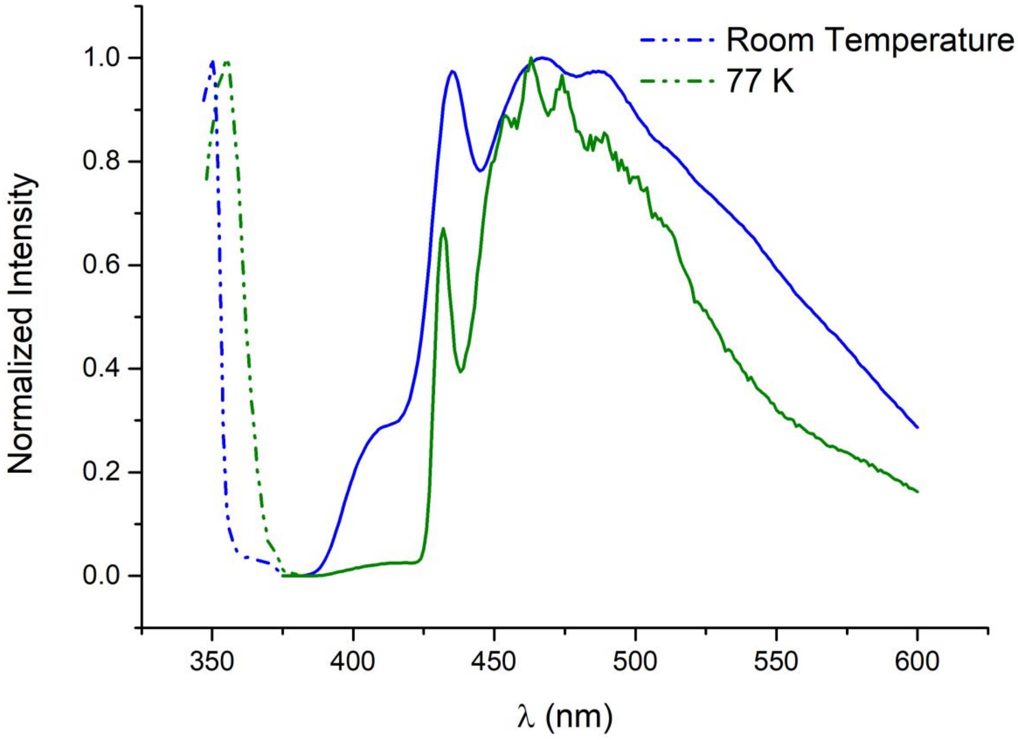

Figure S50. Absorption spectrum of gold complex **8**

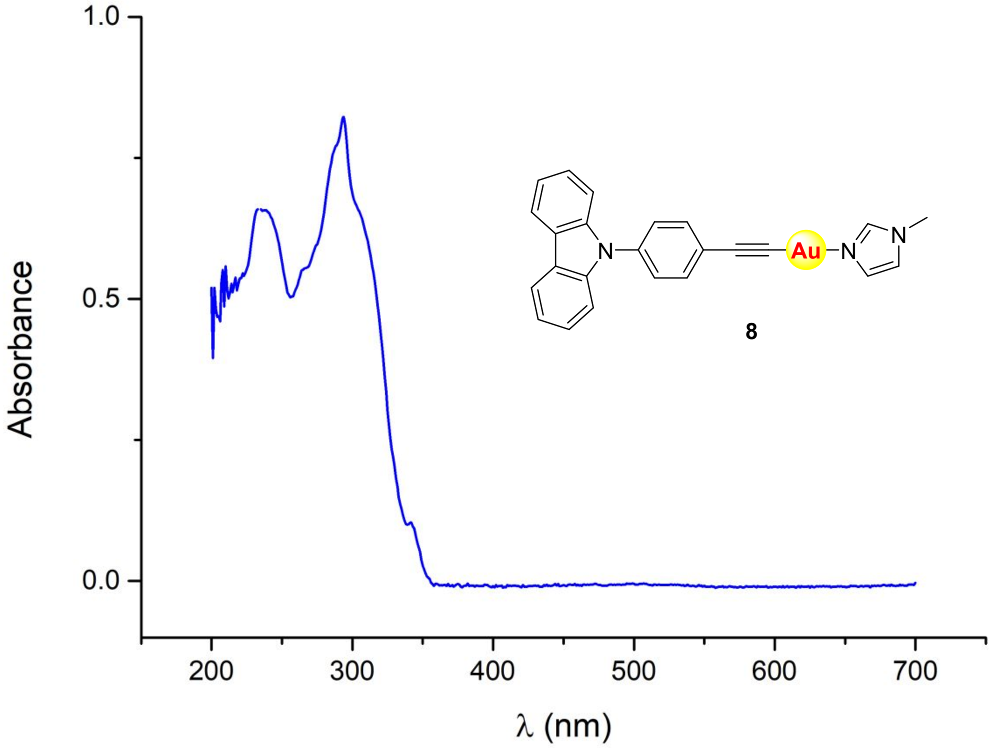

Figure S51. Normalized excitation and emission bands of gold complex **9**

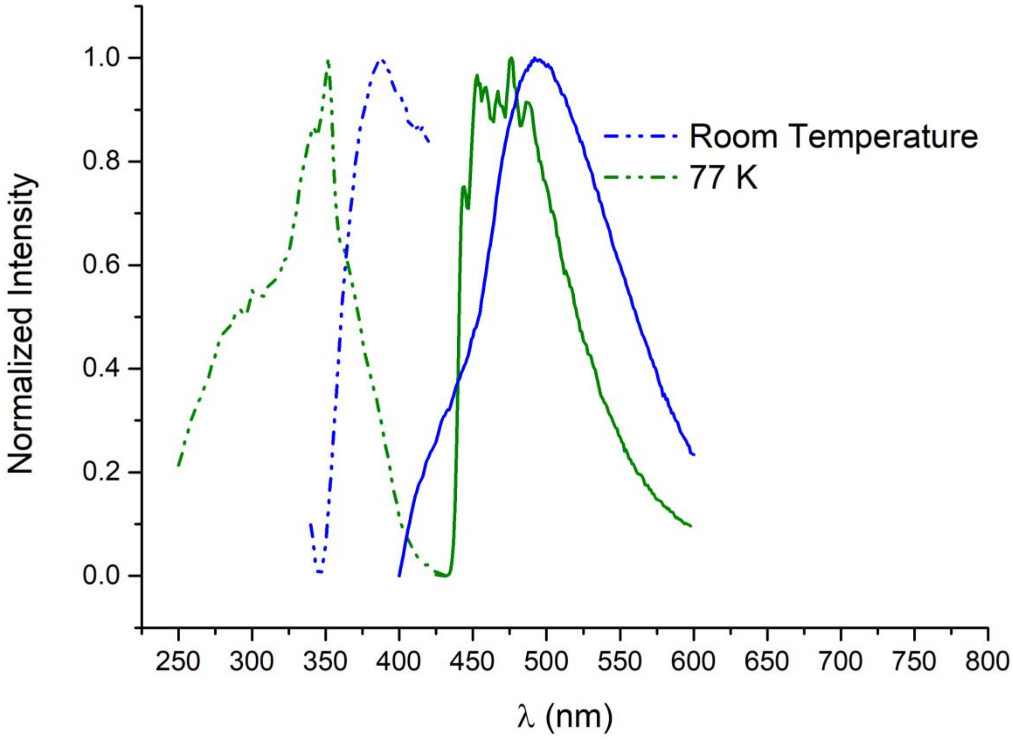

Figure S52. Absorption spectrum of gold complex **9**

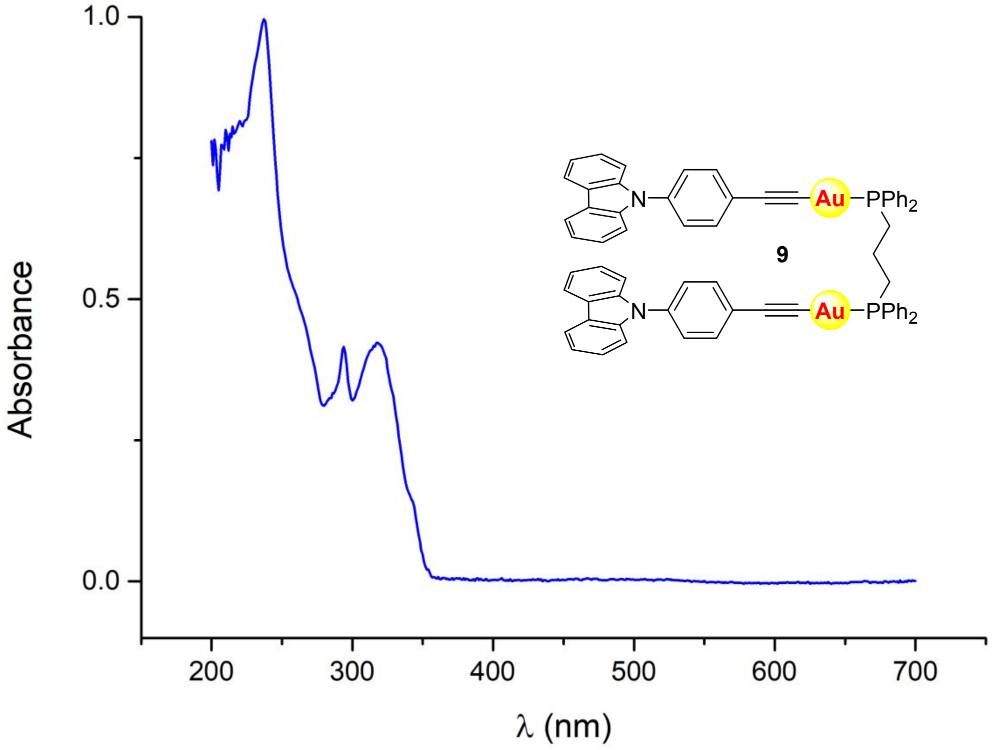

Figure S53. Normalized excitation and emission bands of gold complex **10**

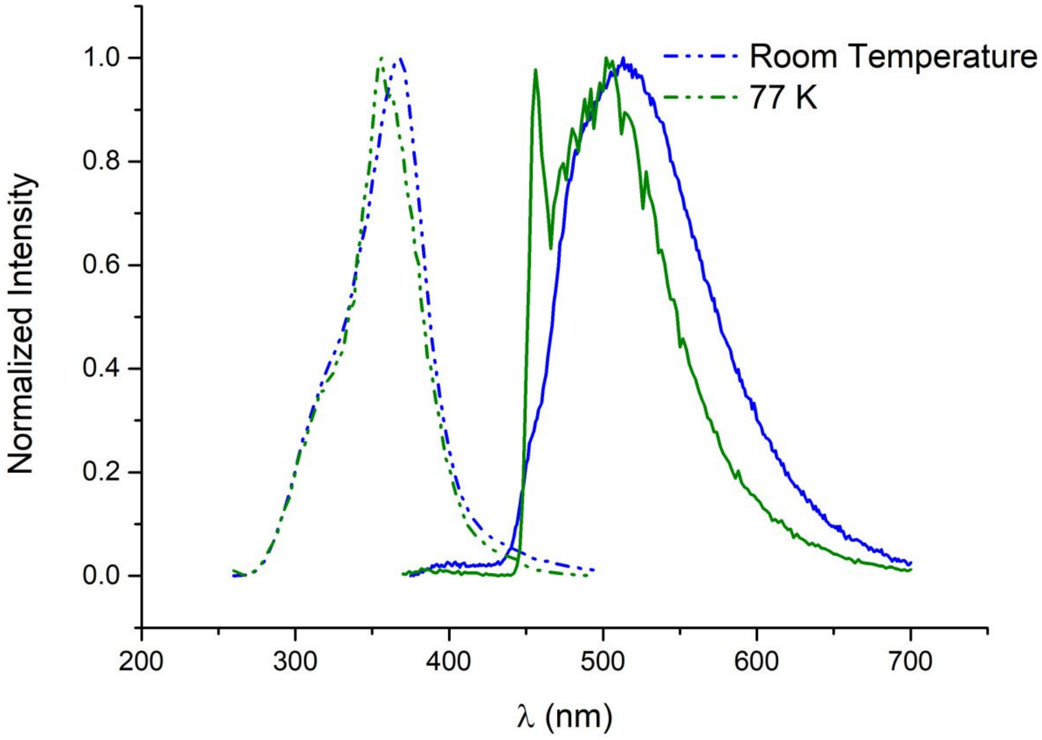

Figure S54. Absorption spectrum of gold complex **10**

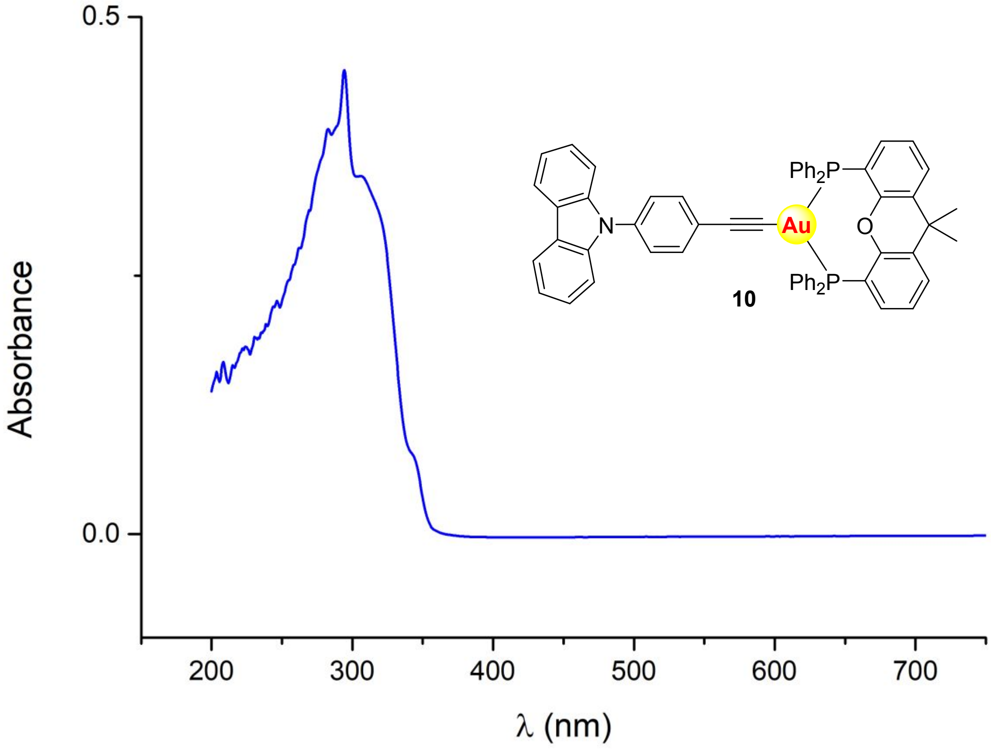

Figure S55. Normalized excitation and emission bands of gold complex **12**

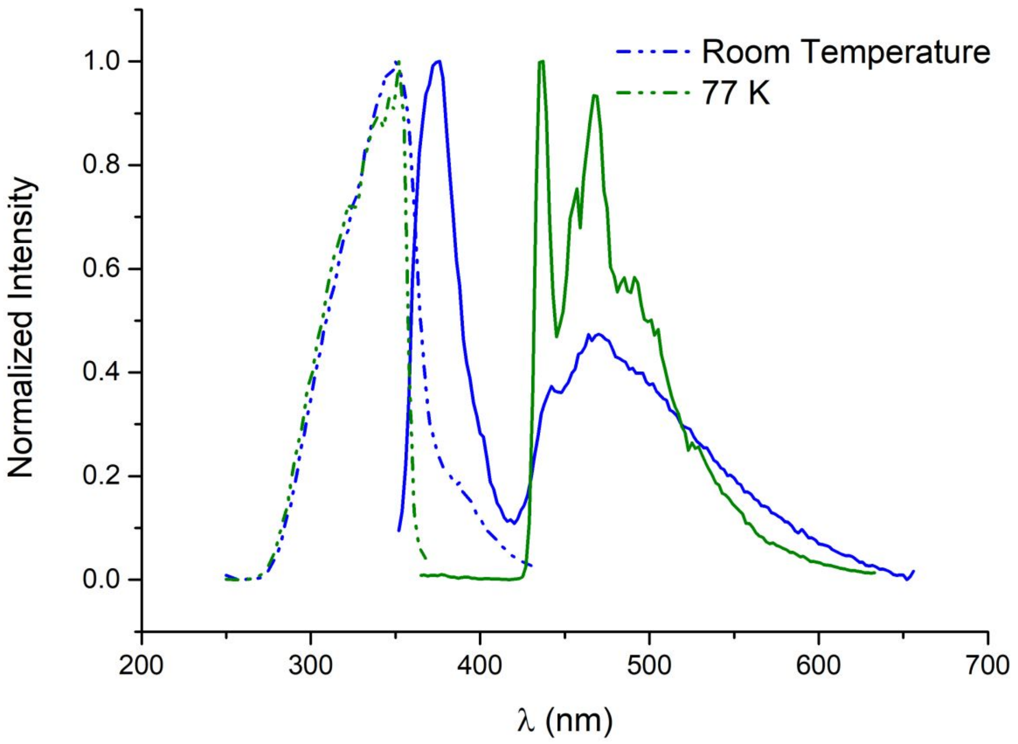

Figure S56. Absorption spectrum of gold complex **12**

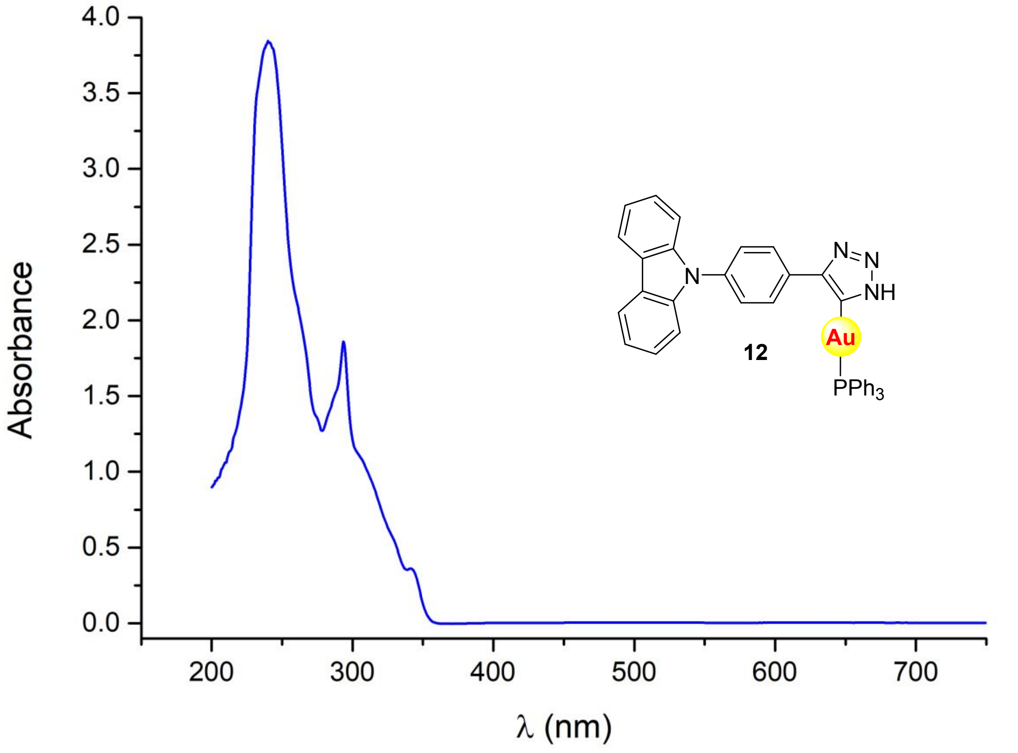

Figure S57. Normalized excitation and emission bands of gold complex **13**

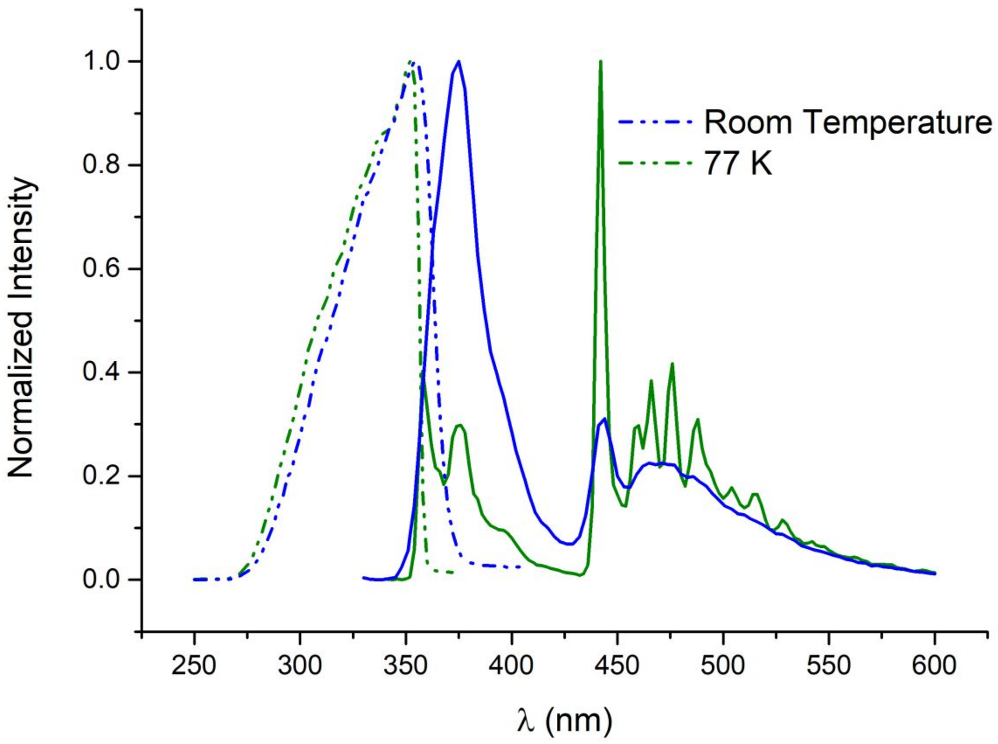

Figure S58. Absorption spectrum of gold complex **13**

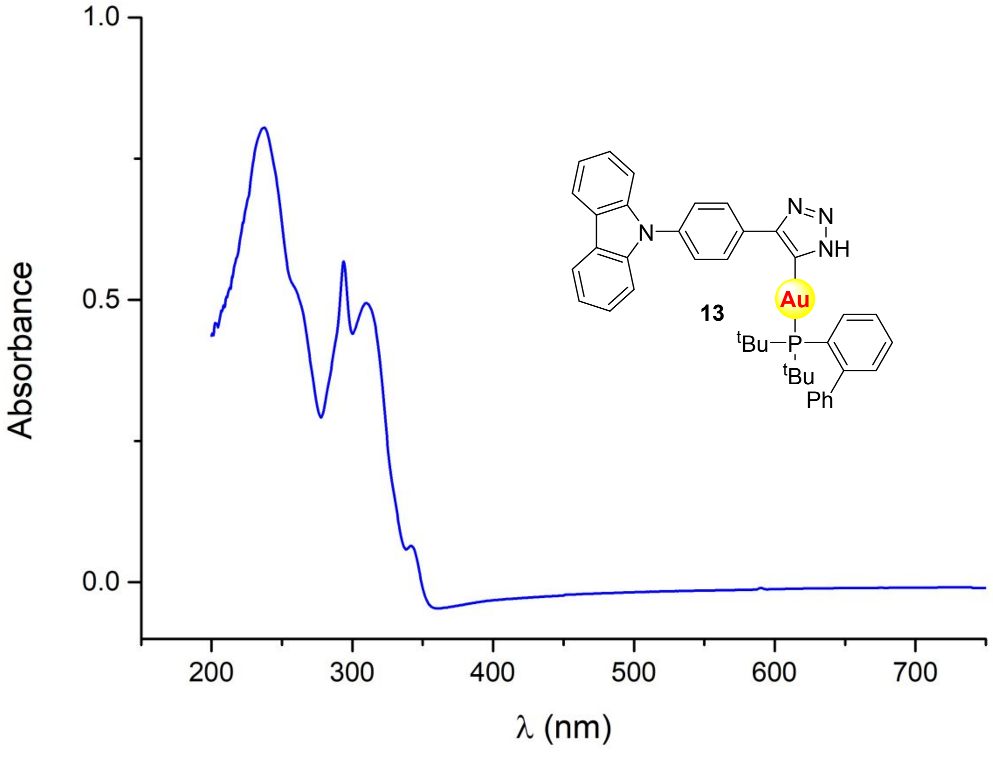

6. Complex 2 TADF graphic and lifetimes table

Figure S59. Lifetime (ms) vs. Temperature (K) plot for gold complex **2**. Experimental data (black dots) and calculated values (red line) using the Boltzmann-like equation shown below.

$$\tau = \frac{3 + e^{-\Delta E(S_1 - T_1)/K_B T}}{3\left(\frac{1}{\tau(T_1)}\right) + \left(\frac{1}{\tau(S_1)}\right) e^{-\Delta E(S_1 - T_1)/K_B T}}$$

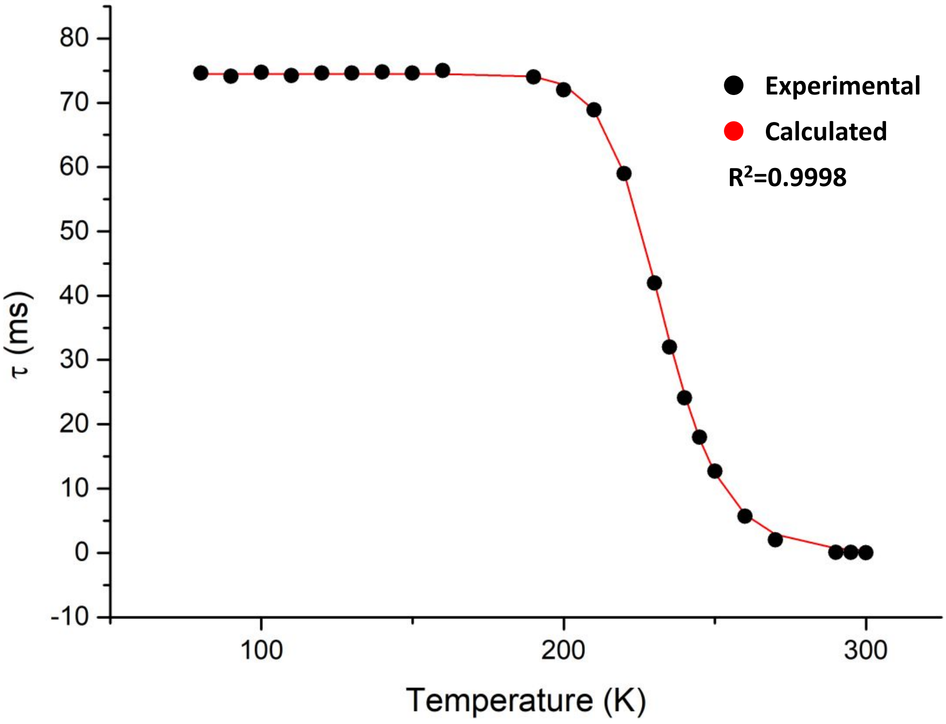

Table S1. Experimental and calculated lifetimes (ms) at each temperature value.

Temperature (K)	Experimental Lifetime (ms)	Calculated Lifetime (ms)
80	74.6	74.47
90	74.1	74.47
100	74.7	74.47
110	74.2	74.47
120	74.6	74.46
130	74.6	74.46
140	74.8	74.46
150	74.6	74.46
160	75	74.46
190	74	74.05
200	72	72.78
210	68.9	68.71
220	59	58.64
230	42	41.70
235	32	32.43
240	24.1	24.08

245	18	17.26
250	12.7	12.10
260	5.7	5.80
270	2	2.80
290	0.1	0.72
295	0.069	0.52
300	0.05	0.39

7. Computational methods

The ωB97X-D/6-31+G(d,p)[1,2,3,4] (genECP with Def2-SVP[5,6] for Au) combination was employed to optimize the geometries of stationary points. To confirm that the optimized geometries were energy minima, vibrational frequency calculations were carried out and no imaginary frequencies were observed. Calculations were done in gas phase.

TD-DFT calculations were performed with the Tamm-Dancoff approximation,[7] via the TDA keyword. Absorption was calculated with the vertical $S_0 \rightarrow S_1$ excitation using the optimized S_0 geometry. Emission was calculated with the vertical $T_1 \rightarrow S_0$ excitation using the T_1 geometry and including the 50-50 keyword ("tda=(50-50)").

Gaussian 16[8] was used to run all the density functional theory (DFT) calculations. AQME[9] was employed to i) generate tridimensional structures from SMILES strings and ii) identify and correct errors from geometry optimization and frequency DFT calculations in an automated manner. Molecular representations were created using PyMOL[10] with the display settings developed by Dr. Robert S. Paton from Colorado State University, which are openly accessible.[11]

Raw TD-DFT results

Absorption ($S_0 \rightarrow S_1$)

Excited State 1: Singlet-A 4.4251 eV 280.18 nm f=0.2353 <S**2>=0.000
146 ->154 -0.22958
147 ->164 -0.21866
148 ->154 0.58125 (major contribution)

Emission ($T_1 \rightarrow S_0$)

Excited State 1: Triplet-A 2.7933 eV 443.87 nm f=0.0000 <S**2>=2.000
138 ->181 -0.10355
148 ->152 0.67083 (major contribution)

XYZ molecular coordinates

68
S_0 Complex **1**

```
C       -9.04964360  -0.72272600   0.02949495
C       -9.04892461   0.72455810  -0.03107505
```

C	-10.05734869	1.68972017	-0.08254605
C	-9.70343865	3.03132226	-0.15227406
C	-8.35201156	3.41343730	-0.17714006
C	-7.33065348	2.47270824	-0.12672106
C	-7.69618754	1.12752114	-0.04717205
N	-6.88459045	-0.00007994	0.00099795
C	-5.46615137	-0.00074993	0.00156595
C	-4.76705930	-0.63814097	-1.02408912
C	-3.37789220	-0.64707397	-1.01769412
C	-2.65999615	-0.00187291	0.00244495
C	-1.22898405	-0.00230091	0.00267695
C	-0.00673596	-0.00295390	0.00275895
Au	1.99153019	-0.00169489	0.00184295
P	4.33091936	0.00059213	-0.00001405
C	5.07193642	-1.31141196	-1.03428412
C	6.22201249	-2.00638001	-0.64964710
C	6.76825454	-2.97280107	-1.49212715
C	6.17236751	-3.24771110	-2.72091824
C	5.02269642	-2.55963005	-3.10684927
C	4.47031337	-1.59937799	-2.26496121
C	5.07404741	-0.23780289	1.65251407
C	6.22403850	0.44422617	2.05985410
C	6.77283854	0.19867315	3.31704119
C	6.17956850	-0.72866092	4.17067725
C	5.02984842	-1.40819797	3.77002022
C	4.47487337	-1.16007195	2.51843013
C	5.07057440	1.55221524	-0.62089909
C	6.22595946	1.56656225	-1.40746115
C	6.77154851	2.77909534	-1.82456818
C	6.16960145	3.98056442	-1.45734615
C	5.01467838	3.97078342	-0.67629410
C	4.46301835	2.76169033	-0.26412107
C	-3.37799720	0.64381913	1.02220302
C	-4.76719230	0.63601012	1.02770403
C	-7.69730352	-1.12693602	0.04775396
C	-7.33312247	-2.47246712	0.12772496
C	-8.35542354	-3.41226420	0.17636796
C	-9.70645666	-3.02890417	0.14936996
C	-10.05903169	-1.68697308	0.07923396
H	-11.10240274	1.39404113	-0.07085505
H	-10.47569172	3.79288631	-0.19206806
H	-8.09737459	4.46710938	-0.23936106
H	-6.28793141	2.77139027	-0.15318906
H	-5.31888436	-1.12150301	-1.82446918
H	-2.83220115	-1.14482800	-1.81227918
H	6.69041855	-1.80047699	0.30804297
H	7.65869861	-3.51218711	-1.18489213
H	6.59836356	-4.00259615	-3.37443429
H	4.54917839	-2.77732807	-4.05876434

H 3.56482931 -1.07644096 -2.56183523
H 6.69013454 1.17092323 1.40135705
H 7.66324260 0.73552520 3.62880621
H 6.60757352 -0.91650793 5.15041732
H 4.55827839 -2.12401702 4.43555327
H 3.56928731 -1.67958700 2.21588311
H 6.69892951 0.63421819 -1.70073817
H 7.66612360 2.78266935 -2.43934722
H 6.59492549 4.92379350 -1.78570318
H 4.53630834 4.90381748 -0.39642808
H 3.55330528 2.75728832 0.33077898
H -2.83240416 1.14101817 1.81721408
H -5.31913937 1.11978515 1.82775208
H -6.29072240 -2.77212013 0.15579996
H -8.10185249 -4.46617927 0.23883297
H -10.47946968 -3.78976723 0.18780497
H -11.10379474 -1.39033906 0.06586396

68
T_1 Complex **1**
C -9.07303400 -0.67804200 0.02485600
C -9.07109700 0.68316600 -0.02792100
C -10.12652100 1.68314300 -0.07732300
C -9.76481100 3.01046500 -0.15453400
C -8.41203400 3.40926700 -0.18485300
C -7.34020000 2.45094400 -0.13035900
C -7.67797500 1.13617900 -0.04912100
N -6.87266400 -0.00045300 0.00205400
C -5.45509000 -0.00233400 0.00299000
C -4.75641500 -0.64859500 -1.01744100
C -3.36733100 -0.65796500 -1.01086800
C -2.64919900 -0.00520800 0.00428400
C -1.21814300 -0.00612800 0.00449600
C 0.00411700 -0.00683500 0.00429100
Au 2.00227100 -0.00439600 0.00272600
P 4.34152900 0.00064400 -0.00028300
C 5.08421900 -1.29921500 -1.04867000
C 6.23671400 -1.99486700 -0.67261400
C 6.78432900 -2.95144500 -1.52535900
C 6.18735600 -3.21584100 -2.75592900
C 5.03526500 -2.52710200 -3.13334700
C 4.48154400 -1.57666500 -2.28123400
C 5.08625600 -0.25406500 1.64911900
C 6.23499800 0.42602400 2.06312100
C 6.78518200 0.16806000 3.31722300
C 6.19452200 -0.76979400 4.16111500
C 5.04601100 -1.44744900 3.75382700
C 4.48964700 -1.18696700 2.50536500
C 5.07878900 1.55978900 -0.60505000

C	6.23329900	1.58403500	-1.39263000
C	6.77708500	2.80167000	-1.79706600
C	6.17422100	3.99838000	-1.41608900
C	5.02012800	3.97876400	-0.63398000
C	4.47025300	2.76461900	-0.23446900
C	-3.36690800	0.64880100	1.01892800
C	-4.75603300	0.64229500	1.02418700
C	-7.68120100	-1.13486100	0.05036500
C	-7.34728800	-2.45060600	0.13225300
C	-8.42185000	-3.40595200	0.18307000
C	-9.77346600	-3.00343600	0.14864800
C	-10.13130800	-1.67513500	0.07072300
H	-11.16621300	1.37614500	-0.05576600
H	-10.53591500	3.77403800	-0.19438000
H	-8.16816600	4.46417800	-0.25082000
H	-6.30591500	2.77476200	-0.15899200
H	-5.30832900	-1.14026200	-1.81256500
H	-2.82173500	-1.16243700	-1.80129700
H	6.70599500	-1.79715200	0.28636900
H	7.67667700	-3.49141500	-1.22474500
H	6.61439000	-3.96309300	-3.41749200
H	4.56089900	-2.73668000	-4.08666100
H	3.57422400	-1.05326200	-2.57160200
H	6.69904000	1.16085000	1.41222500
H	7.67461400	0.70346100	3.63422500
H	6.62357500	-0.96733500	5.13849200
H	4.57641700	-2.17146100	4.41186500
H	3.58495300	-1.70508000	2.19778000
H	6.70695400	0.65546500	-1.69657200
H	7.67098700	2.81294800	-2.41273300
H	6.59815400	4.94561800	-1.73456600
H	4.54100700	4.90814000	-0.34343300
H	3.56115000	2.75268100	0.36125800
H	-2.82100500	1.15208300	1.80991300
H	-5.30768900	1.13511000	1.81878000
H	-6.31398900	-2.77727300	0.16395900
H	-8.18109200	-4.46155100	0.24945300
H	-10.54676300	-3.76492400	0.18583200
H	-11.17008700	-1.36529800	0.04597800

8. Bibliography

1. Becke, A. D. Density-functional thermochemistry. V. Systematic optimization of exchange-correlation functionals. *J. Chem. Phys.* **1997**, *107*, 8554-8560.

2. Chai, J. D.; Head-Gordon, M. Long-range corrected hybrid density functionals with damped atom-atom dispersion corrections. *Phys. Chem. Chem. Phys.* **2008**, *10*, 6615-6620.

3. Hehre, W. J.; Ditchfield, R.; Pople, J. A. Self—Consistent Molecular Orbital Methods. XII. Further Extensions of Gaussian—Type Basis Sets for Use in Molecular Orbital Studies of Organic Molecules. *The J. Chem. Phys.* **1972**, *56*, 2257-2261.

[4] Krishnan, R.; Binkley, J. S.; Seeger, R.; Pople, J. A. Self-consistent molecular orbital methods. XX. A basis set for correlated wave functions. *J. Chem. Phys.* **1980**, *72*, 650-654.

[5] Weigend, F.; Ahlrichs, R. Balanced basis sets of split valence, triple zeta valence and quadruple zeta valence quality for H to Rn: Design and assessment of accuracy. *Phys. Chem. Chem. Phys.* **2005**, *7*, 3297-3305.

[6] Weigend, F. Accurate Coulomb-fitting basis sets for H to Rn. *Phys. Chem. Chem. Phys.* **2006**, *8*, 1057-1065.

[7] Hirata, S.; Head-Gordon, M. Time-dependent density functional theory within the Tamm–Dancoff approximation. *Chem. Phys. Lett.* **1999**, *314*, 291-299.

[8] Gaussian 16 Rev. C.01, Frisch, M. J.; Trucks, G. W.; Schlegel, H. B.; Scuseria, G. E.; Robb, M. A.; Cheeseman, J. R.; Scalmani, G.; Barone, V.; Petersson, G. A.; Nakatsuji, H.; Li, X.; Caricato, M.; Marenich, J.; Bloino, A.; Janesko, B. G.; Gomperts, R.; Mennucci, B.; Hratchian, H. P.; Ortiz, J. V.; Izmaylov, A. F.; Sonnenberg, J. L.; Williams-Young, D.; Ding, F.; Lipparini, F.; Egidi, F.; Goings, J.; Peng, B.; Petrone, A.; Henderson, T.; Ranasinghe, D.; Zakrzewski, V. G.; Gao, J.; Rega, N.; Zheng, G.; Liang, W.; Hada, M.; Ehara, M.; Toyota, K.; Fukuda, R.; Hasegawa, J.; Ishida, M.; Nakajima, T.; Honda, Y.; Kitao, O.; Nakai, H.; Vreven, T.; Throssell, K.; Montgomery, Jr., J. A.; Peralta, J. E.; Ogliaro, F.; Bearpark, M.; Heyd, J. J.; Brothers, E.; Kudin, K. N.; Staroverov, V. N.; Keith, T.; Kobayashi, R.; Normand, J.; Raghavachari, K.; Rendell, A.; Burant, J. C.; Iyengar, S. S.; Tomasi, J.; Cossi, M.; Millam, J. M.; Klene, M.; Adamo, C.; Cammi, R.; Ochterski, J. W.; Martin, R. L.; Morokuma, K.; Farkas, O.; Foresman, J. B.; Fox, D. J. Gaussian, Inc., Wallingford CT, 2016.

[9] Alegre-Requena, J. V.; Shree, S. S.; Pérez-Soto, R.; Alturaifi, T. M.; Paton, R. S. AQME: Automated quantum mechanical environments for researchers and educators. *Wires Comput. Mol. Sci.* **2023**, *13*, e1663.

[10] Schrodinger, L. The PyMOL molecular graphics system.

[11] https://gist.github.com/bobbypaton (accessed 09 July 2021).
